# Supplementary figures and images for: Gim3 buffers and potentiates de novo mutations that affect fluconazole susceptibility in yeast (part 1 of 2)
Source: EMBO Rep. 2026 Feb 17;27(6):1510–39. doi: 10.1038/s44319-026-00702-x (PMC13022404; doi:10.1038/s44319-026-00702-x)

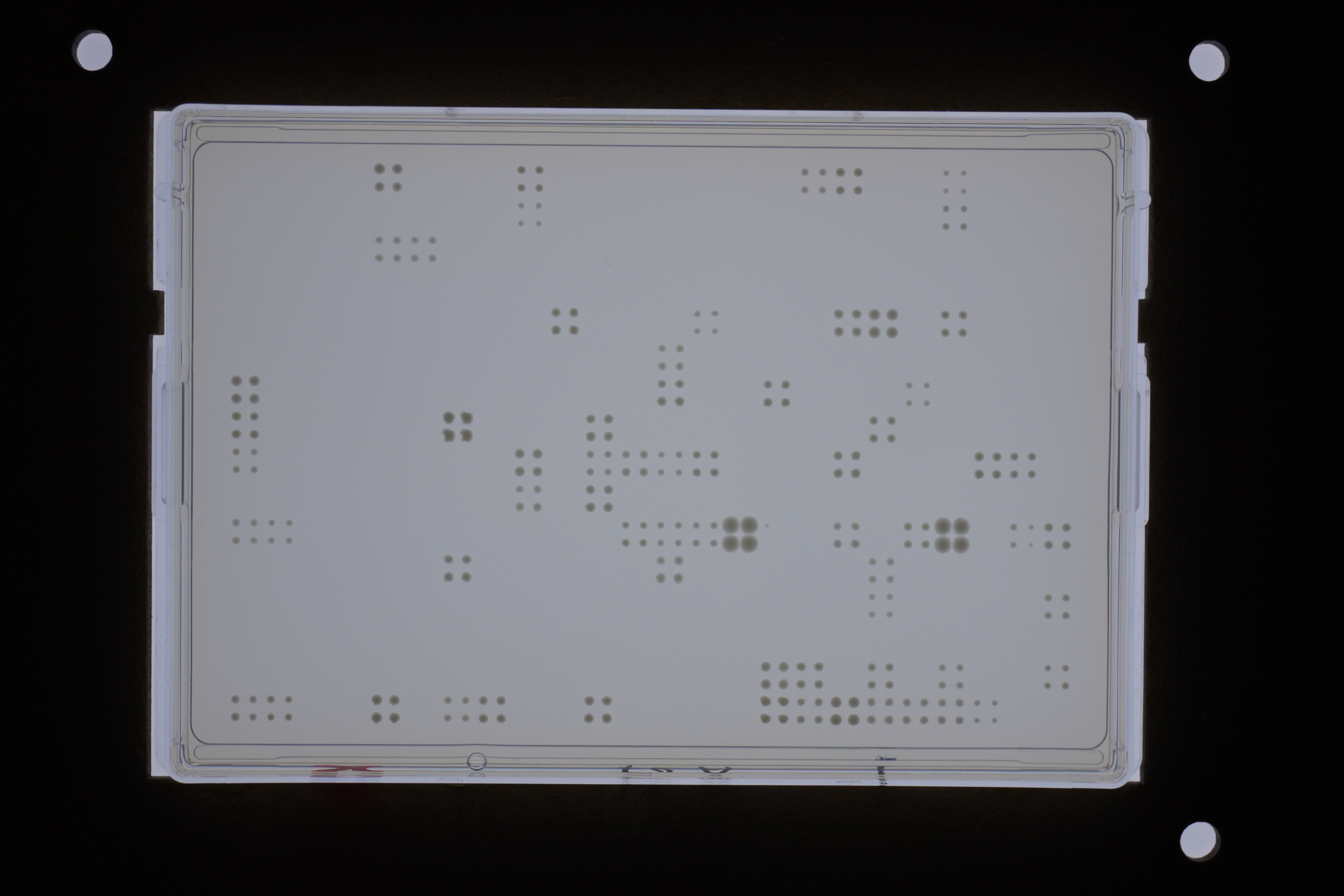

Supplement: Supplementary file 14 — Source data Fig. 2 [file 44319_2026_702_MOESM14_ESM.zip › Figure2B_SourceData/Images/SC_5FOA_1.TIFF]

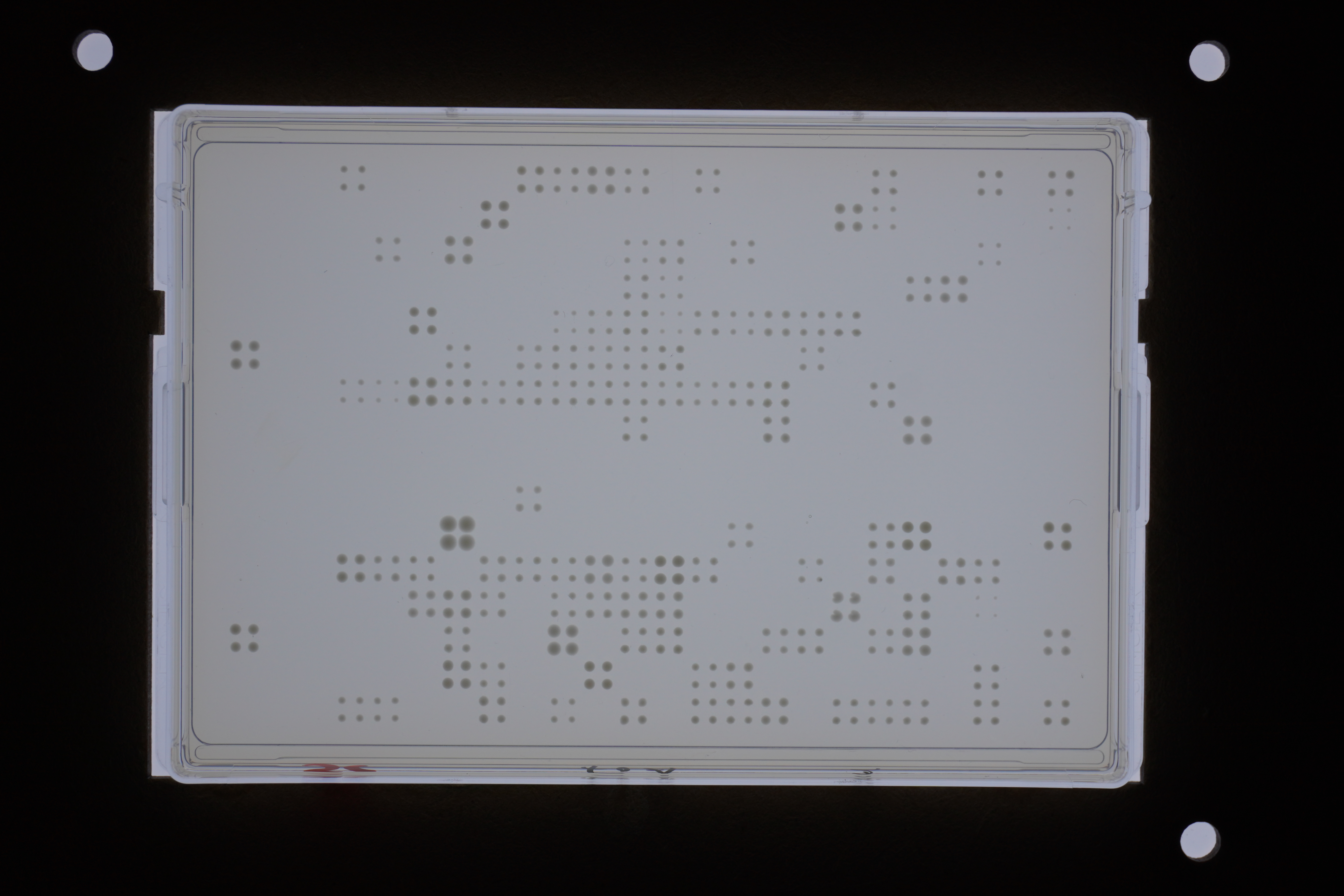

Supplement: Supplementary file 14 — Source data Fig. 2 [file 44319_2026_702_MOESM14_ESM.zip › Figure2B_SourceData/Images/SC_5FOA_2.TIFF]

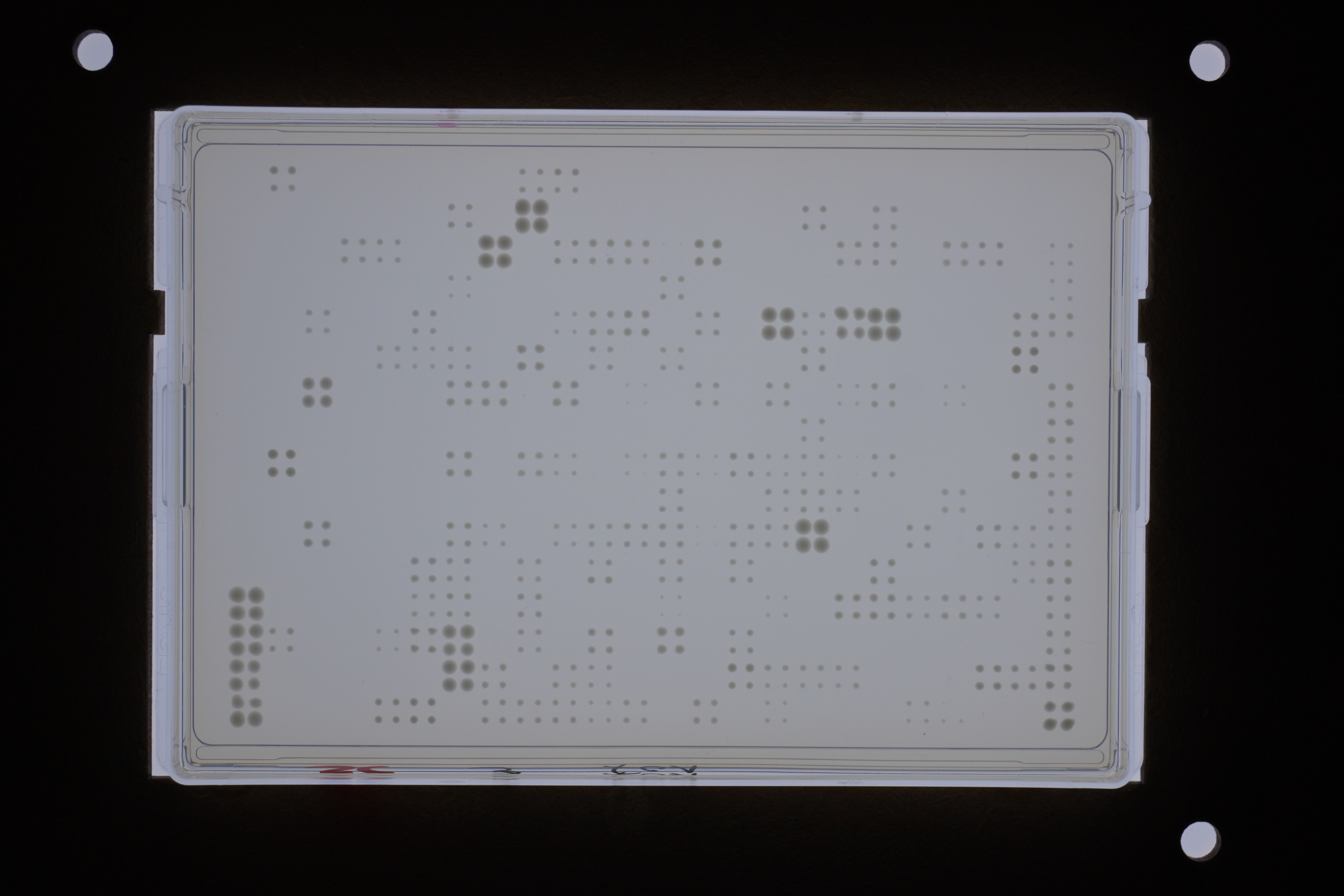

Supplement: Supplementary file 14 — Source data Fig. 2 [file 44319_2026_702_MOESM14_ESM.zip › Figure2B_SourceData/Images/SC_5FOA_3.TIFF]

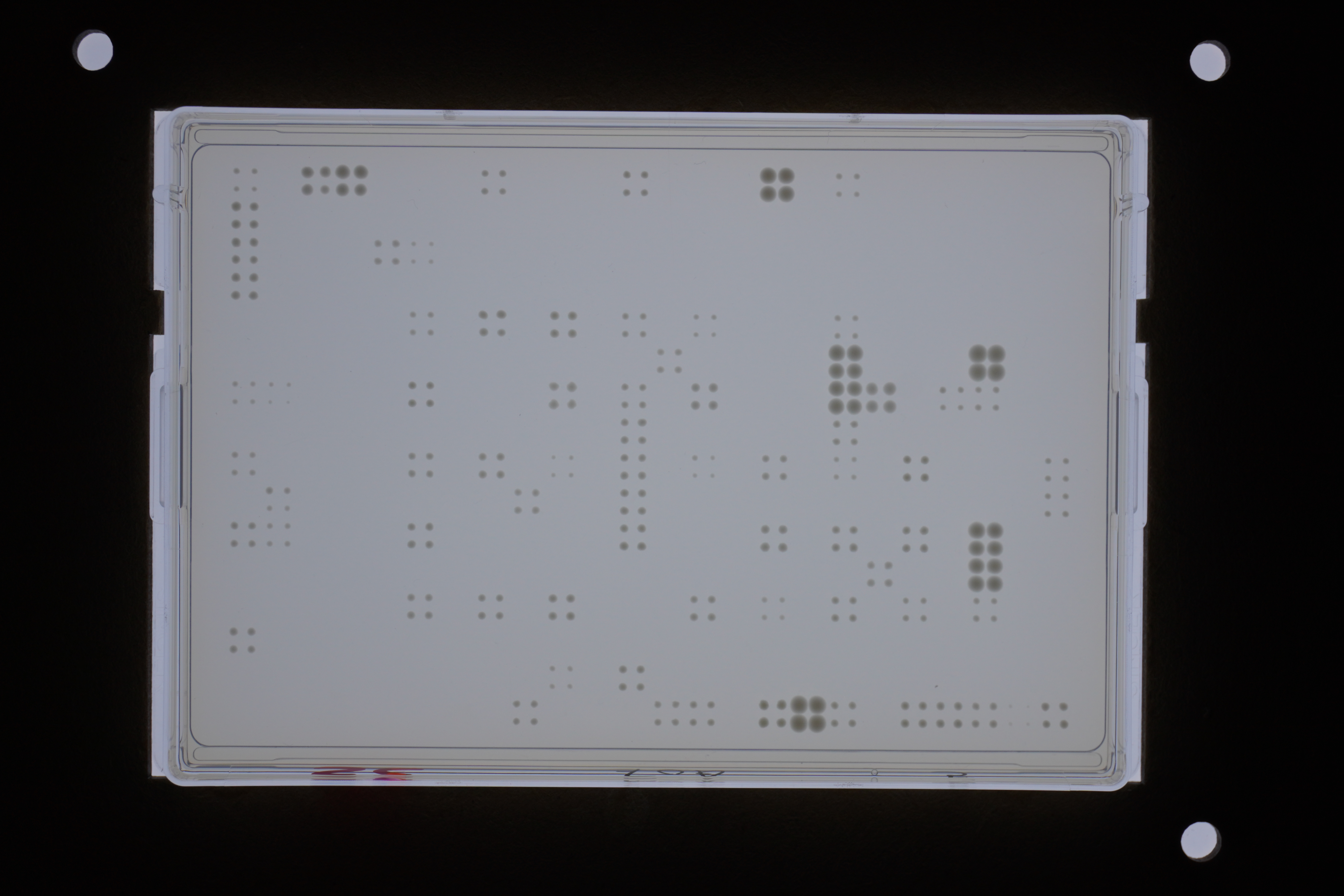

Supplement: Supplementary file 14 — Source data Fig. 2 [file 44319_2026_702_MOESM14_ESM.zip › Figure2B_SourceData/Images/SC_5FOA_4.TIFF]

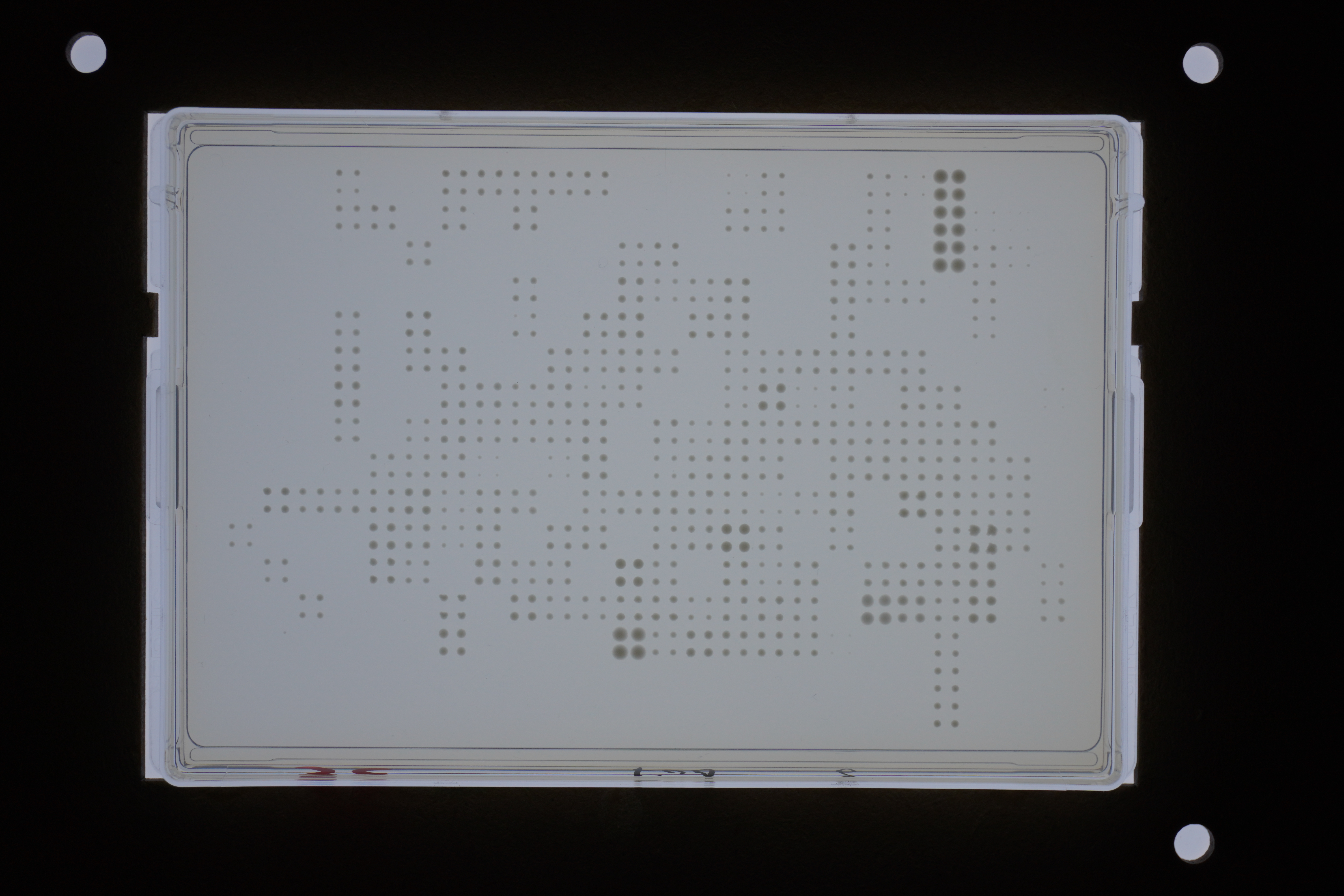

Supplement: Supplementary file 14 — Source data Fig. 2 [file 44319_2026_702_MOESM14_ESM.zip › Figure2B_SourceData/Images/SC_5FOA_5.TIFF]

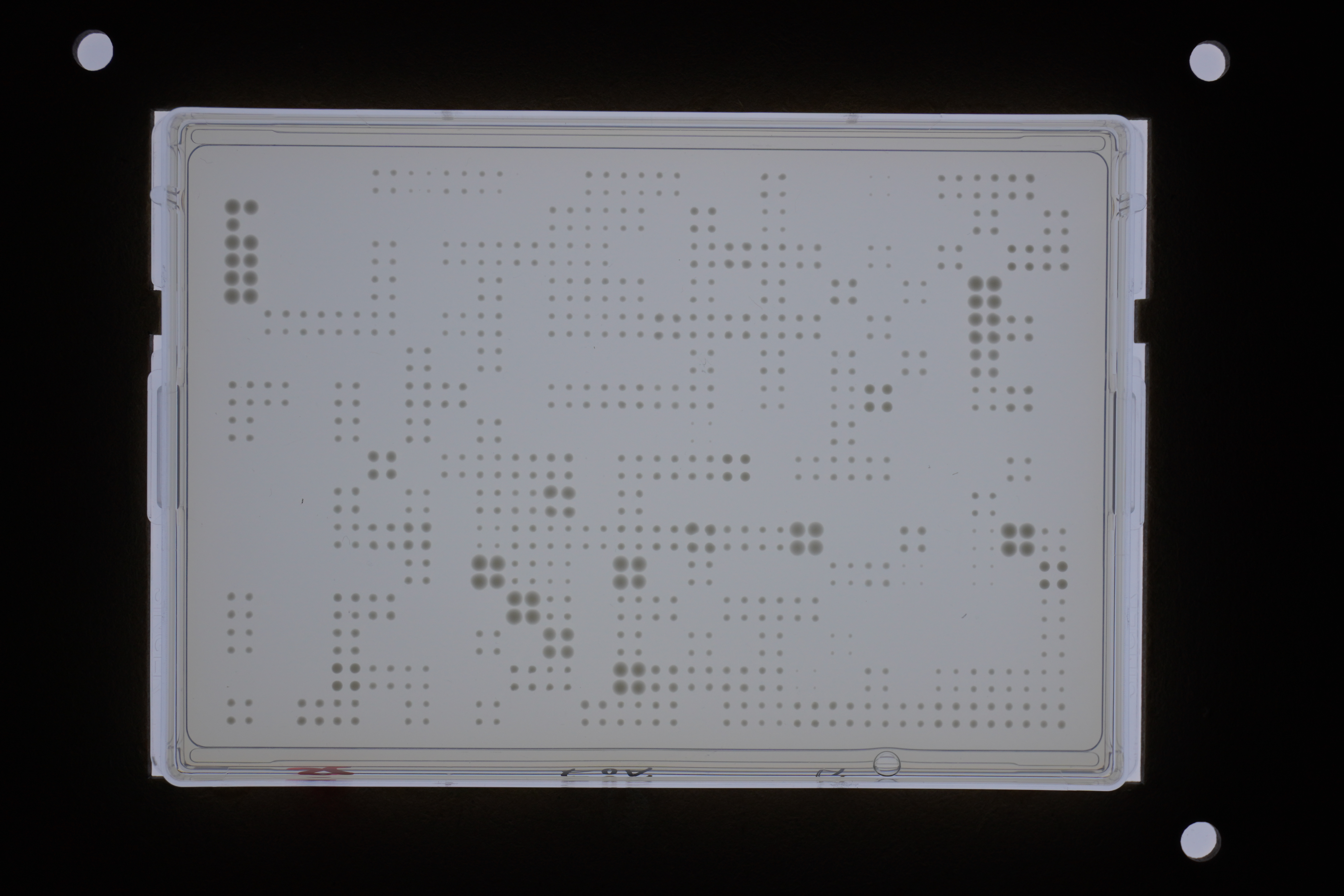

Supplement: Supplementary file 14 — Source data Fig. 2 [file 44319_2026_702_MOESM14_ESM.zip › Figure2B_SourceData/Images/SC_5FOA_6.TIFF]

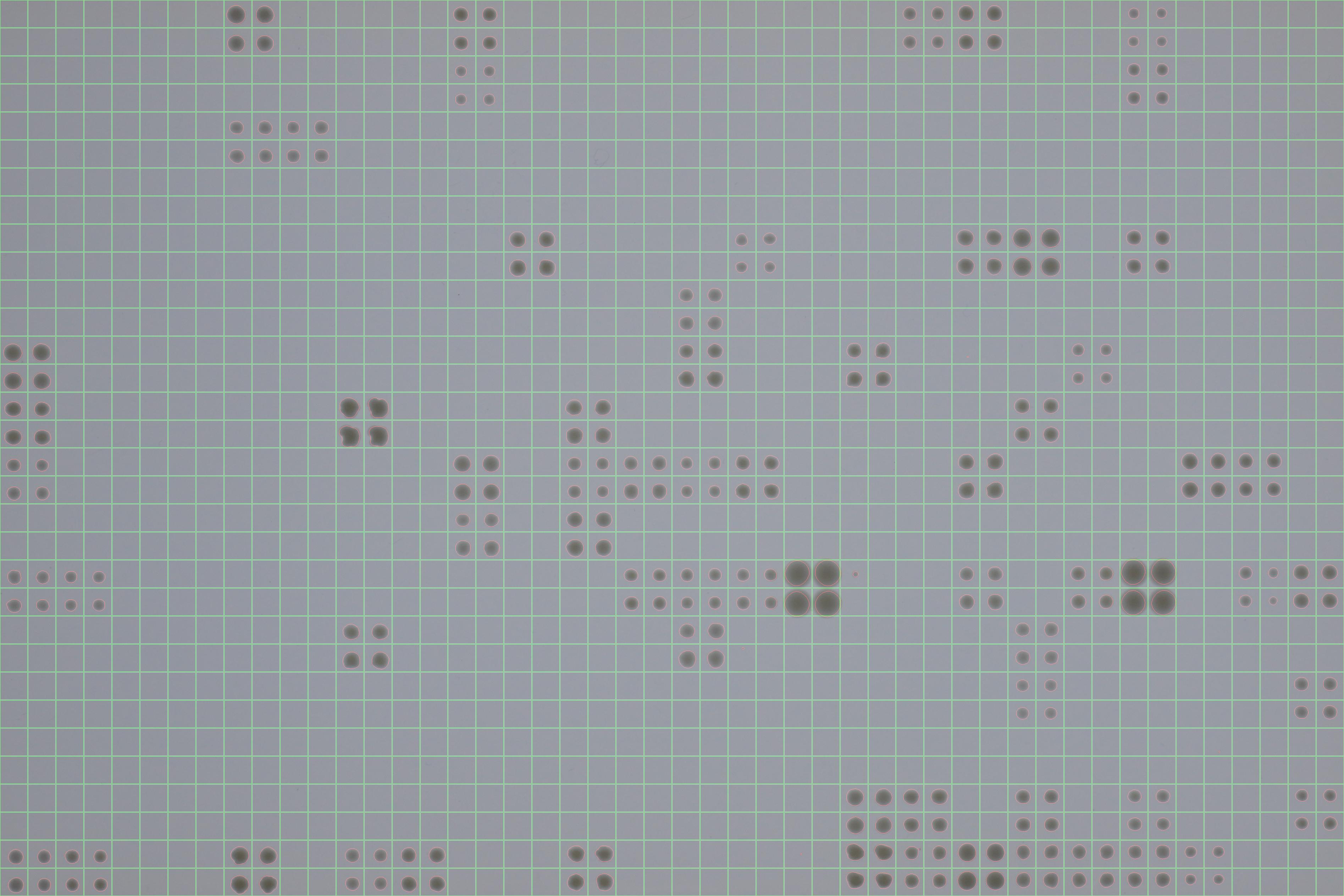

Supplement: Supplementary file 14 — Source data Fig. 2 [file 44319_2026_702_MOESM14_ESM.zip › Figure2B_SourceData/Images/SC_5FOA_segmented_1.TIFF]

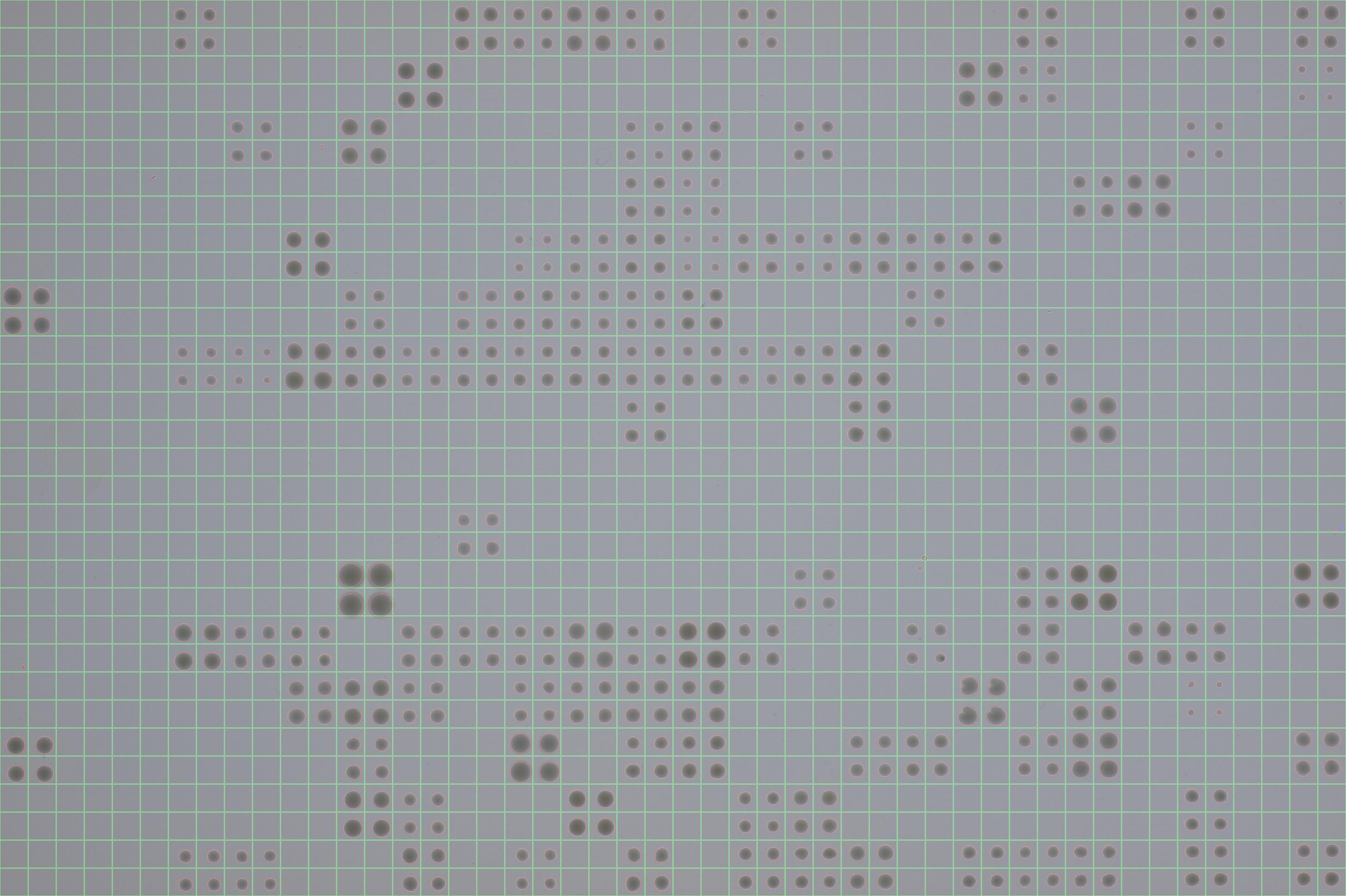

Supplement: Supplementary file 14 — Source data Fig. 2 [file 44319_2026_702_MOESM14_ESM.zip › Figure2B_SourceData/Images/SC_5FOA_segmented_2.TIFF]

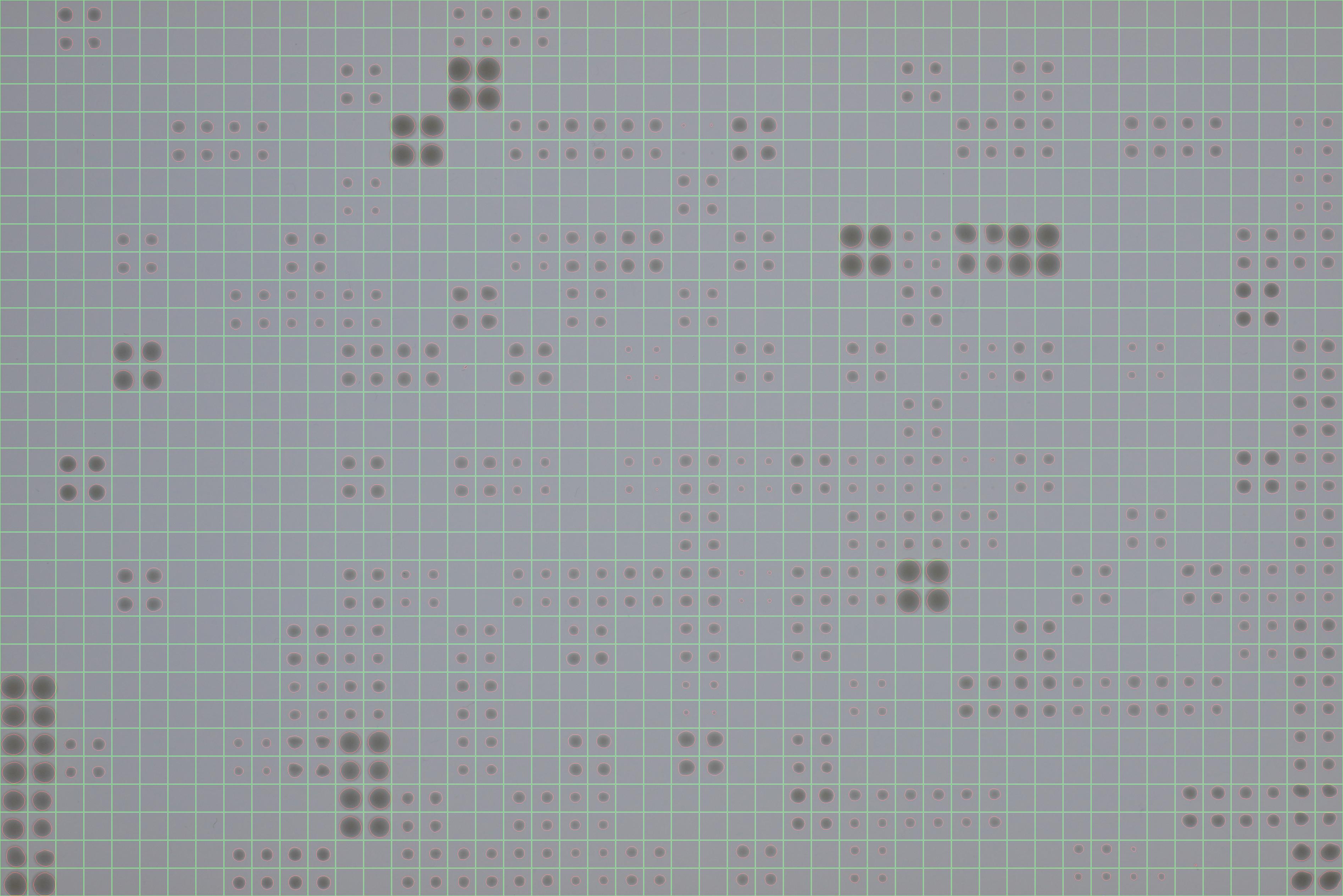

Supplement: Supplementary file 14 — Source data Fig. 2 [file 44319_2026_702_MOESM14_ESM.zip › Figure2B_SourceData/Images/SC_5FOA_segmented_3.TIFF]

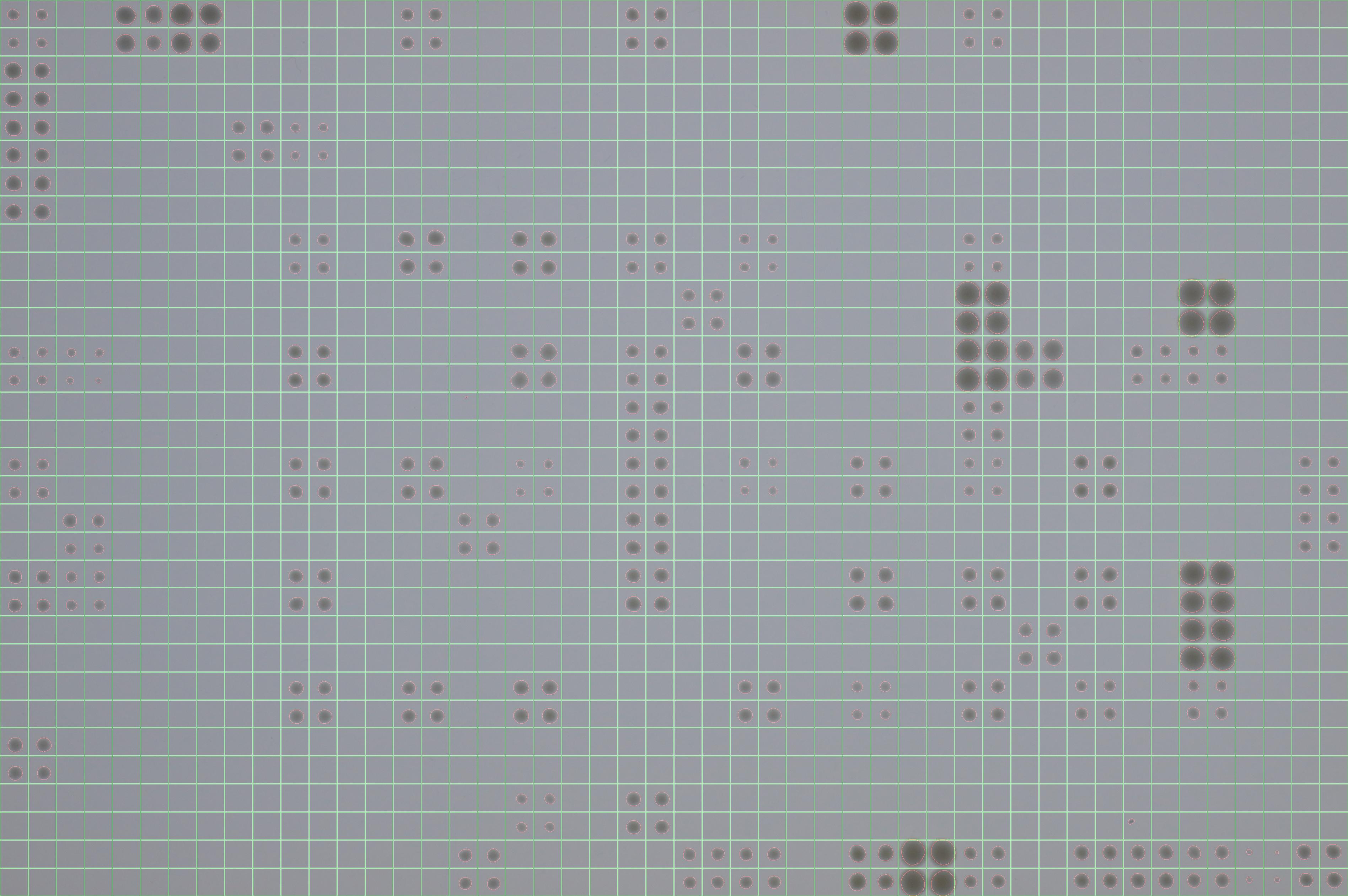

Supplement: Supplementary file 14 — Source data Fig. 2 [file 44319_2026_702_MOESM14_ESM.zip › Figure2B_SourceData/Images/SC_5FOA_segmented_4.TIFF]

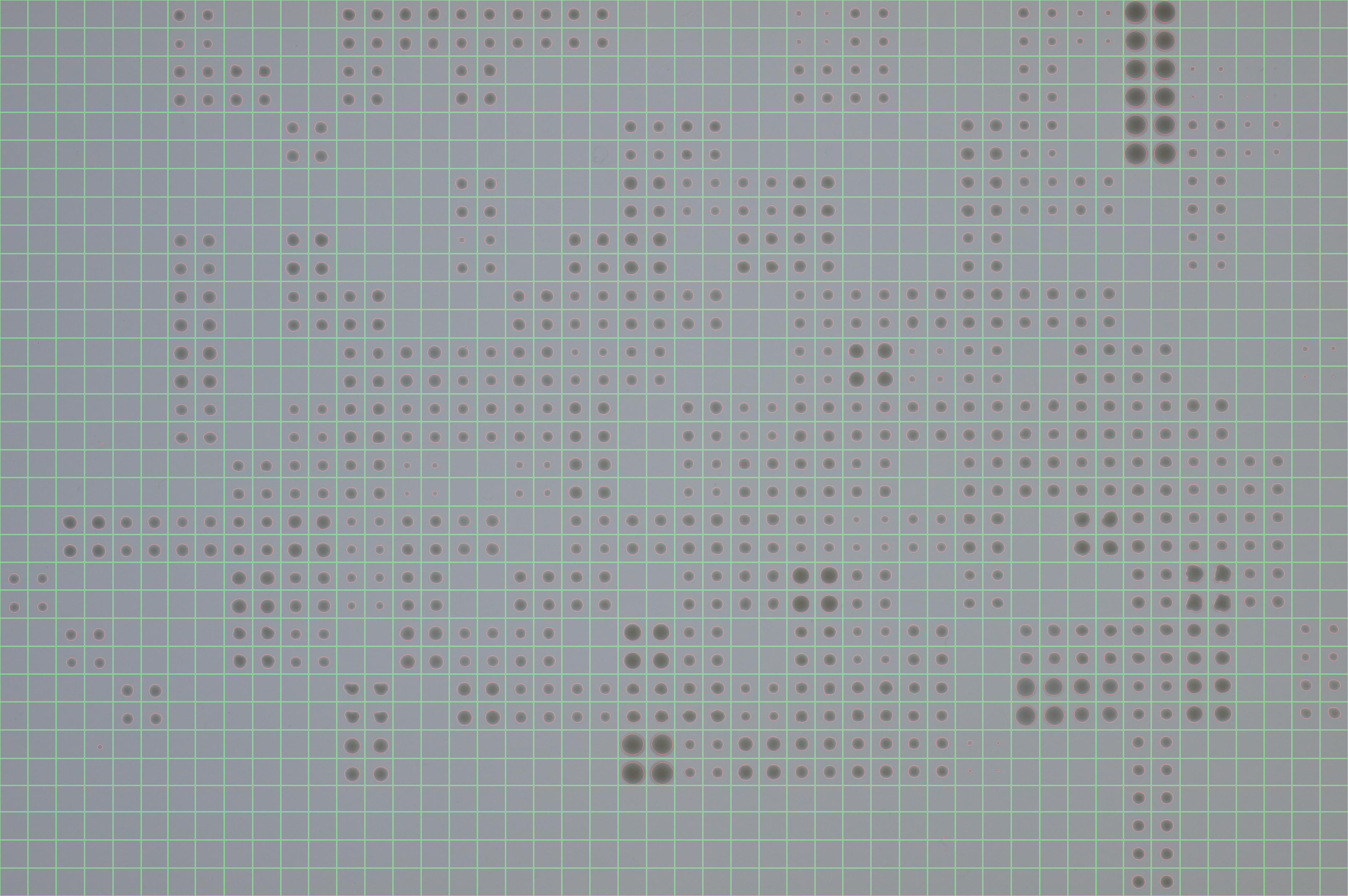

Supplement: Supplementary file 14 — Source data Fig. 2 [file 44319_2026_702_MOESM14_ESM.zip › Figure2B_SourceData/Images/SC_5FOA_segmented_5.TIFF]

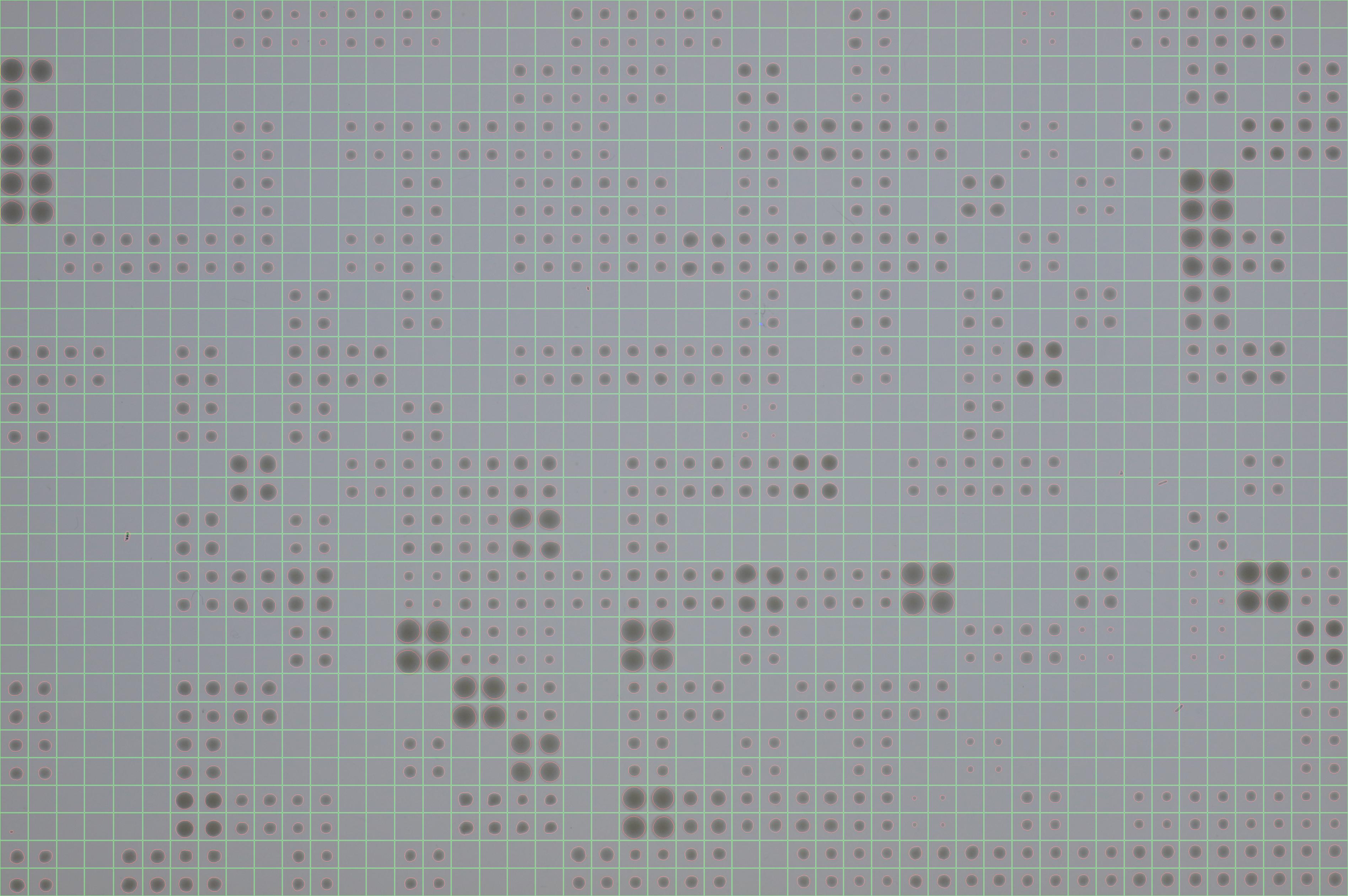

Supplement: Supplementary file 14 — Source data Fig. 2 [file 44319_2026_702_MOESM14_ESM.zip › Figure2B_SourceData/Images/SC_5FOA_segmented_6.TIFF]

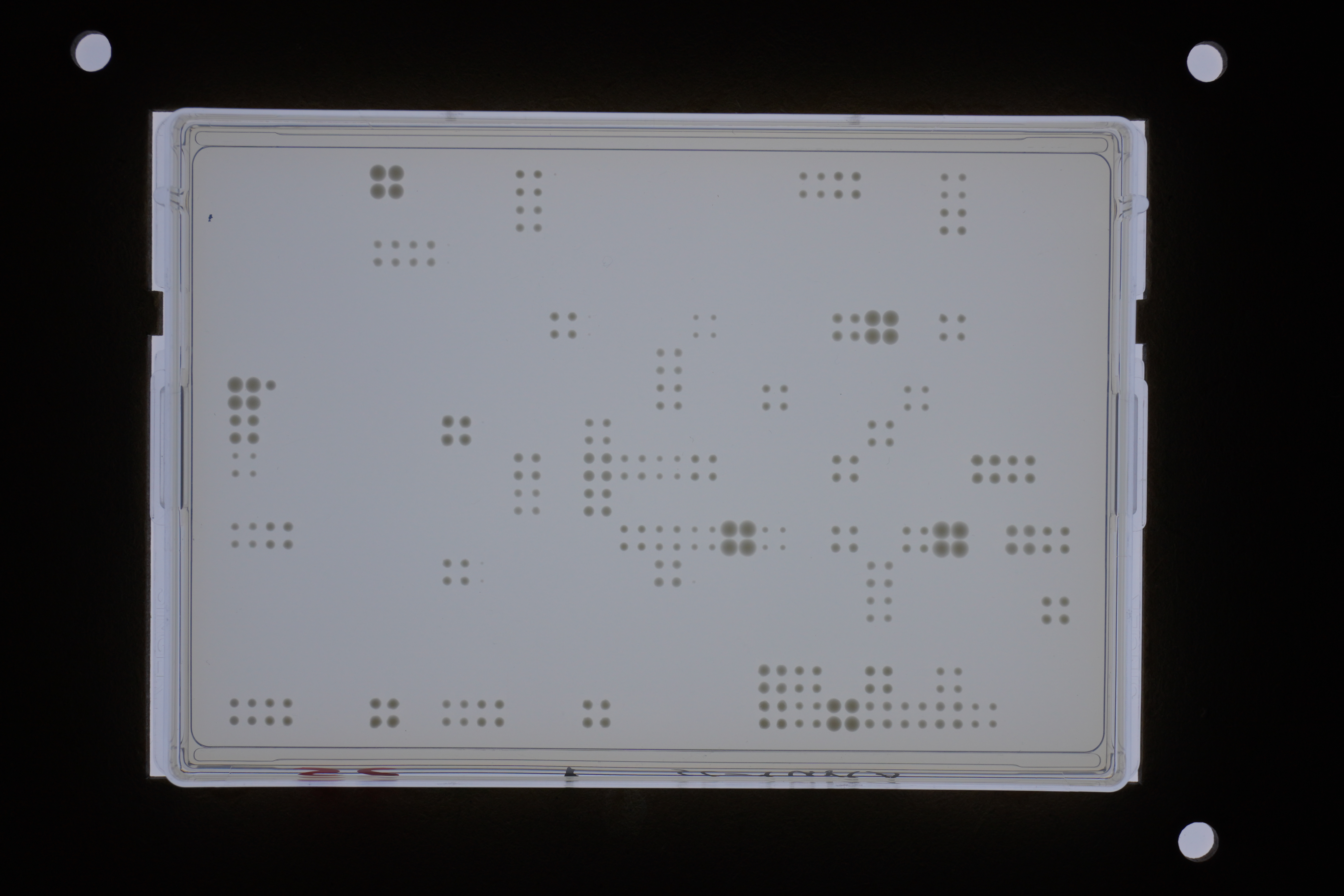

Supplement: Supplementary file 14 — Source data Fig. 2 [file 44319_2026_702_MOESM14_ESM.zip › Figure2B_SourceData/Images/SC_SCminURA_1.TIFF]

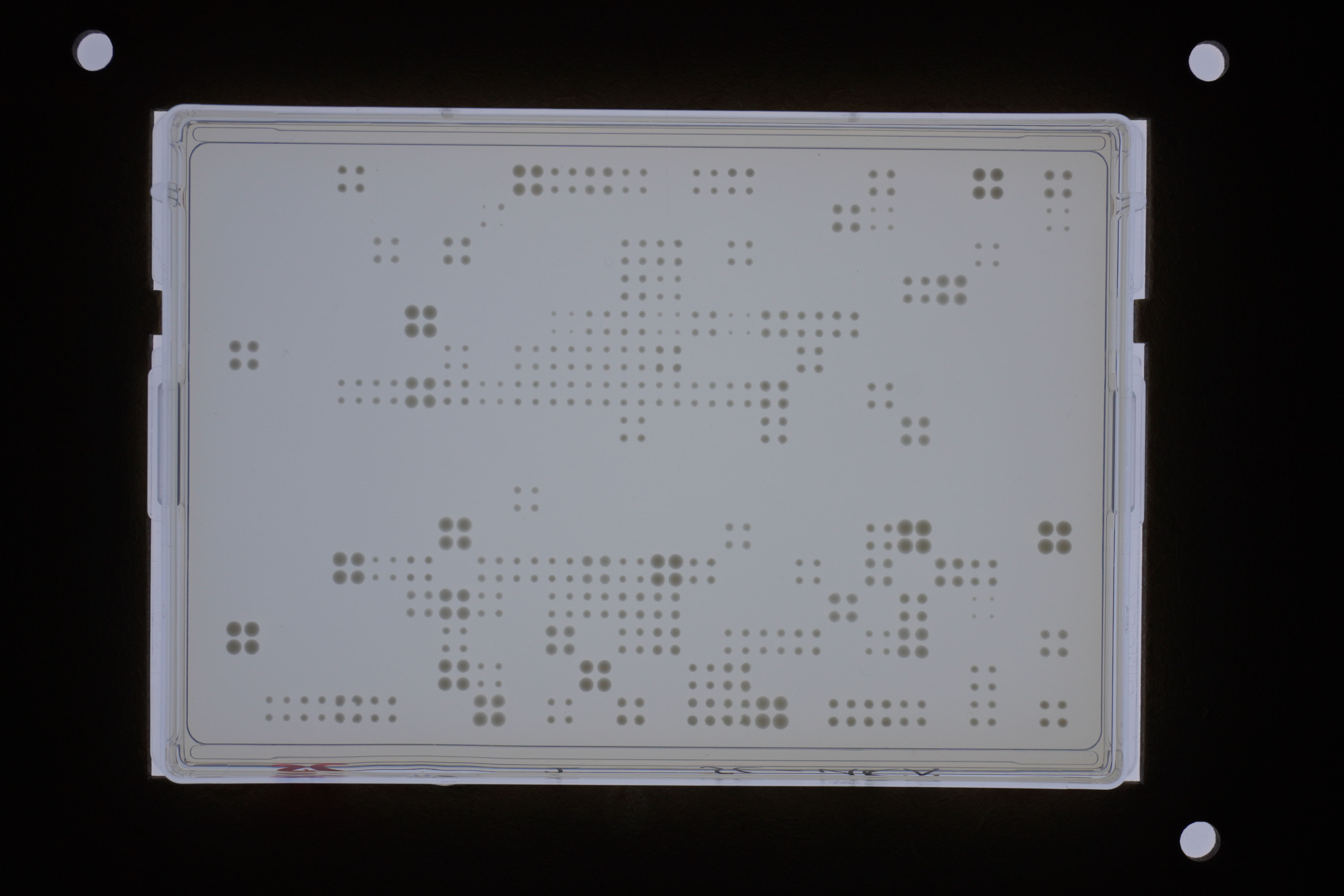

Supplement: Supplementary file 14 — Source data Fig. 2 [file 44319_2026_702_MOESM14_ESM.zip › Figure2B_SourceData/Images/SC_SCminURA_2.TIFF]

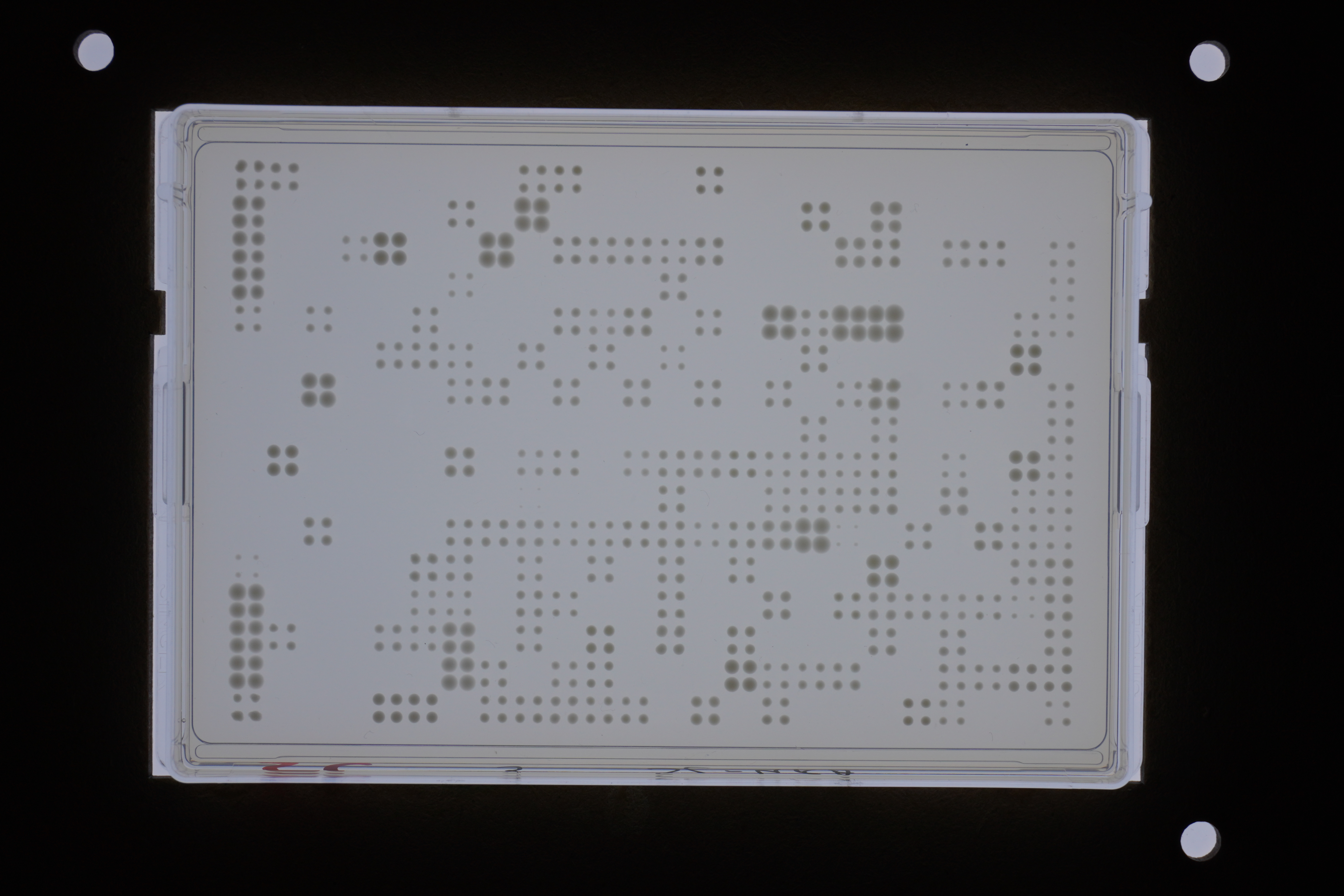

Supplement: Supplementary file 14 — Source data Fig. 2 [file 44319_2026_702_MOESM14_ESM.zip › Figure2B_SourceData/Images/SC_SCminURA_3.TIFF]

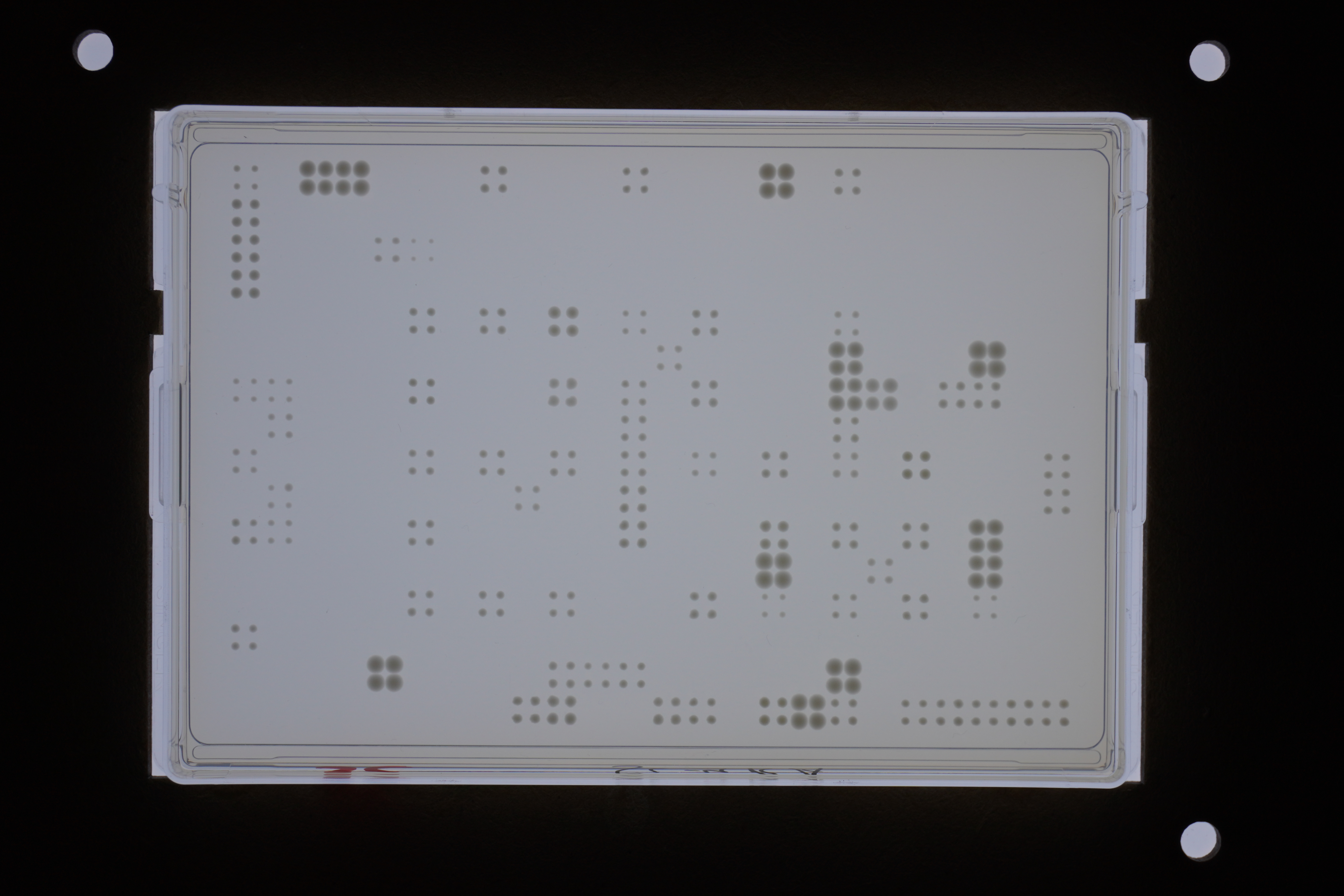

Supplement: Supplementary file 14 — Source data Fig. 2 [file 44319_2026_702_MOESM14_ESM.zip › Figure2B_SourceData/Images/SC_SCminURA_4.TIFF]

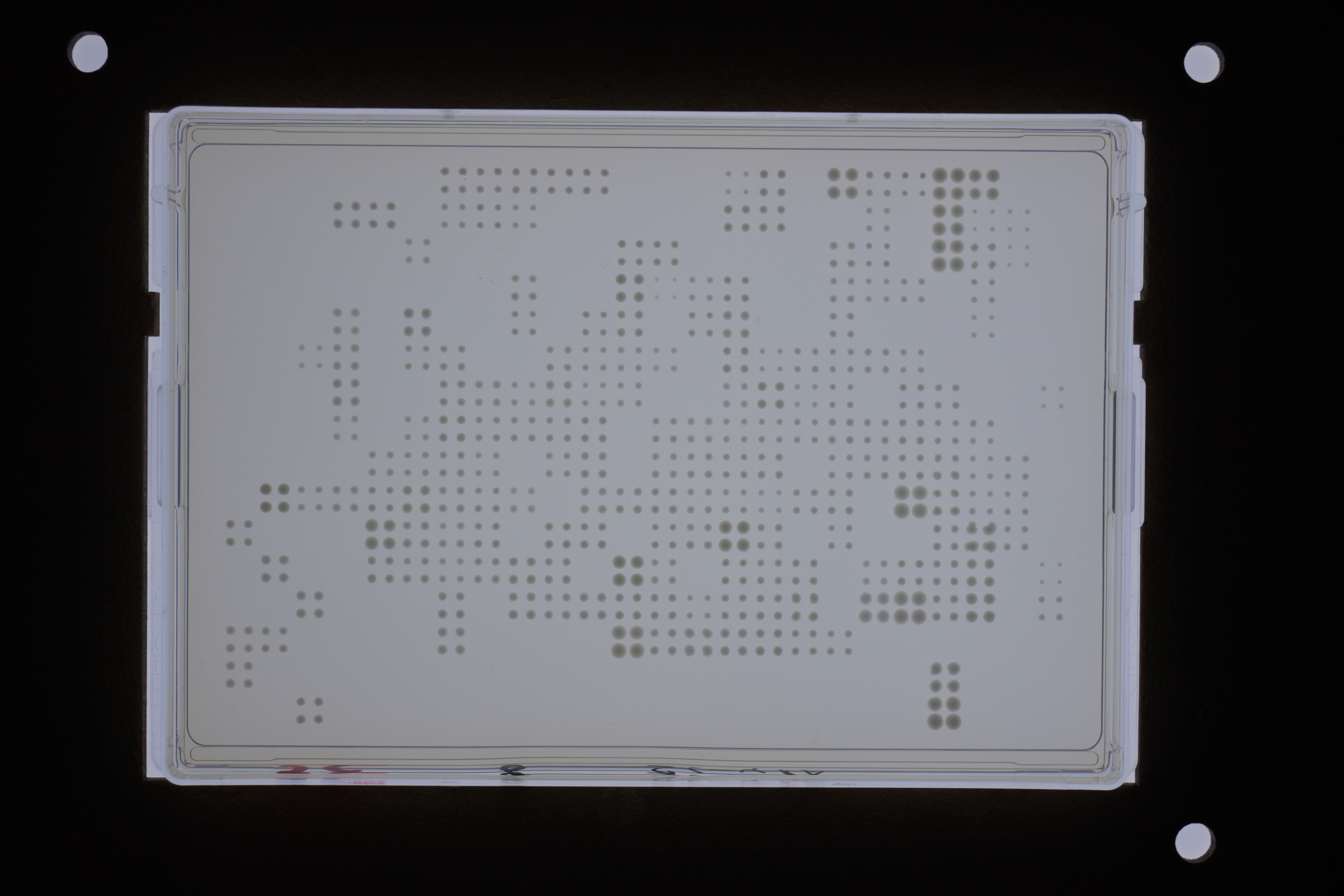

Supplement: Supplementary file 14 — Source data Fig. 2 [file 44319_2026_702_MOESM14_ESM.zip › Figure2B_SourceData/Images/SC_SCminURA_5.TIFF]

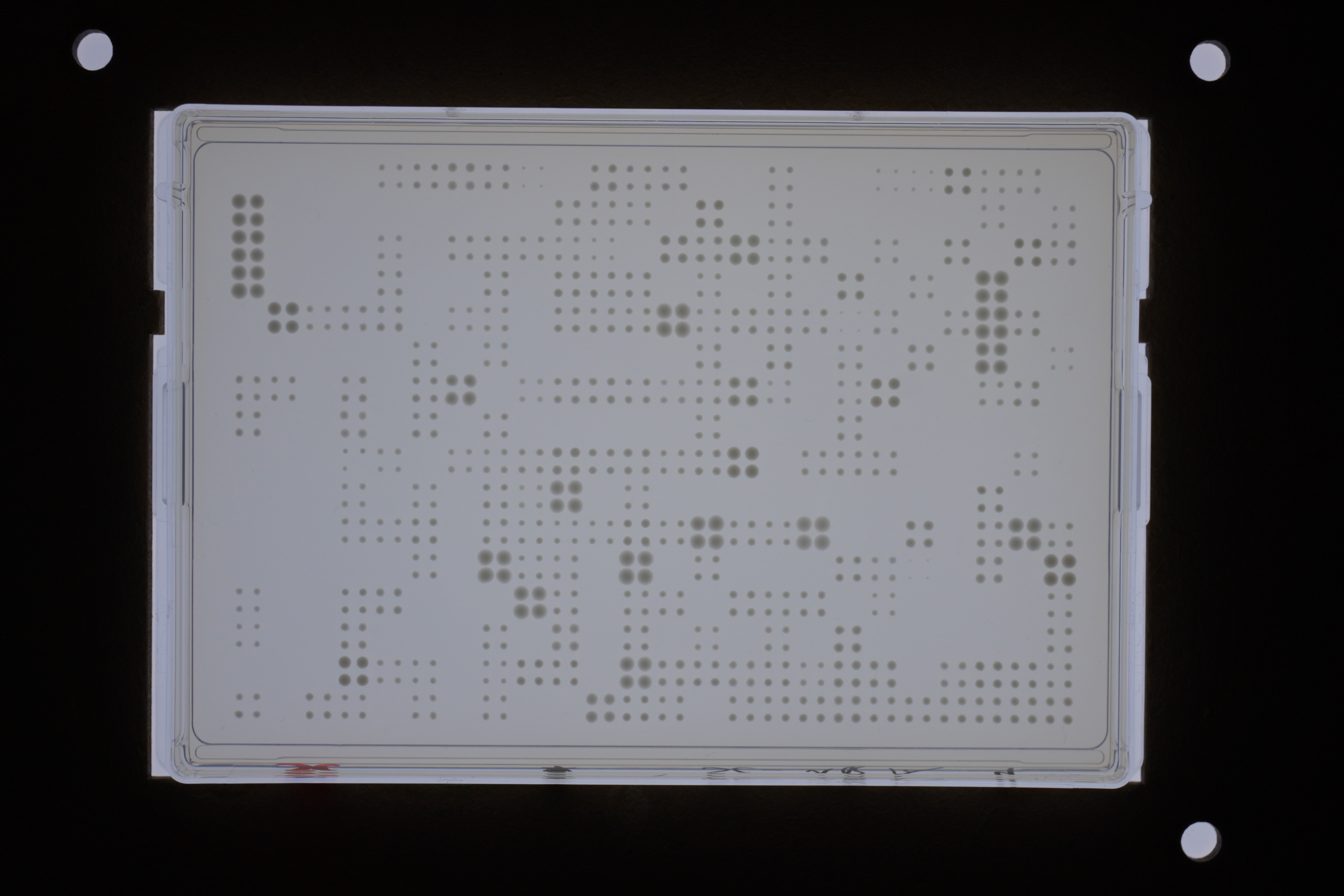

Supplement: Supplementary file 14 — Source data Fig. 2 [file 44319_2026_702_MOESM14_ESM.zip › Figure2B_SourceData/Images/SC_SCminURA_6.TIFF]

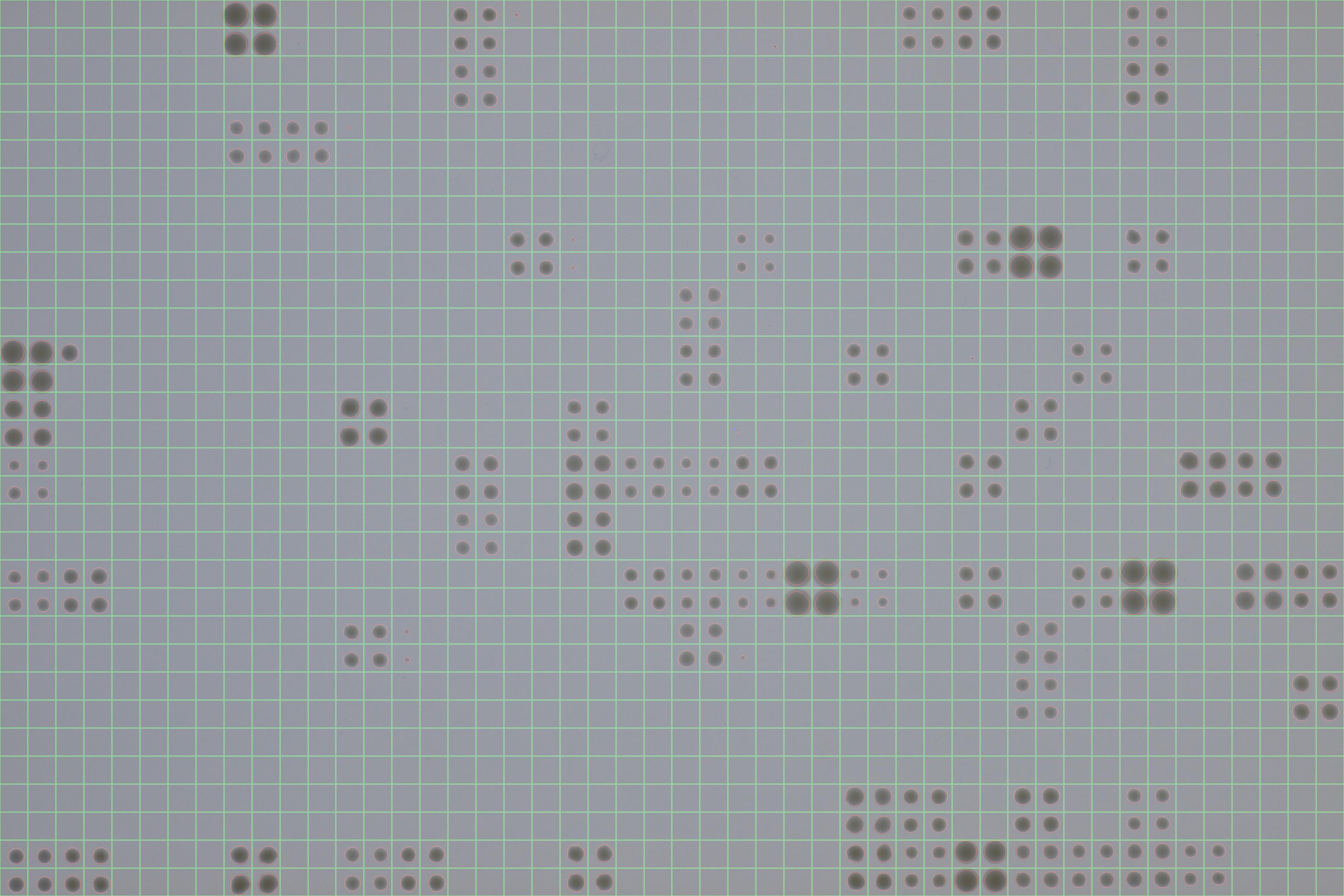

Supplement: Supplementary file 14 — Source data Fig. 2 [file 44319_2026_702_MOESM14_ESM.zip › Figure2B_SourceData/Images/SC_SCminURA_segmented_1.TIFF]

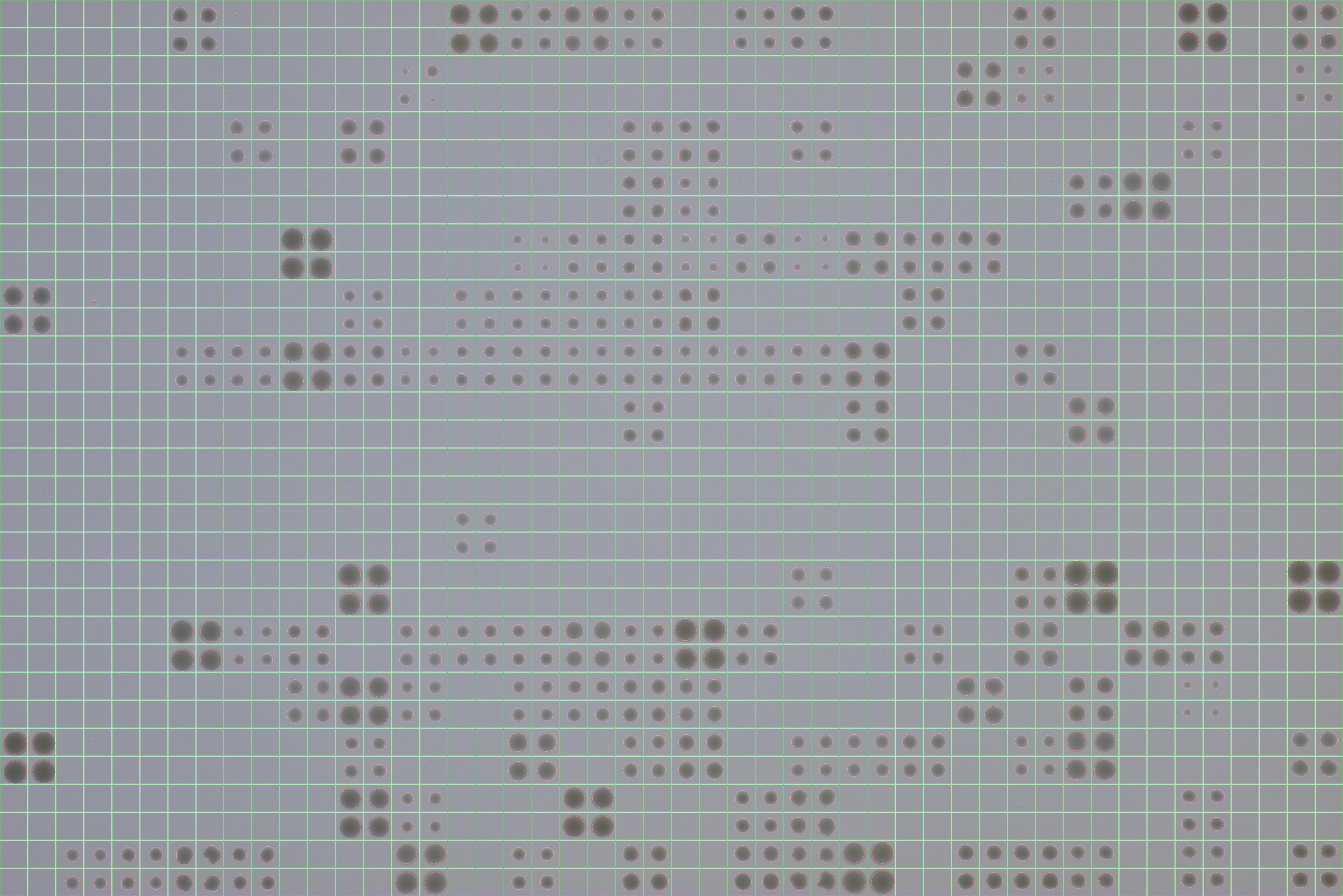

Supplement: Supplementary file 14 — Source data Fig. 2 [file 44319_2026_702_MOESM14_ESM.zip › Figure2B_SourceData/Images/SC_SCminURA_segmented_2.TIFF]

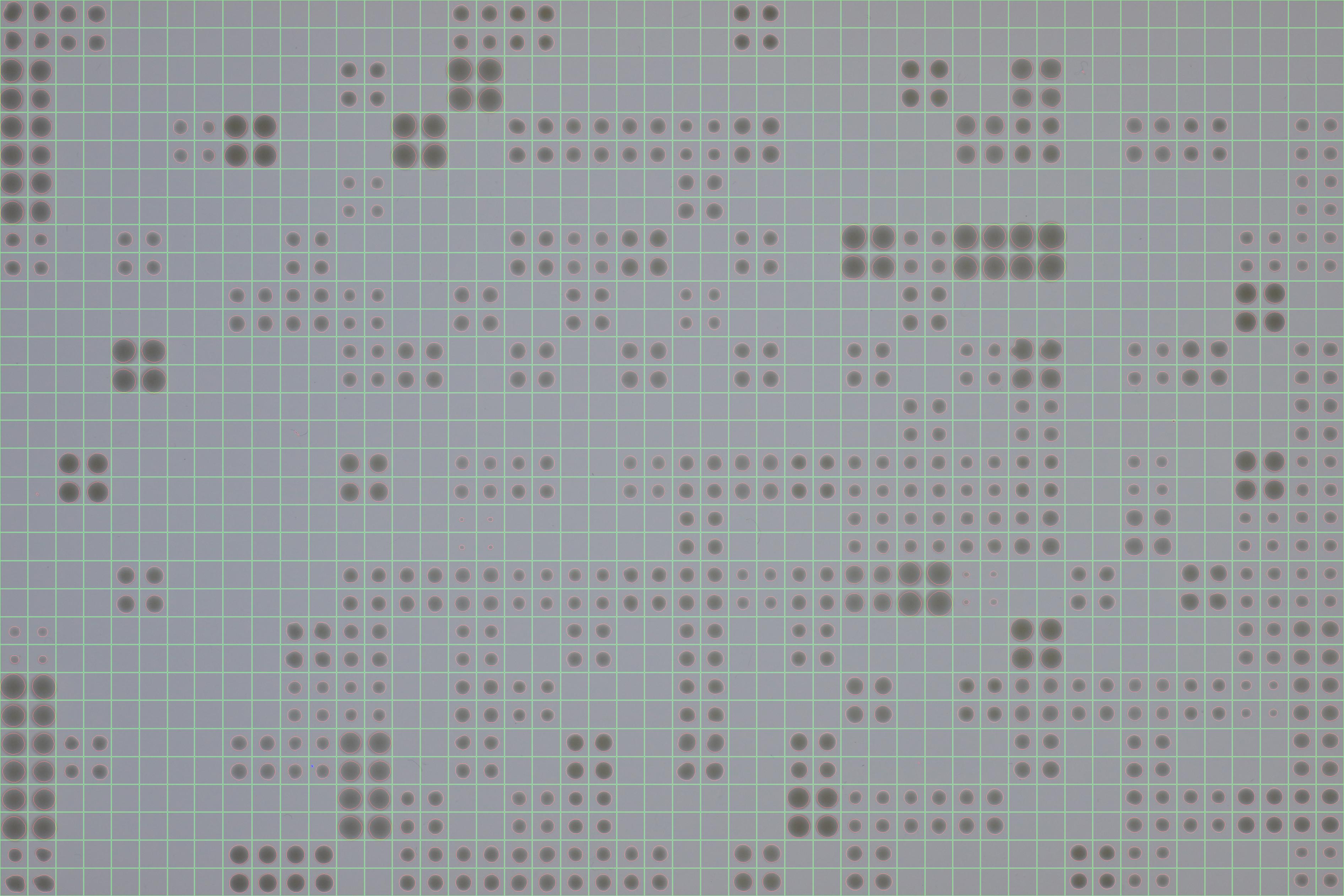

Supplement: Supplementary file 14 — Source data Fig. 2 [file 44319_2026_702_MOESM14_ESM.zip › Figure2B_SourceData/Images/SC_SCminURA_segmented_3.TIFF]

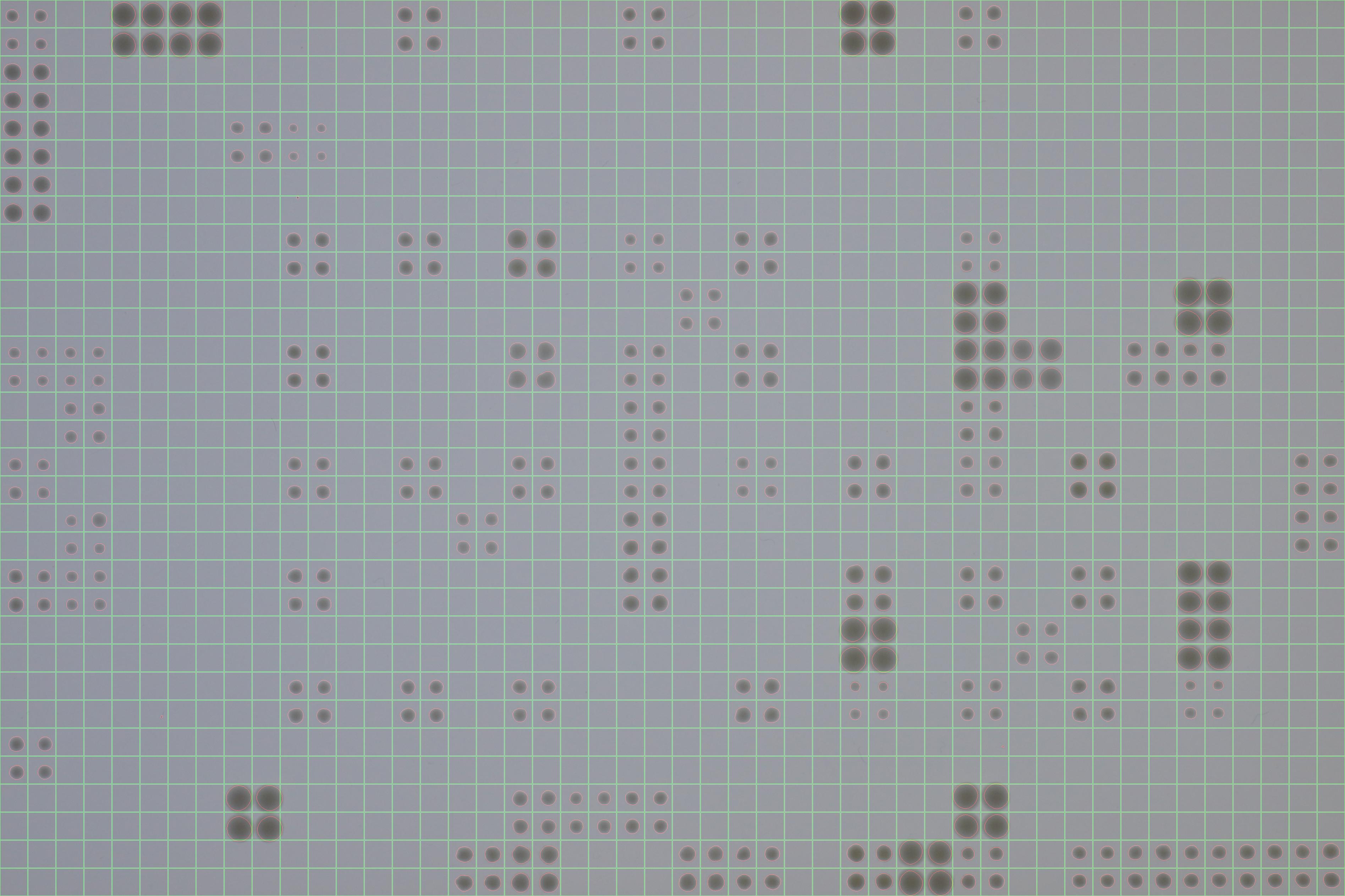

Supplement: Supplementary file 14 — Source data Fig. 2 [file 44319_2026_702_MOESM14_ESM.zip › Figure2B_SourceData/Images/SC_SCminURA_segmented_4.TIFF]

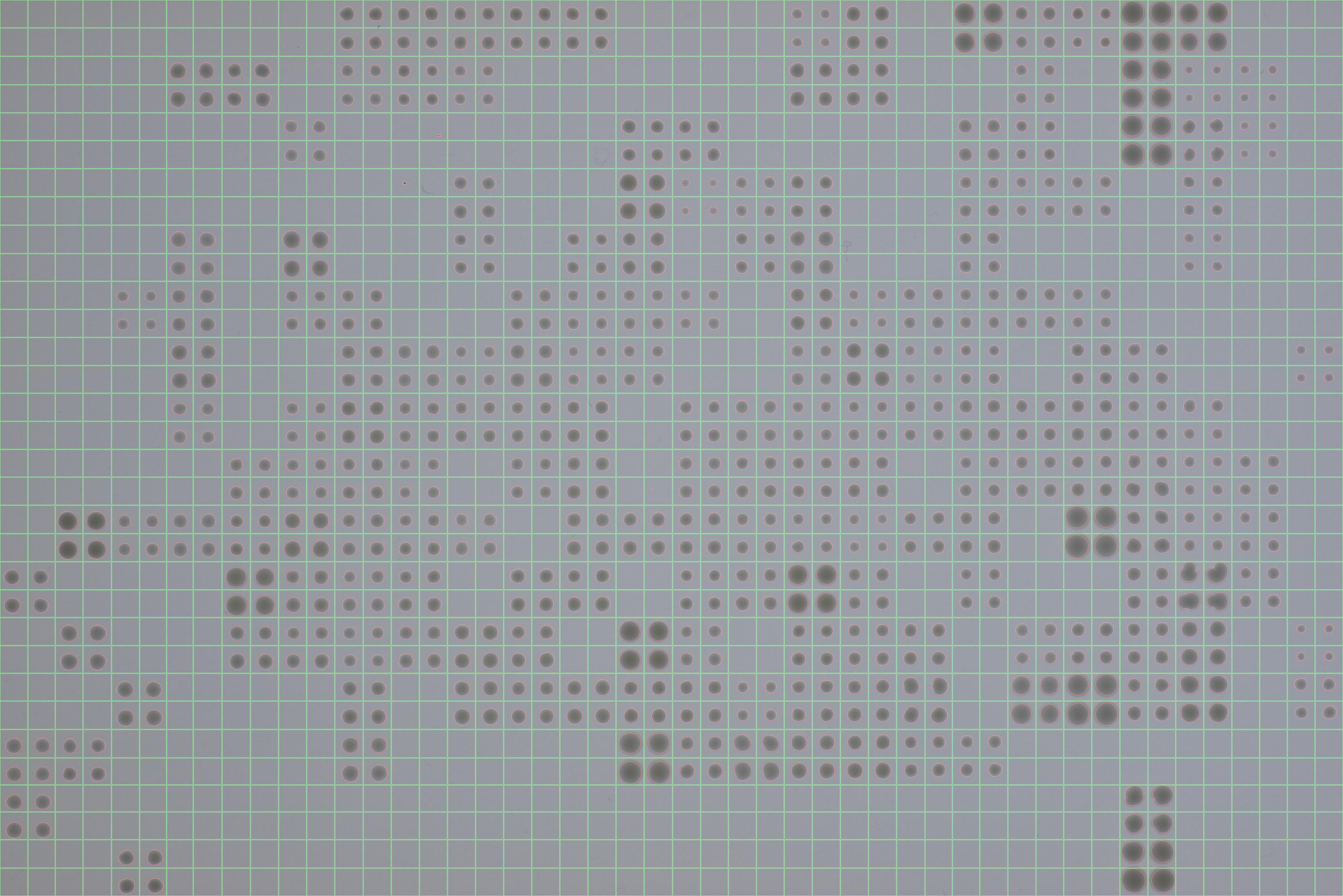

Supplement: Supplementary file 14 — Source data Fig. 2 [file 44319_2026_702_MOESM14_ESM.zip › Figure2B_SourceData/Images/SC_SCminURA_segmented_5.TIFF]

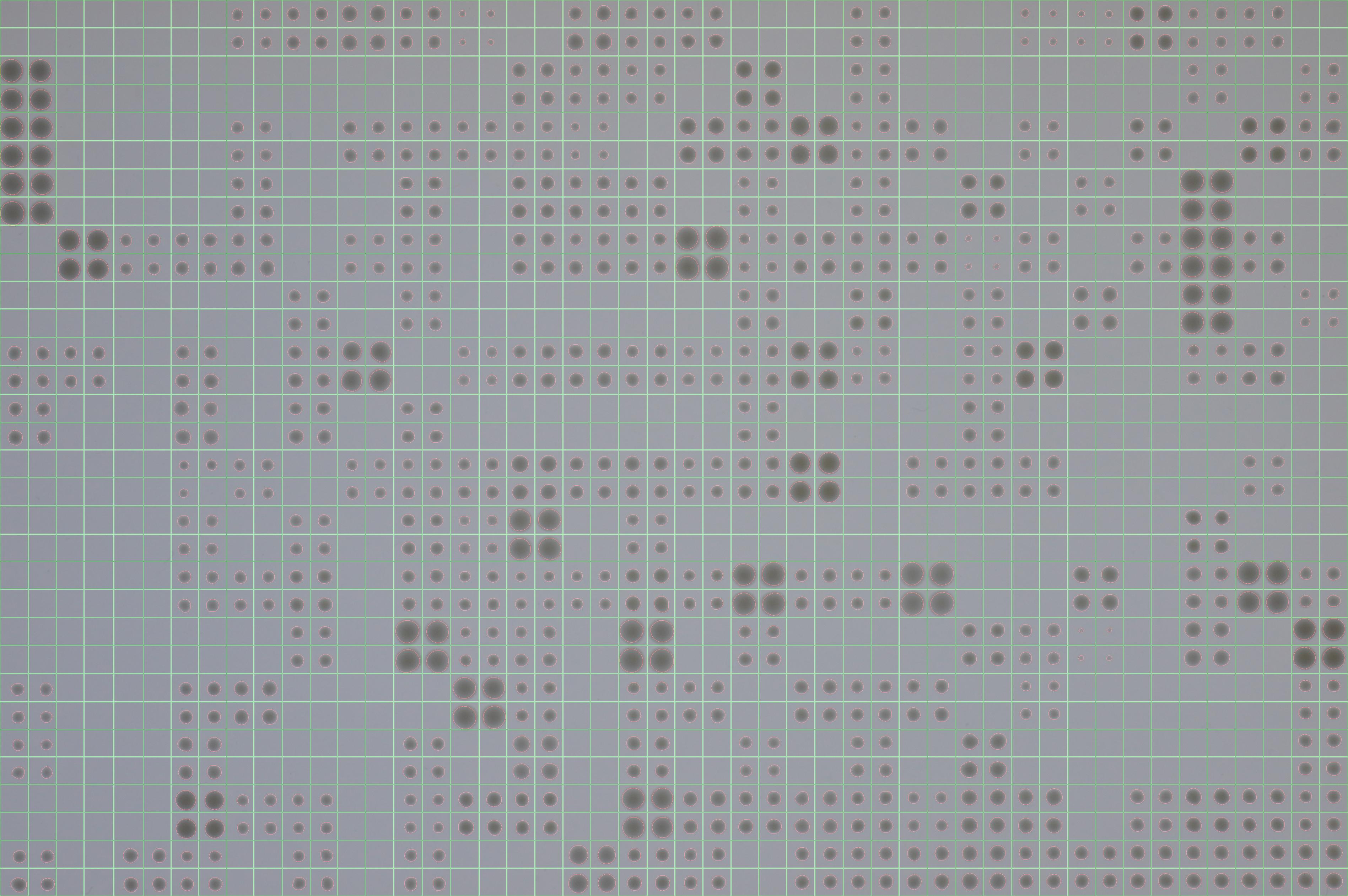

Supplement: Supplementary file 14 — Source data Fig. 2 [file 44319_2026_702_MOESM14_ESM.zip › Figure2B_SourceData/Images/SC_SCminURA_segmented_6.TIFF]

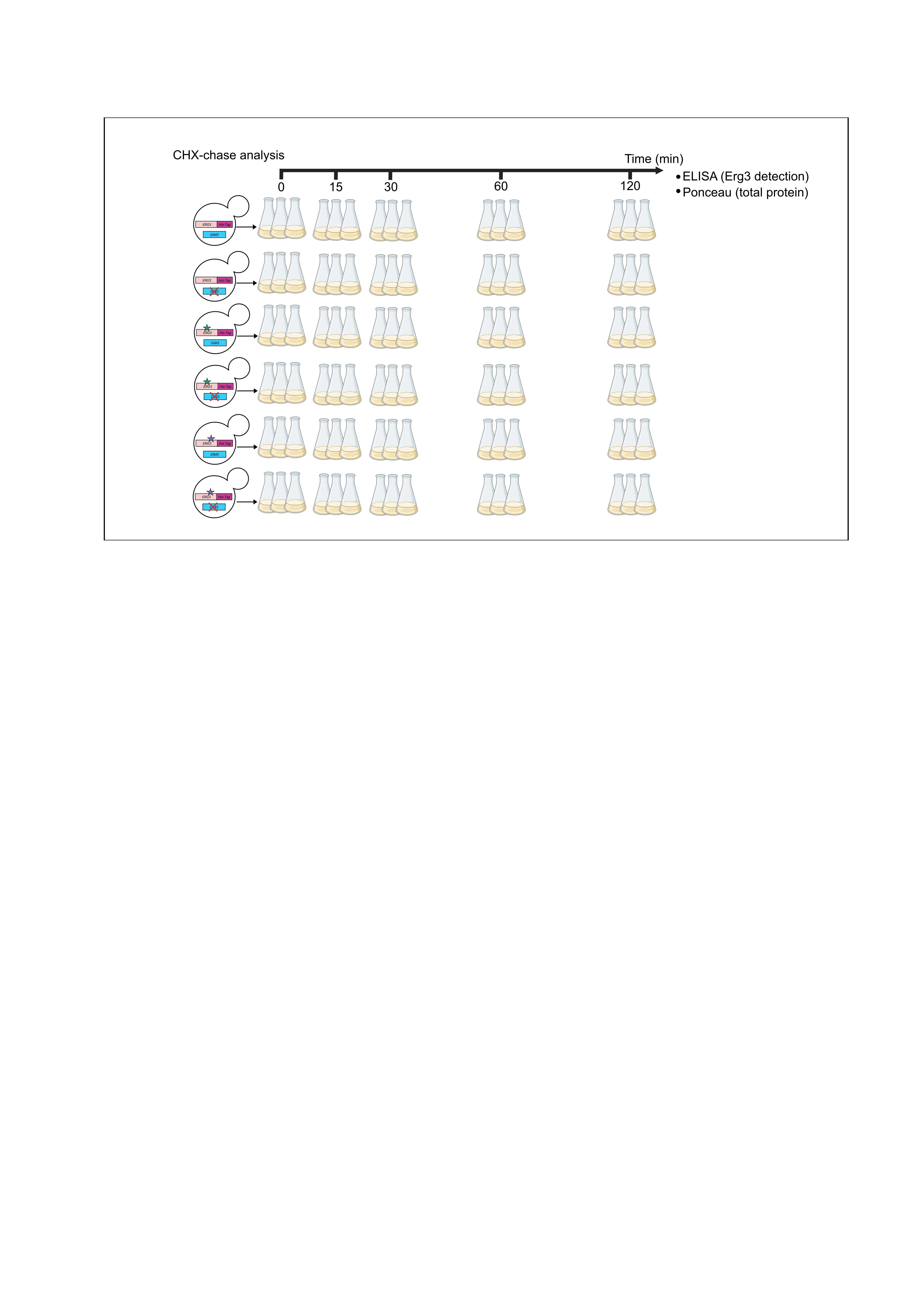

Supplement: Supplementary file 16 — Source data Fig. 4 [file 44319_2026_702_MOESM16_ESM.zip › Figure 4_SourceData/4A/Cycloheximide chase assay_experimental setup.png]

WT

WT + G584A

WT + A865C

WT + *erg3del*

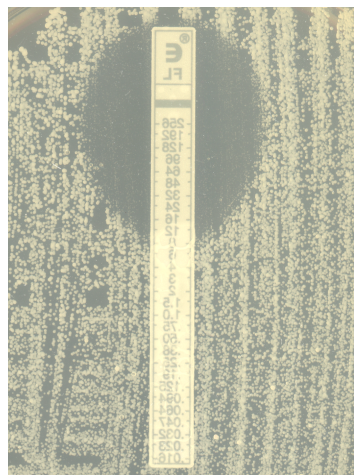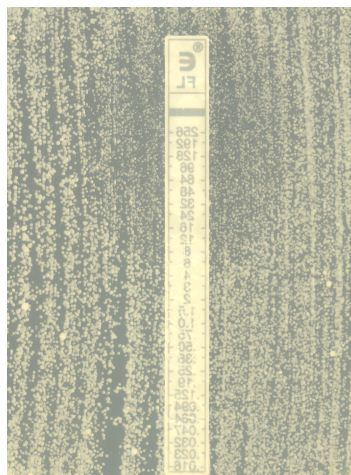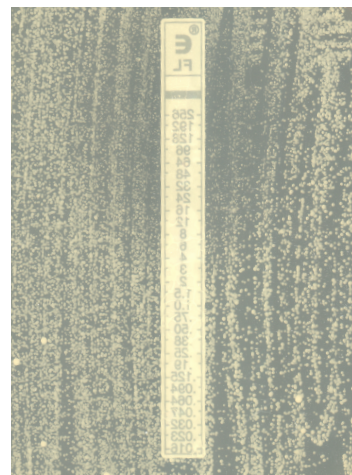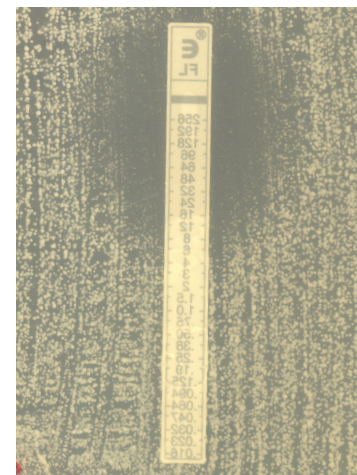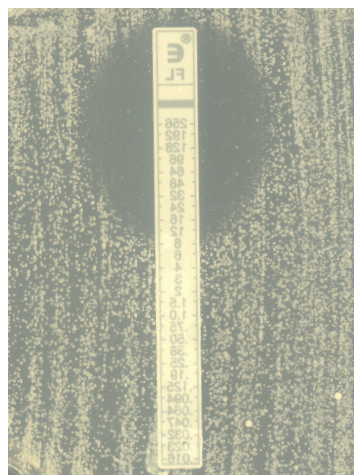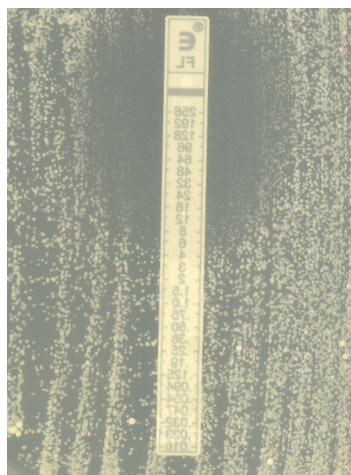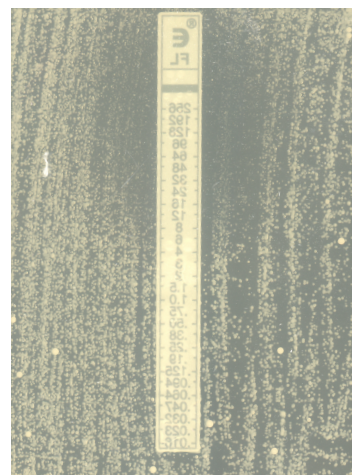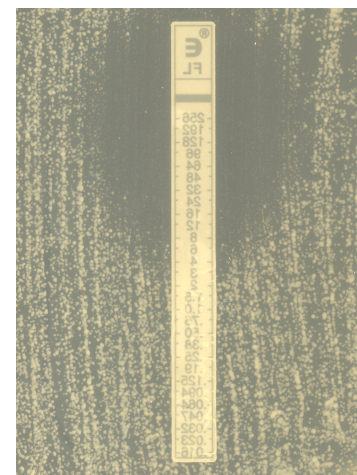

*gim3 del* + wt  
*erg3*

*gim3del* +  
G584A

*gim3del* +  
A865C

*gim3del* +  
*erg3del*

Supplement: Supplementary file 16 — Source data Fig. 4 [file 44319_2026_702_MOESM16_ESM.zip › Figure 4_SourceData/4C/Images/24Hours.pdf]

WT

WT + G584A

WT + A865C

WT + erg3del

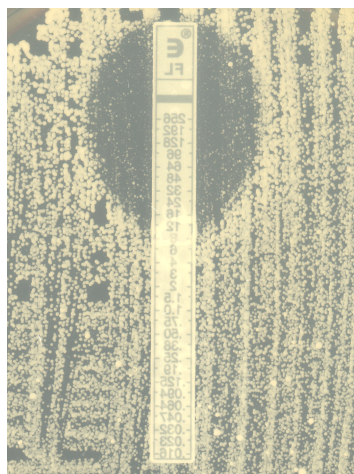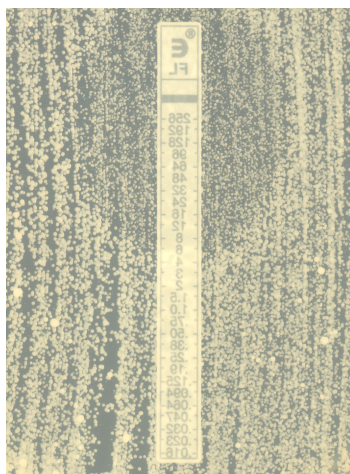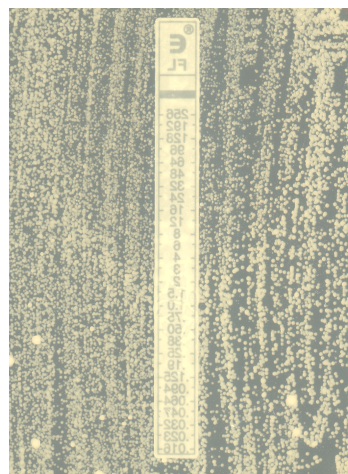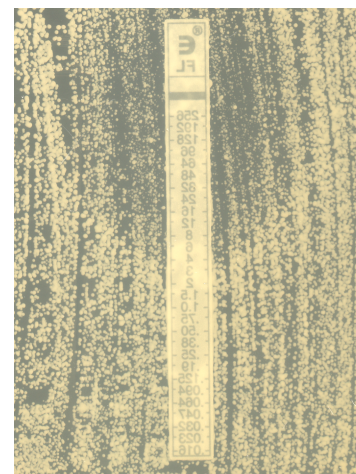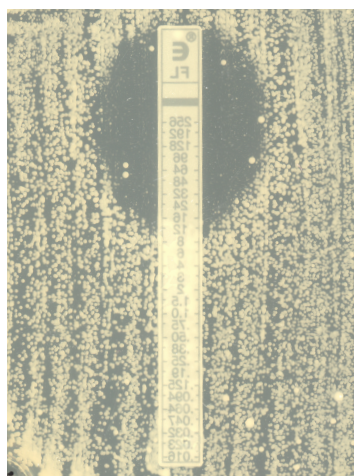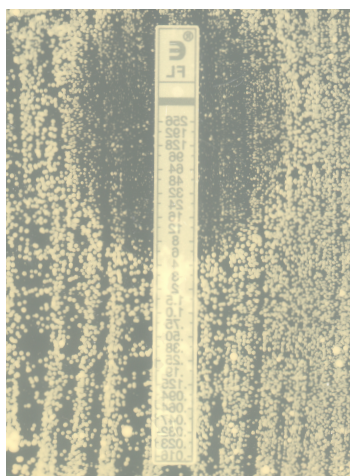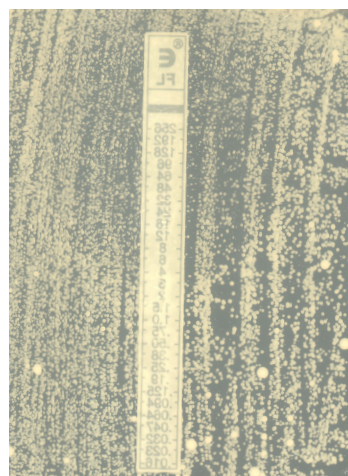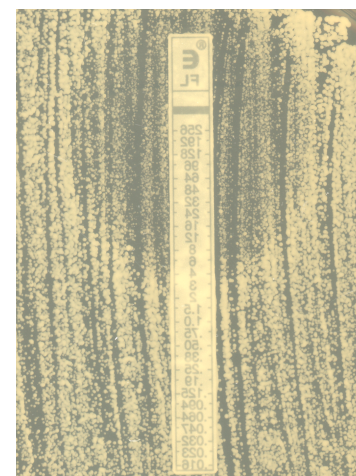

gim3 del + wt  
erg3

gim3del +  
G584A

gim3del +  
A865C

gim3del +  
erg3del

Supplement: Supplementary file 16 — Source data Fig. 4 [file 44319_2026_702_MOESM16_ESM.zip › Figure 4_SourceData/4C/Images/48Hours.pdf]

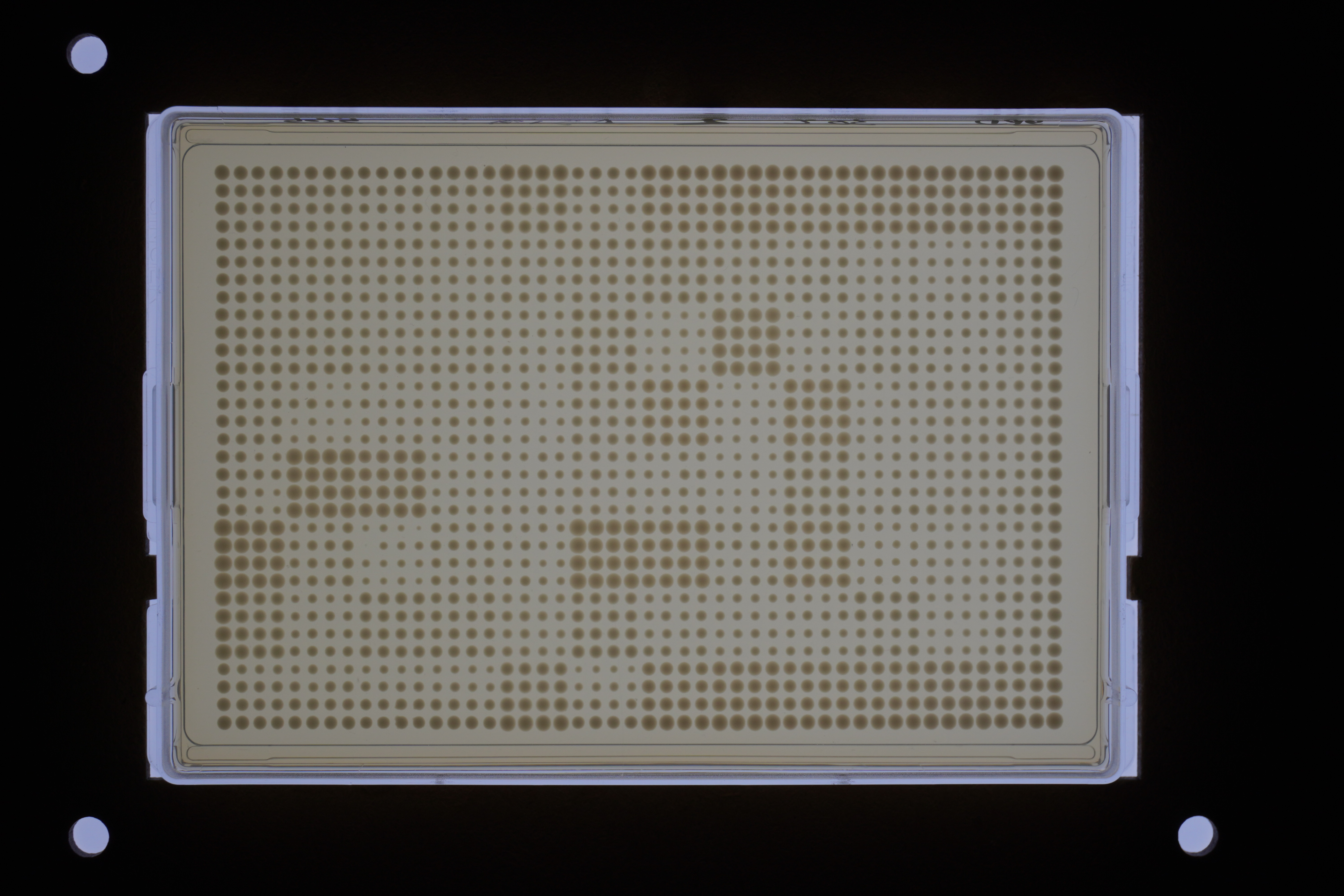

Supplement: Supplementary file 17 — Source data Fig. 5 [file 44319_2026_702_MOESM17_ESM.zip › Figure 5_SourceData/5C_to_5F/Images/30C_KO_48h.TIFF]

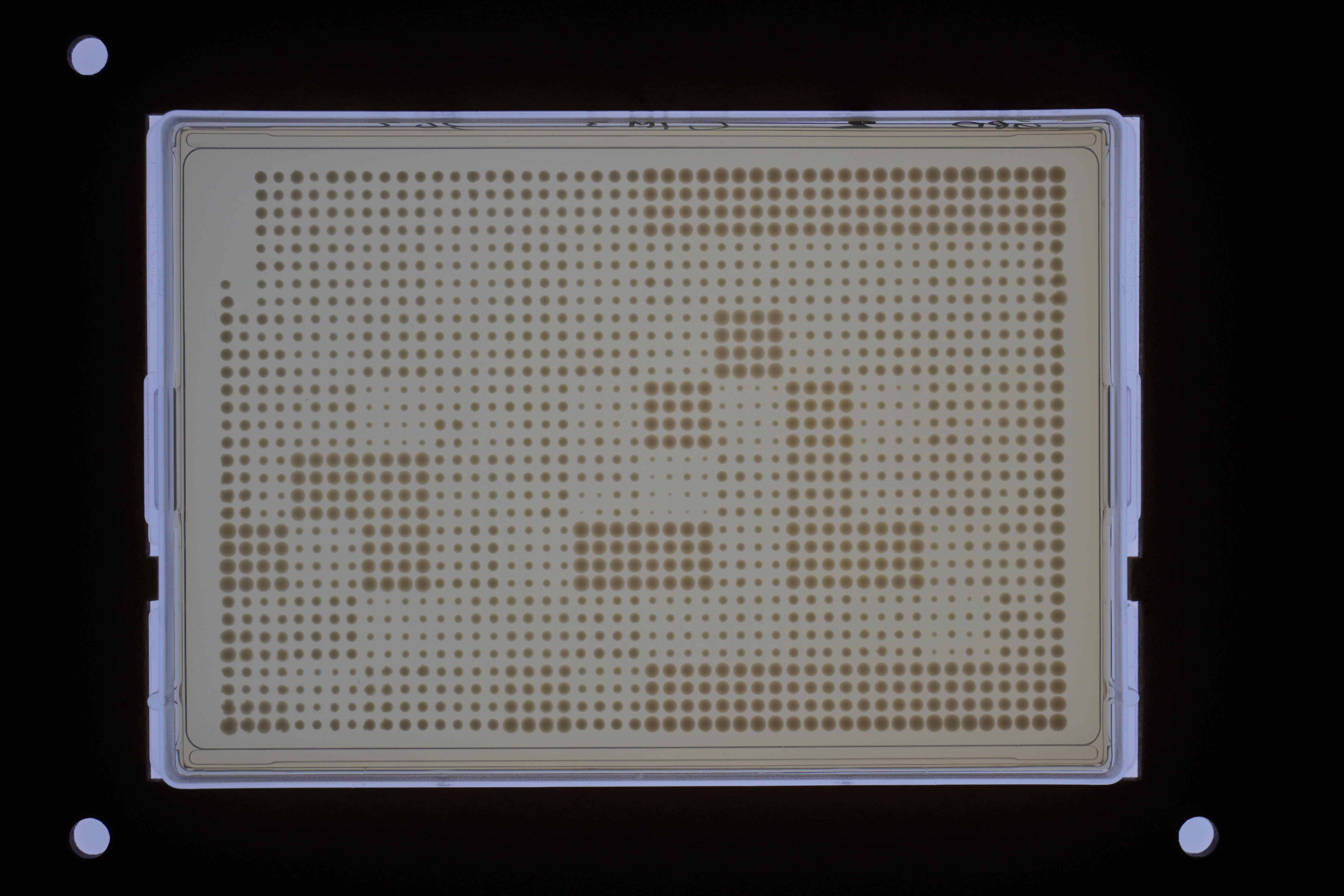

Supplement: Supplementary file 17 — Source data Fig. 5 [file 44319_2026_702_MOESM17_ESM.zip › Figure 5_SourceData/5C_to_5F/Images/30C_WT_48h.TIFF]

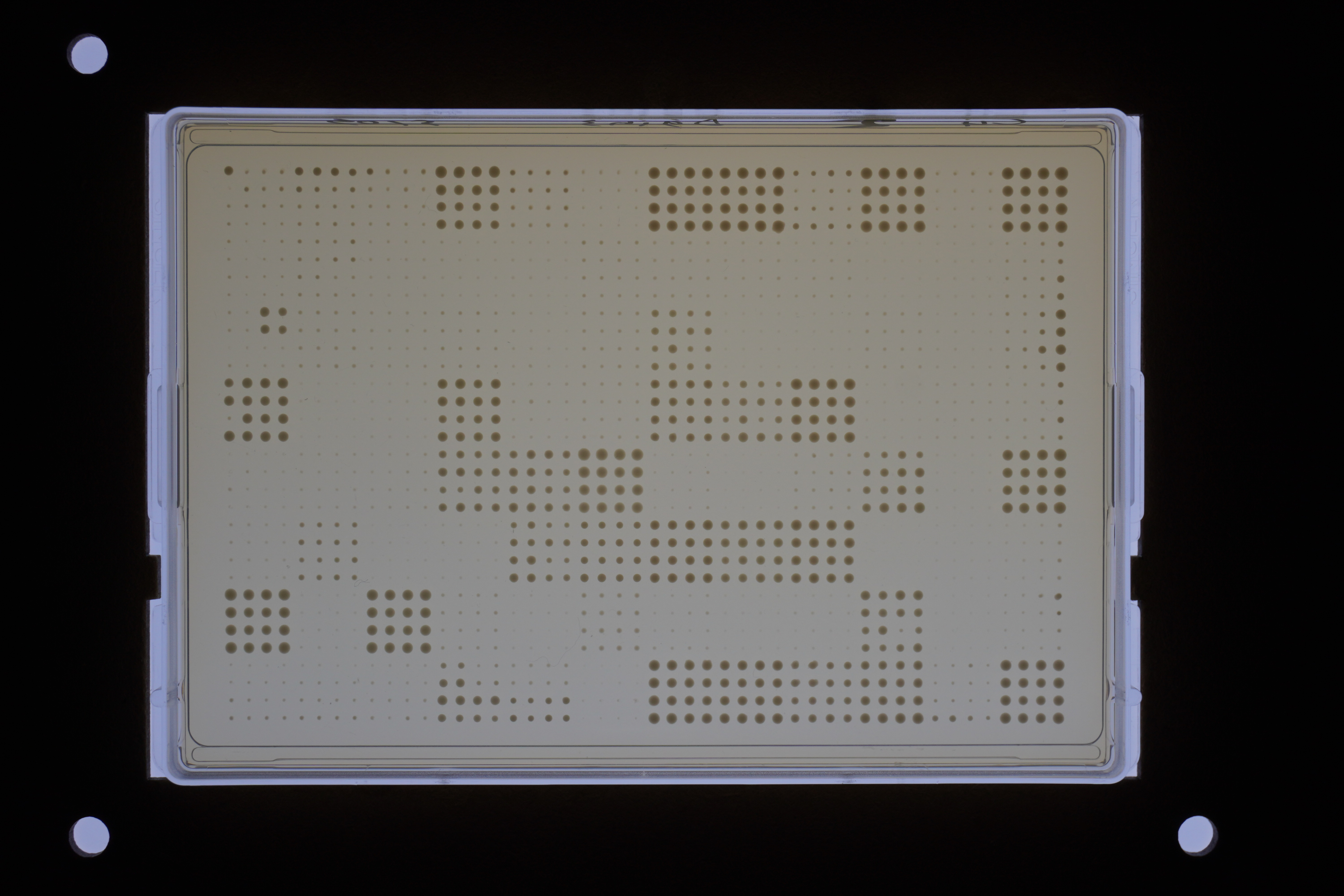

Supplement: Supplementary file 17 — Source data Fig. 5 [file 44319_2026_702_MOESM17_ESM.zip › Figure 5_SourceData/5C_to_5F/Images/Caspofungine_0.5ug_KO_48h.TIFF]

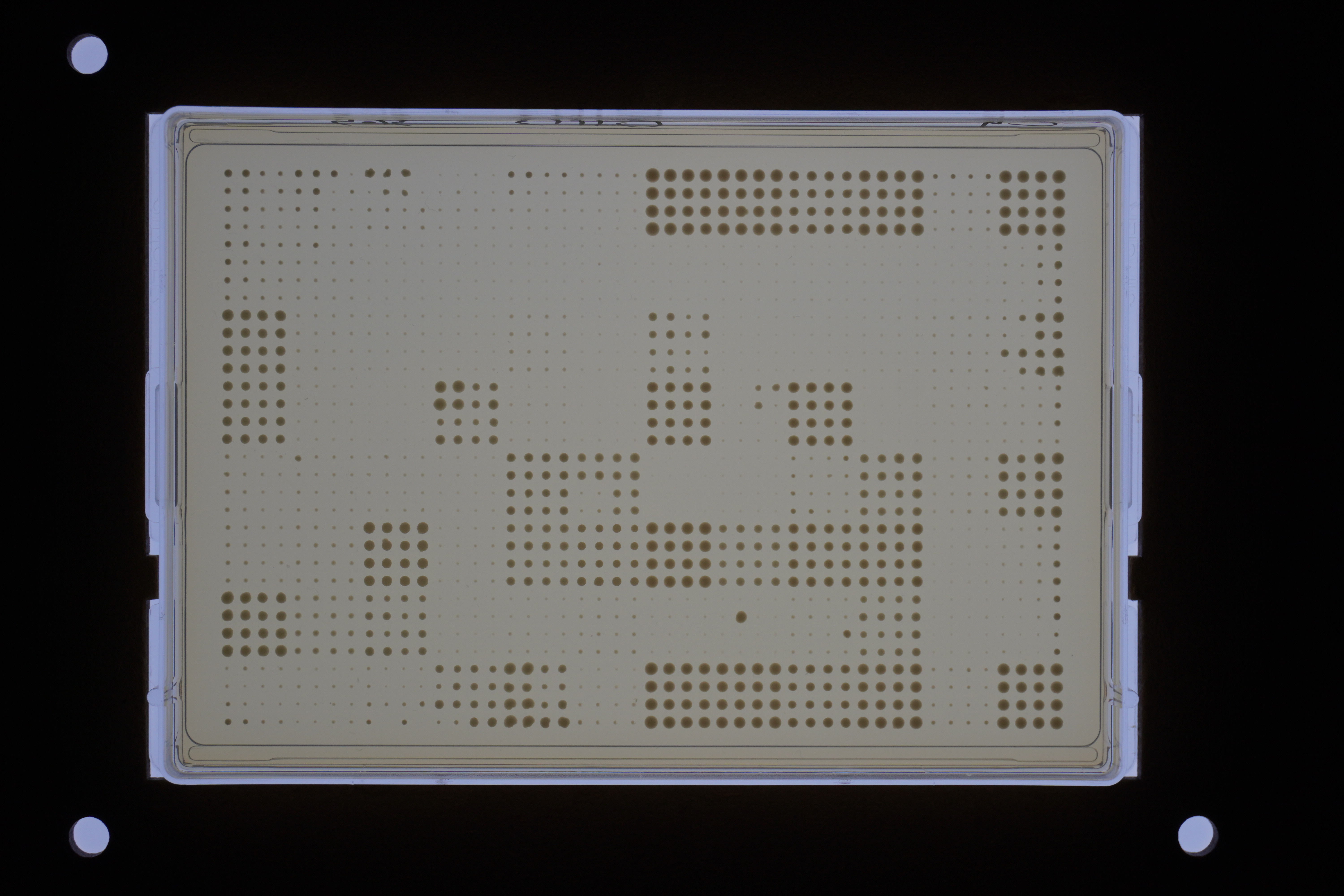

Supplement: Supplementary file 17 — Source data Fig. 5 [file 44319_2026_702_MOESM17_ESM.zip › Figure 5_SourceData/5C_to_5F/Images/Caspofungine_0.5ug_WT_48h.TIFF]

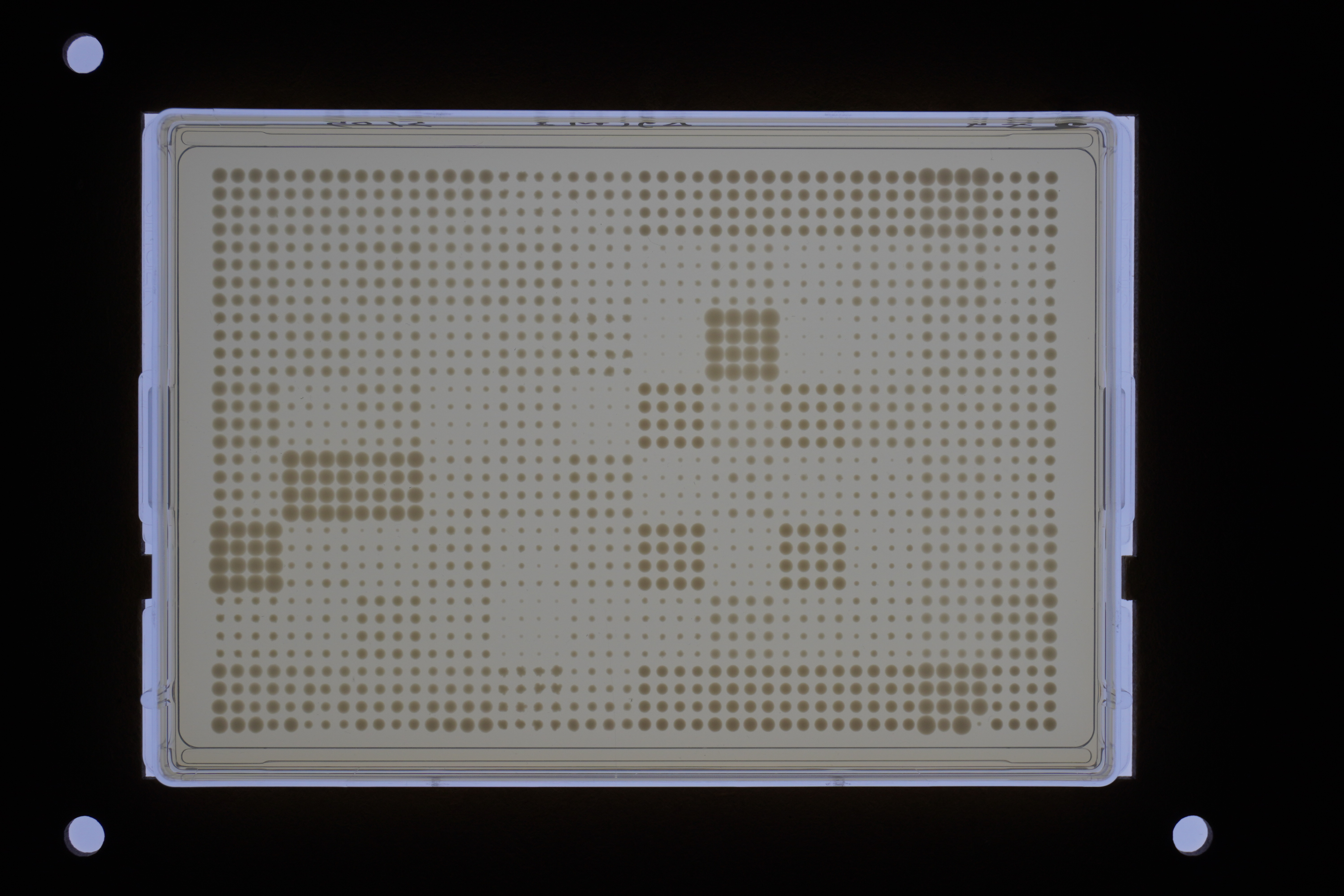

Supplement: Supplementary file 17 — Source data Fig. 5 [file 44319_2026_702_MOESM17_ESM.zip › Figure 5_SourceData/5C_to_5F/Images/Fluconazole_128ug_KO_48h.TIFF]

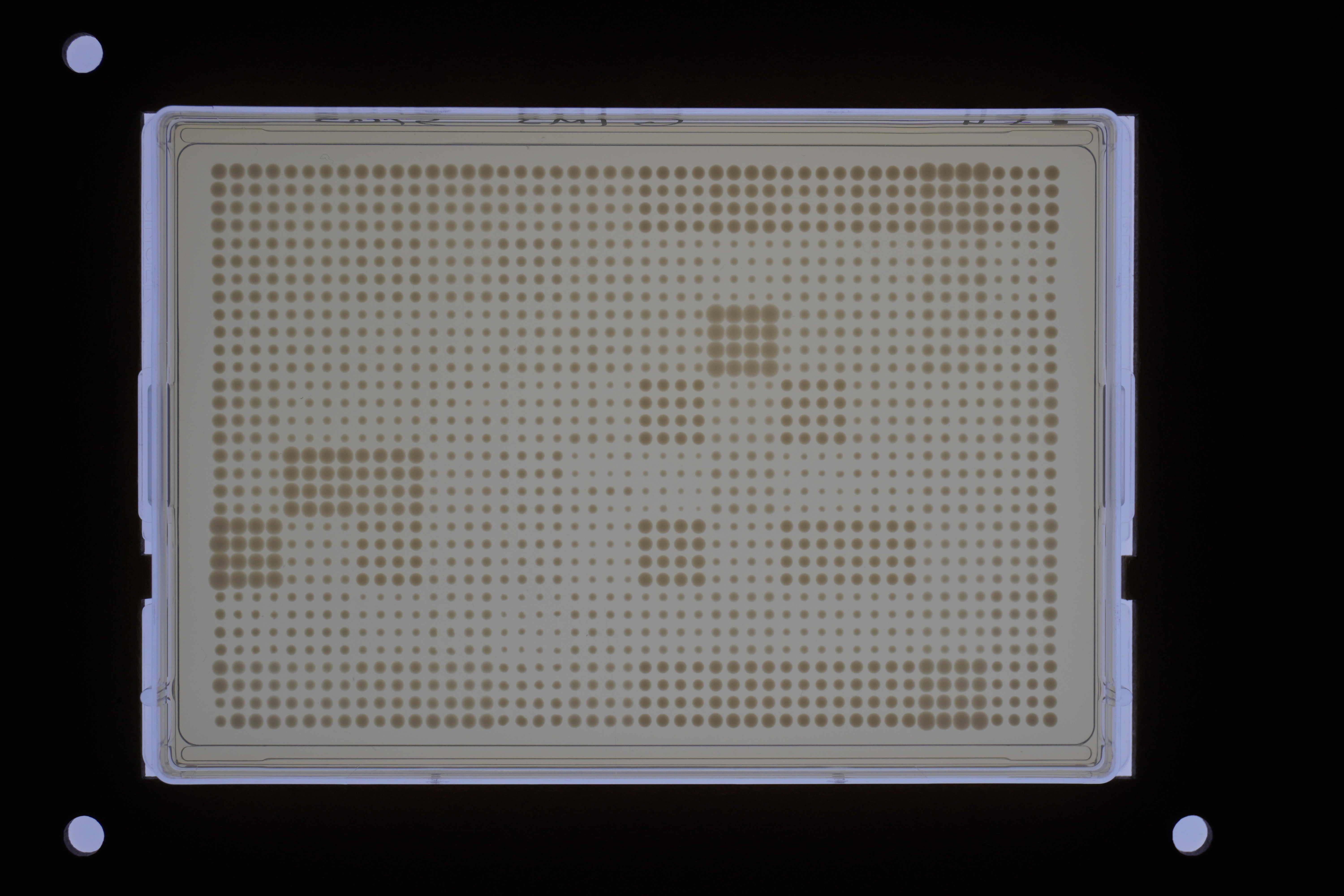

Supplement: Supplementary file 17 — Source data Fig. 5 [file 44319_2026_702_MOESM17_ESM.zip › Figure 5_SourceData/5C_to_5F/Images/Fluconazole_128ug_WT_48h.TIFF]

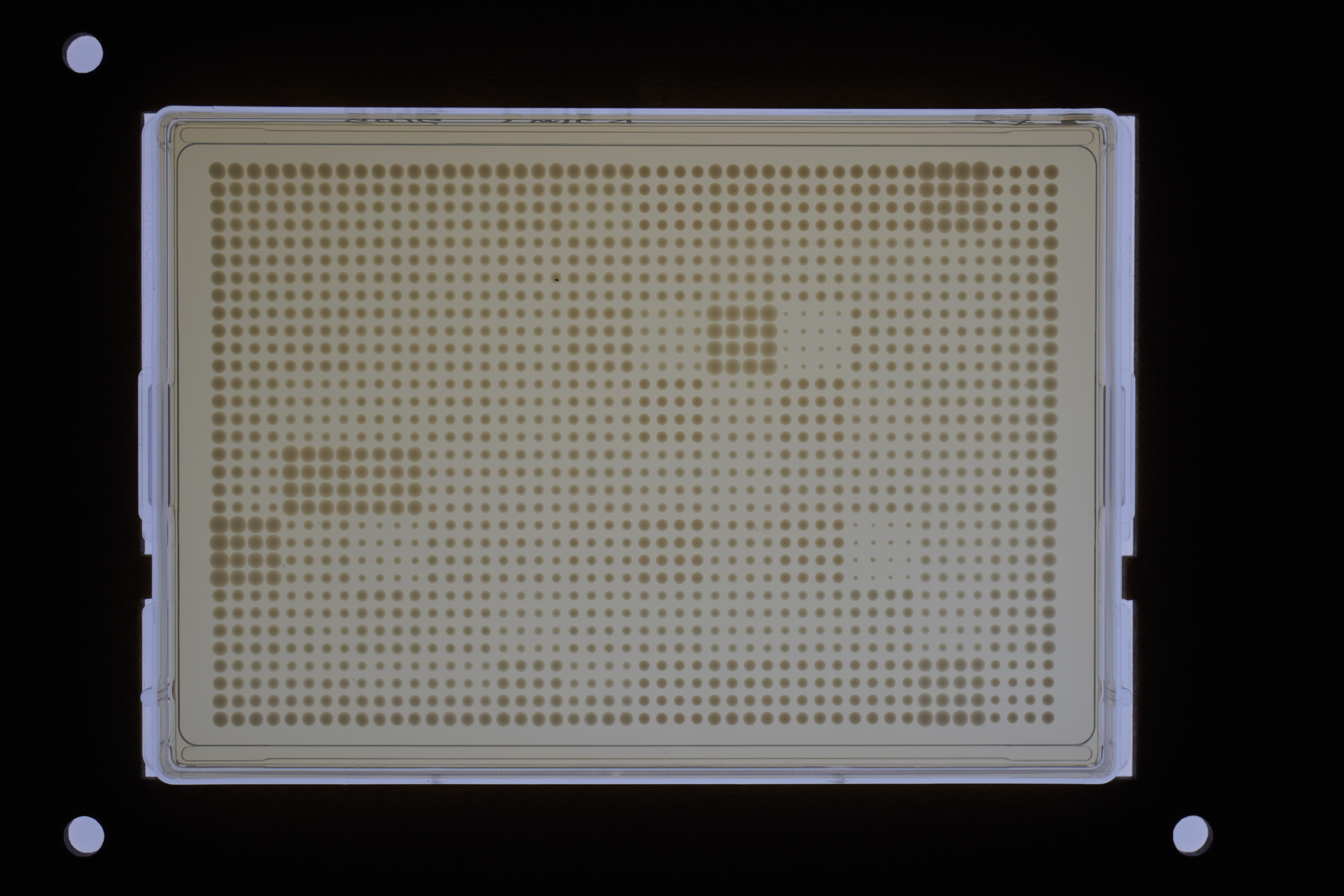

Supplement: Supplementary file 17 — Source data Fig. 5 [file 44319_2026_702_MOESM17_ESM.zip › Figure 5_SourceData/5C_to_5F/Images/Fluconazole_64ug_KO_48h.TIFF]

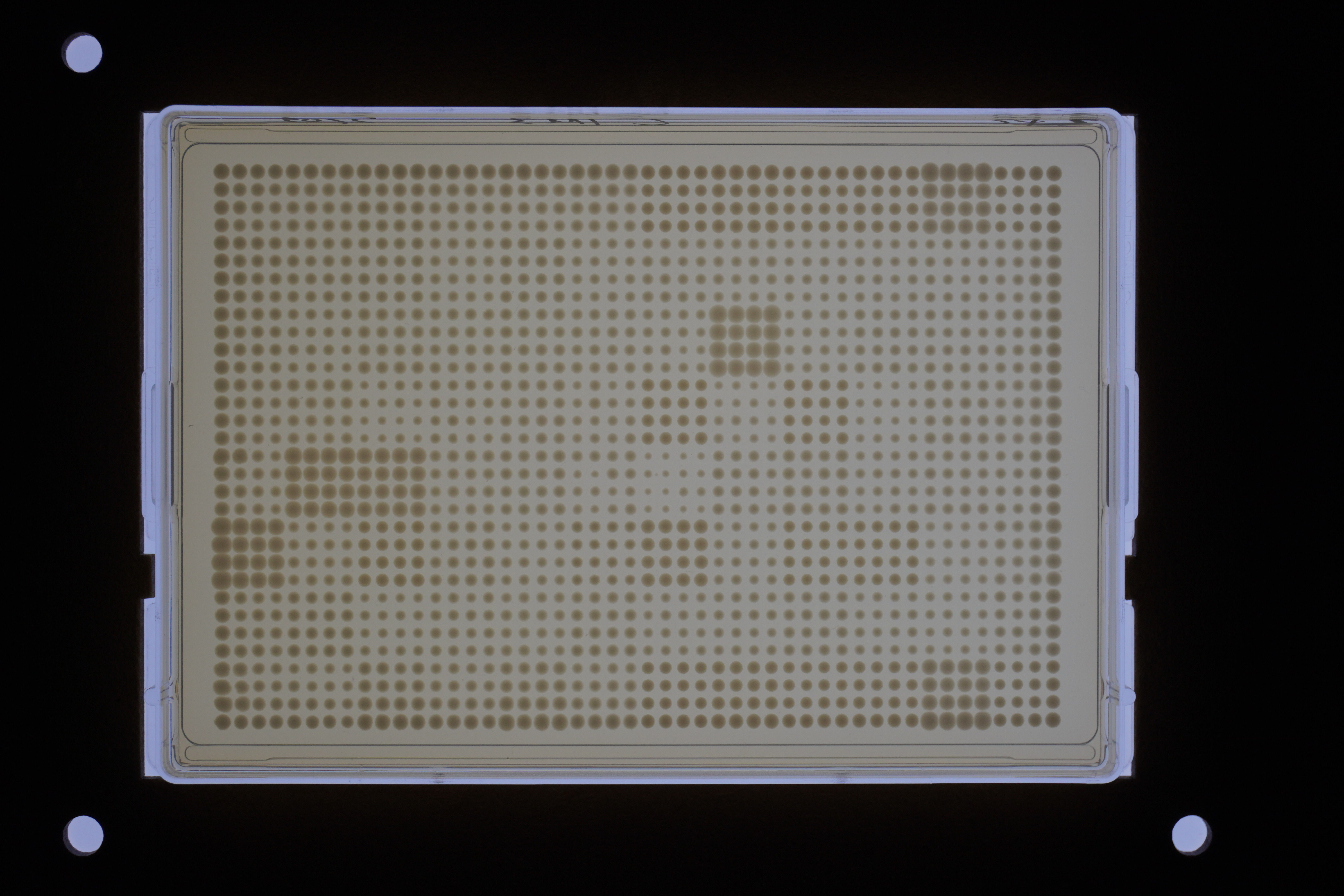

Supplement: Supplementary file 17 — Source data Fig. 5 [file 44319_2026_702_MOESM17_ESM.zip › Figure 5_SourceData/5C_to_5F/Images/Fluconazole_64ug_WT_48h.TIFF]

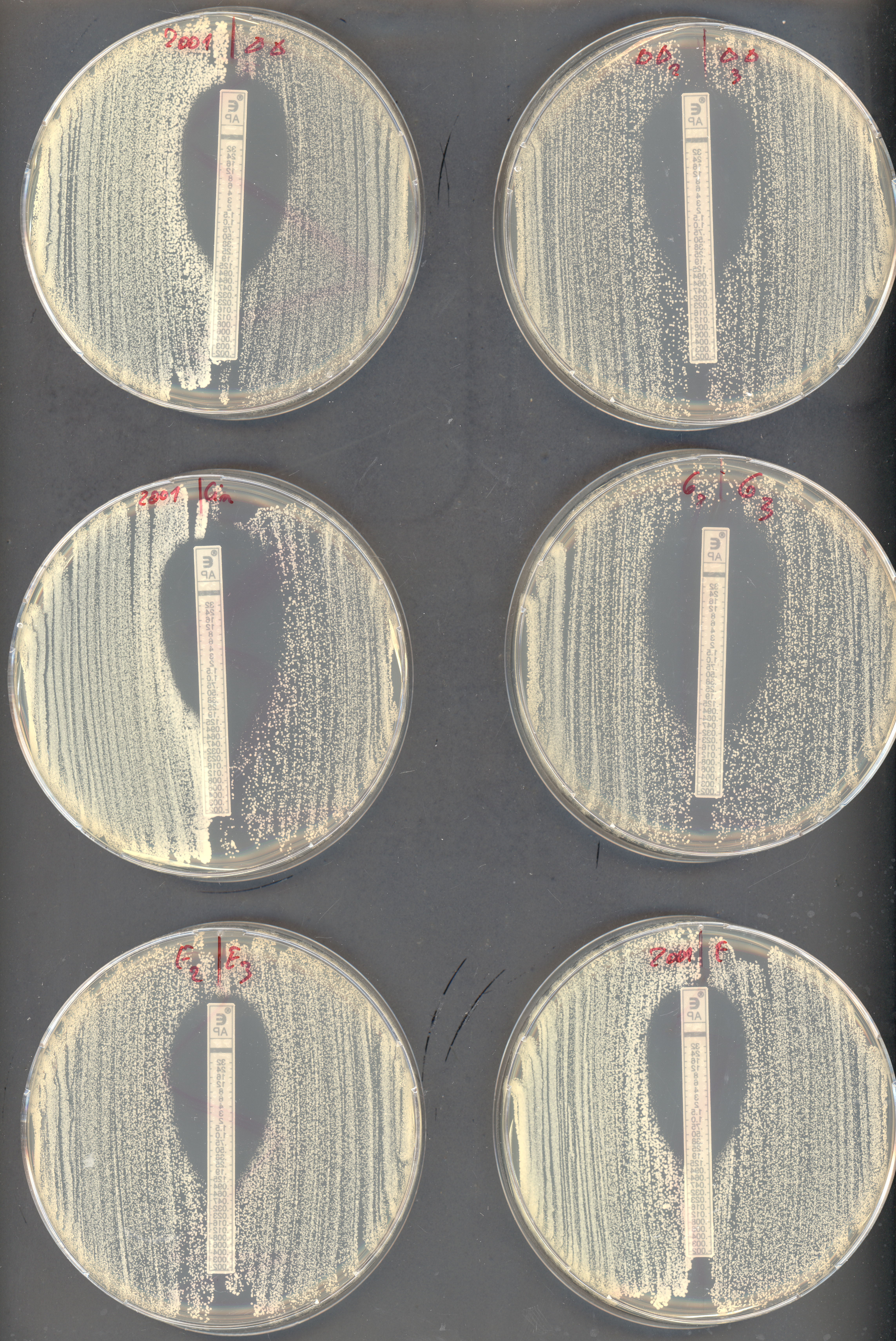

Supplement: Supplementary file 18 — Source data Fig. 6 [file 44319_2026_702_MOESM18_ESM.zip › Figure6_SourceData/Images 6B/Etest_Images_N glabratus/48hr/Nakaseomyces glabratus_AmpB.TIFF]

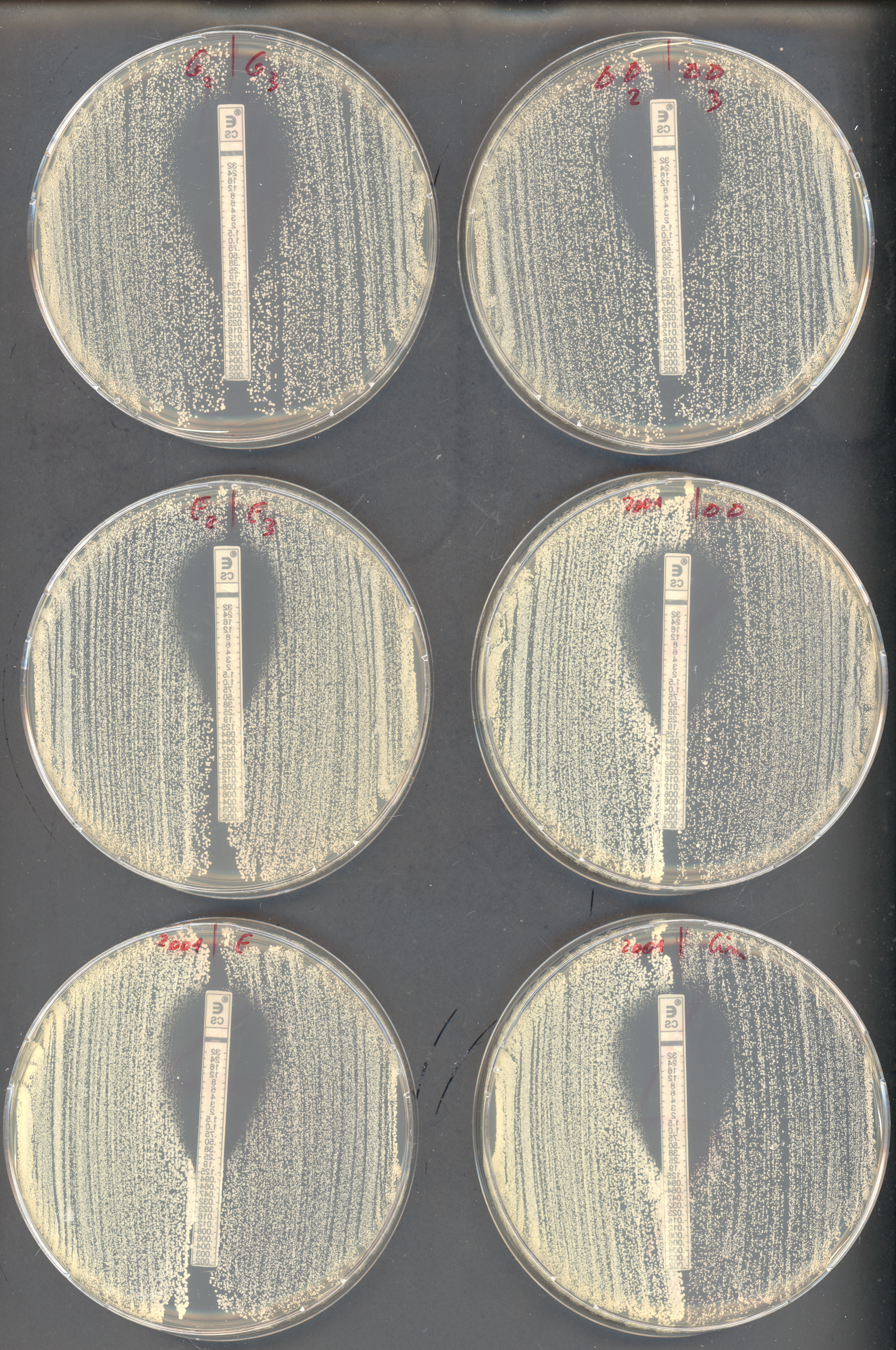

Supplement: Supplementary file 18 — Source data Fig. 6 [file 44319_2026_702_MOESM18_ESM.zip › Figure6_SourceData/Images 6B/Etest_Images_N glabratus/48hr/Nakaseomyces glabratus_Casp.TIFF]

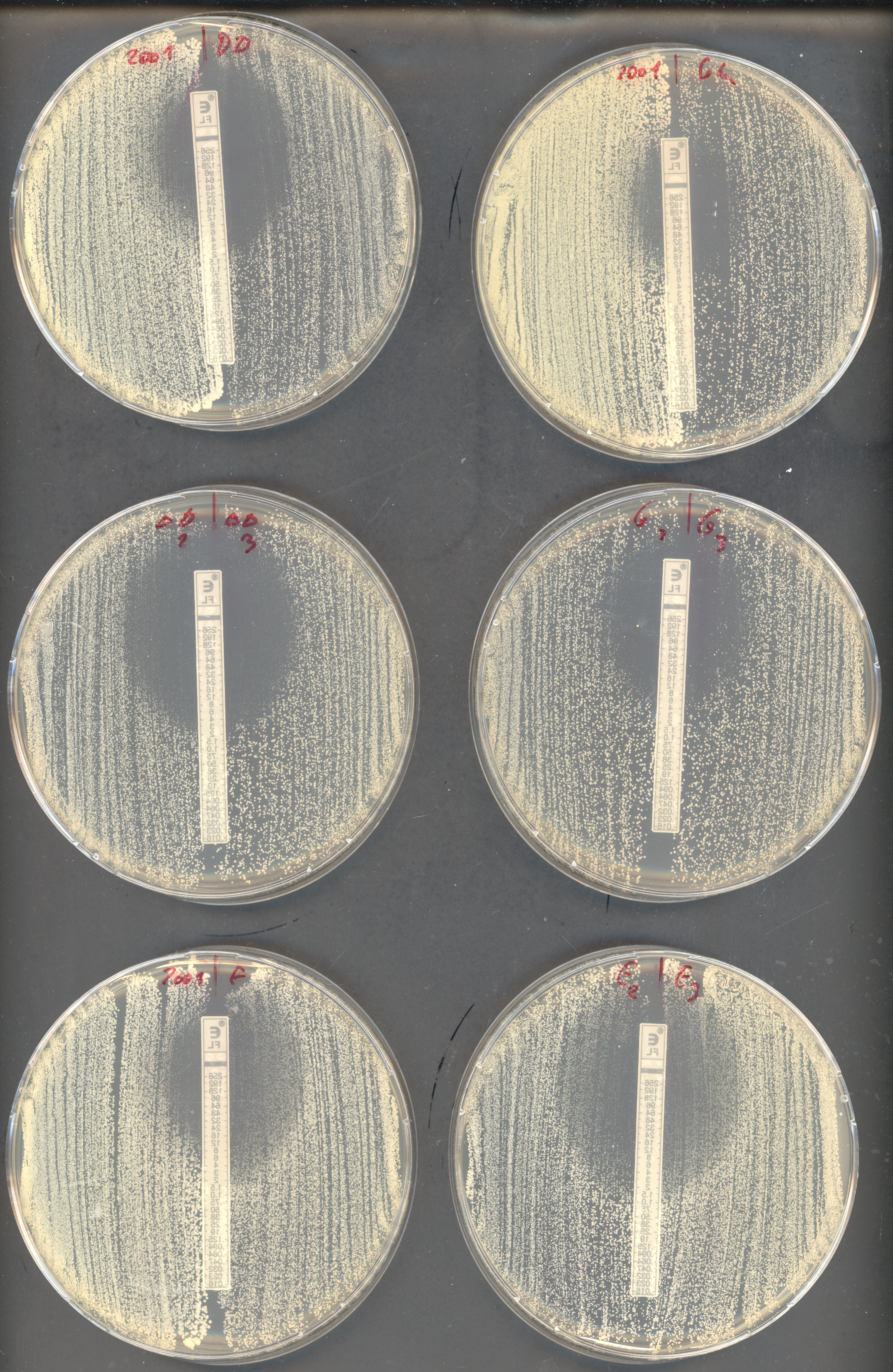

Supplement: Supplementary file 18 — Source data Fig. 6 [file 44319_2026_702_MOESM18_ESM.zip › Figure6_SourceData/Images 6B/Etest_Images_N glabratus/48hr/Nakaseomyces glabratus_Flc.TIFF]

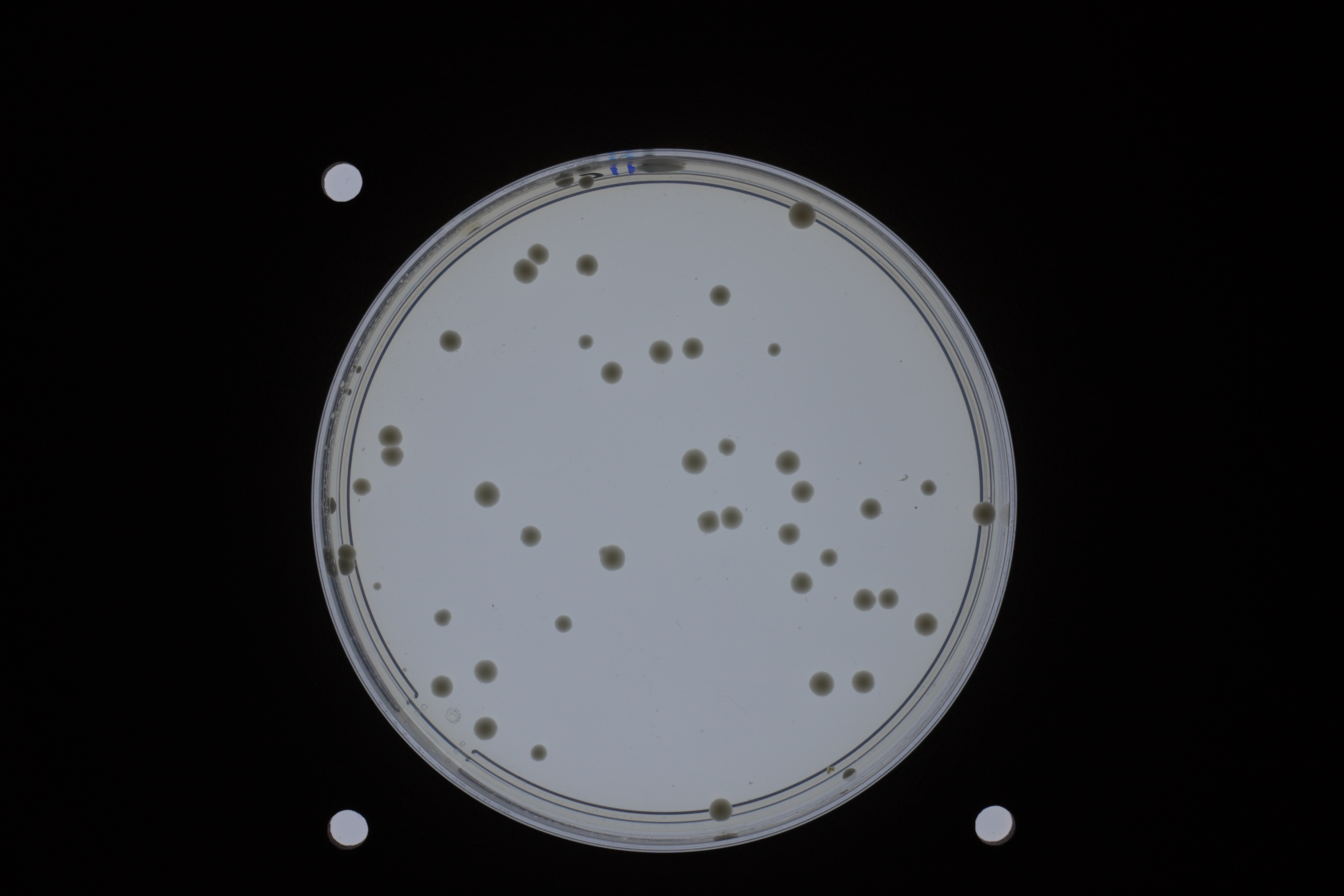

Supplement: Supplementary file 19 — Figure EV1 Source Data [file 44319_2026_702_MOESM19_ESM.zip › Figure EV1_SourceData/EV1A/Images/No fluconazole_H2O_Control_5FOA_1.TIFF]

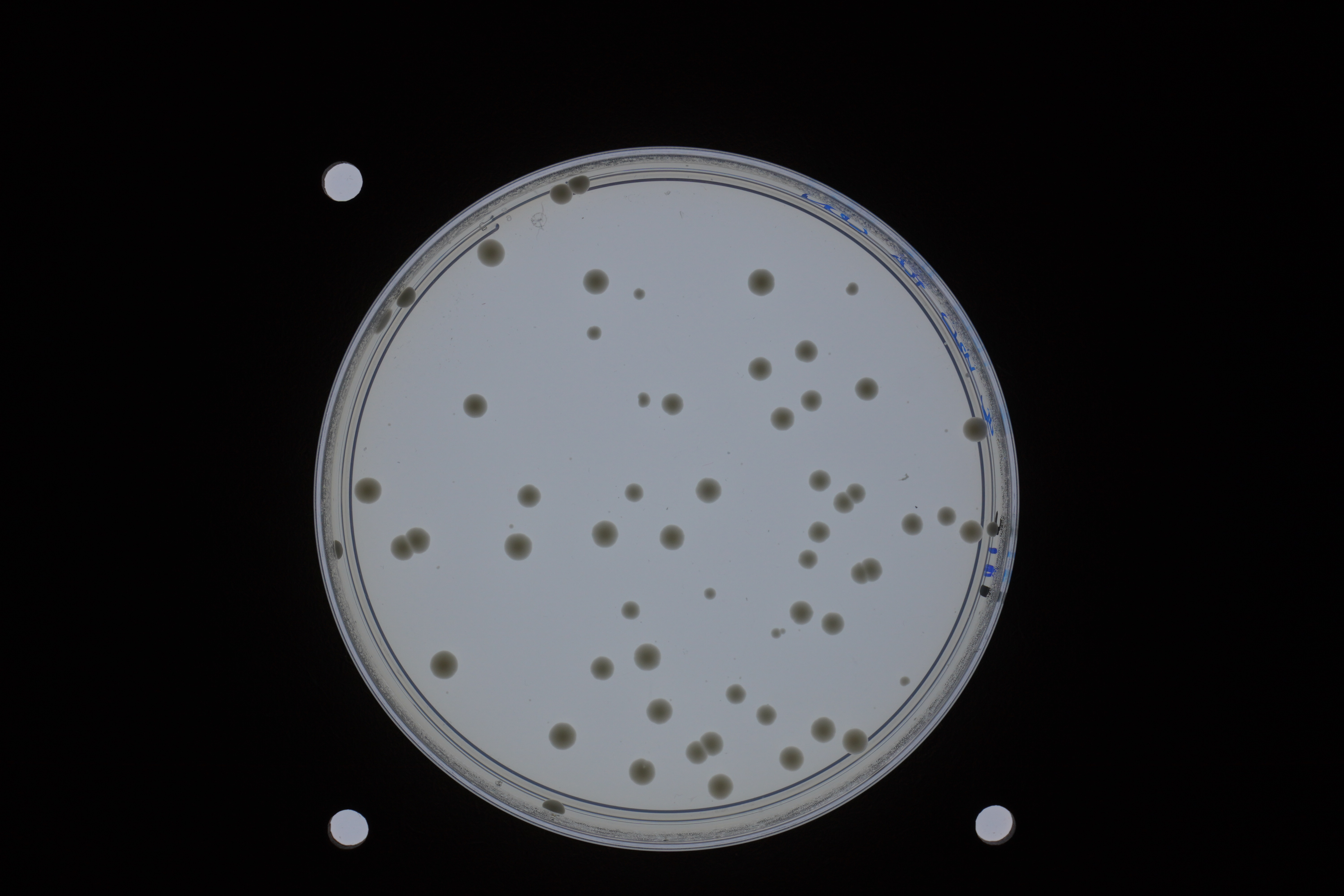

Supplement: Supplementary file 19 — Figure EV1 Source Data [file 44319_2026_702_MOESM19_ESM.zip › Figure EV1_SourceData/EV1A/Images/No fluconazole_H2O_Control_5FOA_10.TIFF]

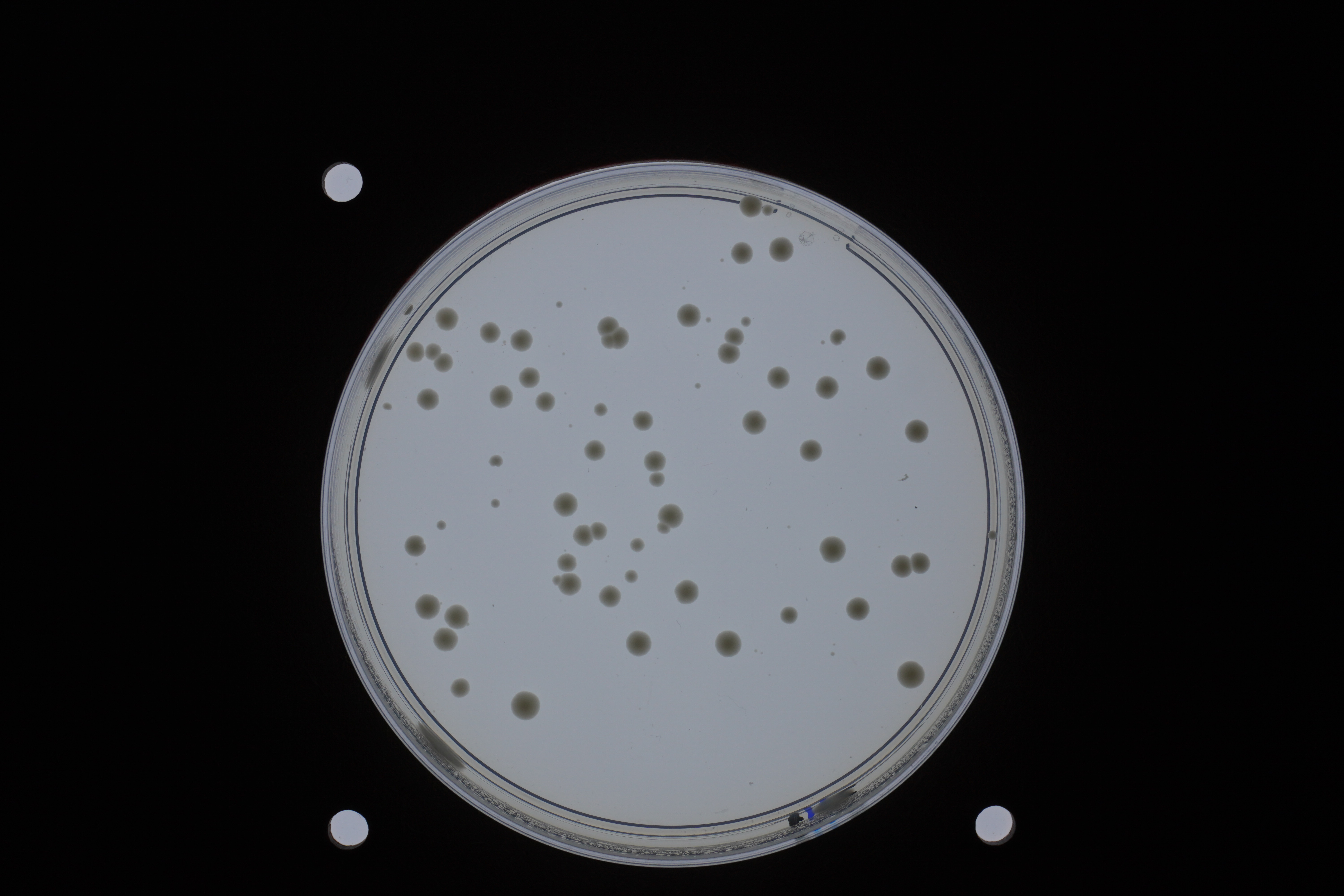

Supplement: Supplementary file 19 — Figure EV1 Source Data [file 44319_2026_702_MOESM19_ESM.zip › Figure EV1_SourceData/EV1A/Images/No fluconazole_H2O_Control_5FOA_2.TIFF]

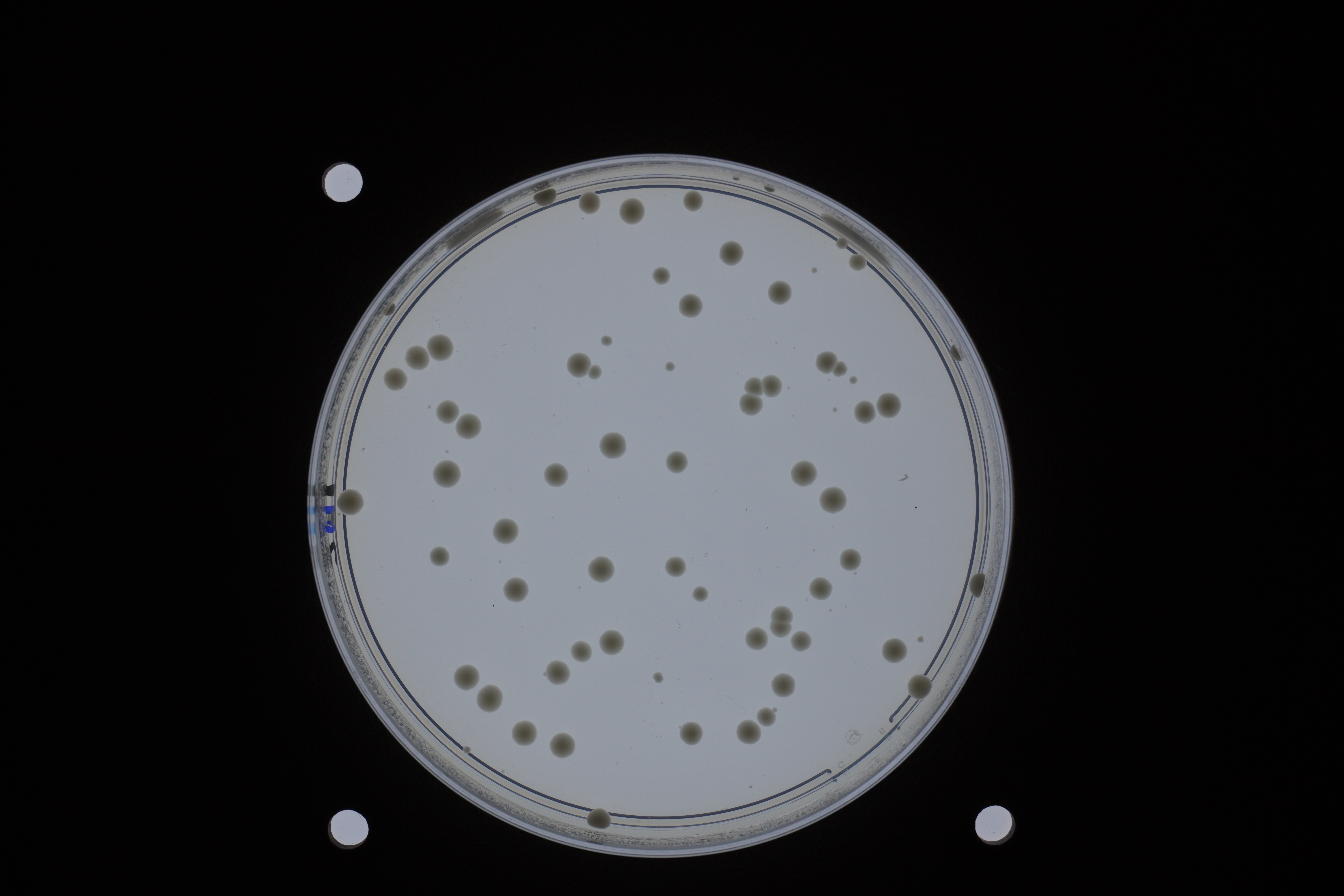

Supplement: Supplementary file 19 — Figure EV1 Source Data [file 44319_2026_702_MOESM19_ESM.zip › Figure EV1_SourceData/EV1A/Images/No fluconazole_H2O_Control_5FOA_3.TIFF]

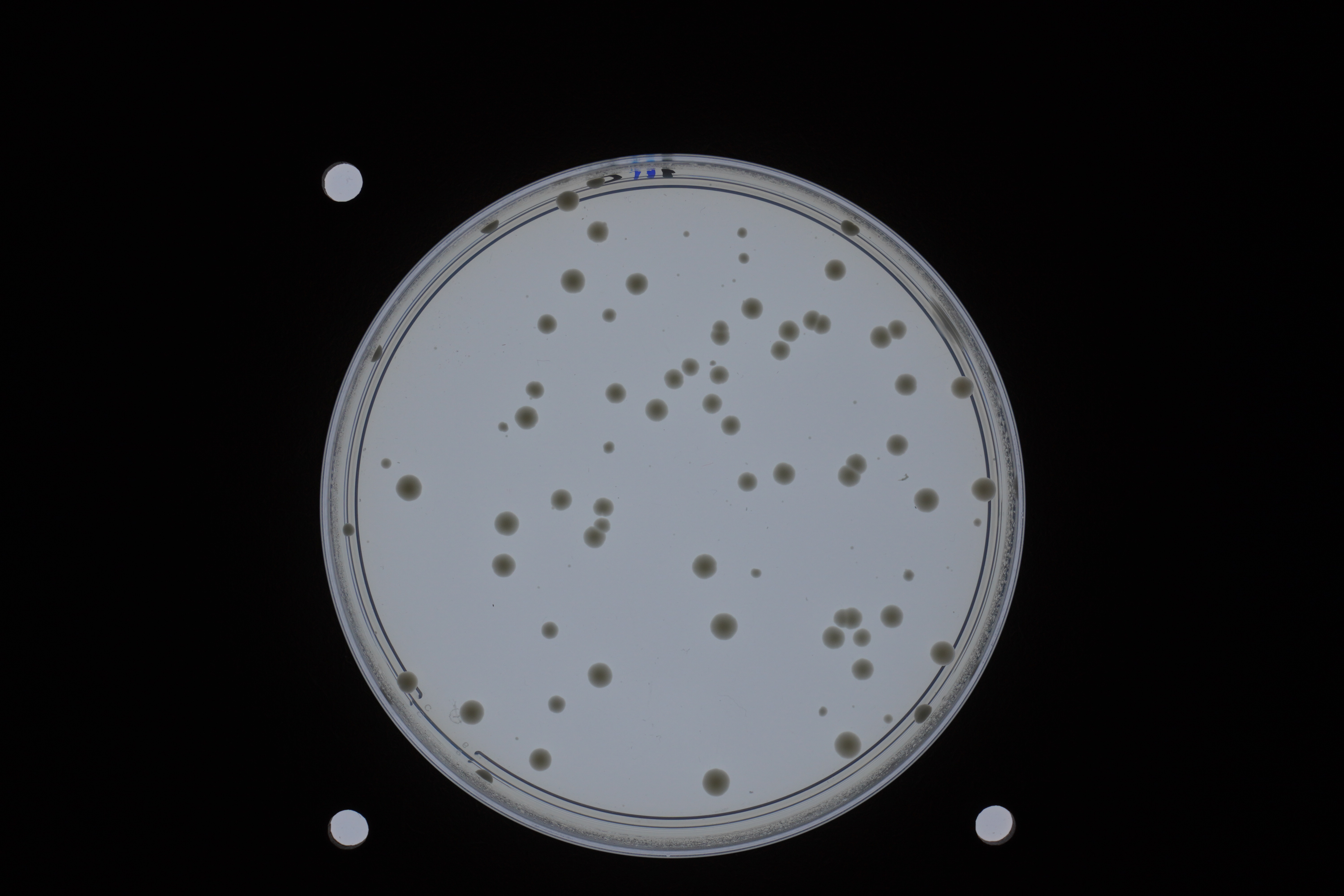

Supplement: Supplementary file 19 — Figure EV1 Source Data [file 44319_2026_702_MOESM19_ESM.zip › Figure EV1_SourceData/EV1A/Images/No fluconazole_H2O_Control_5FOA_4.TIFF]

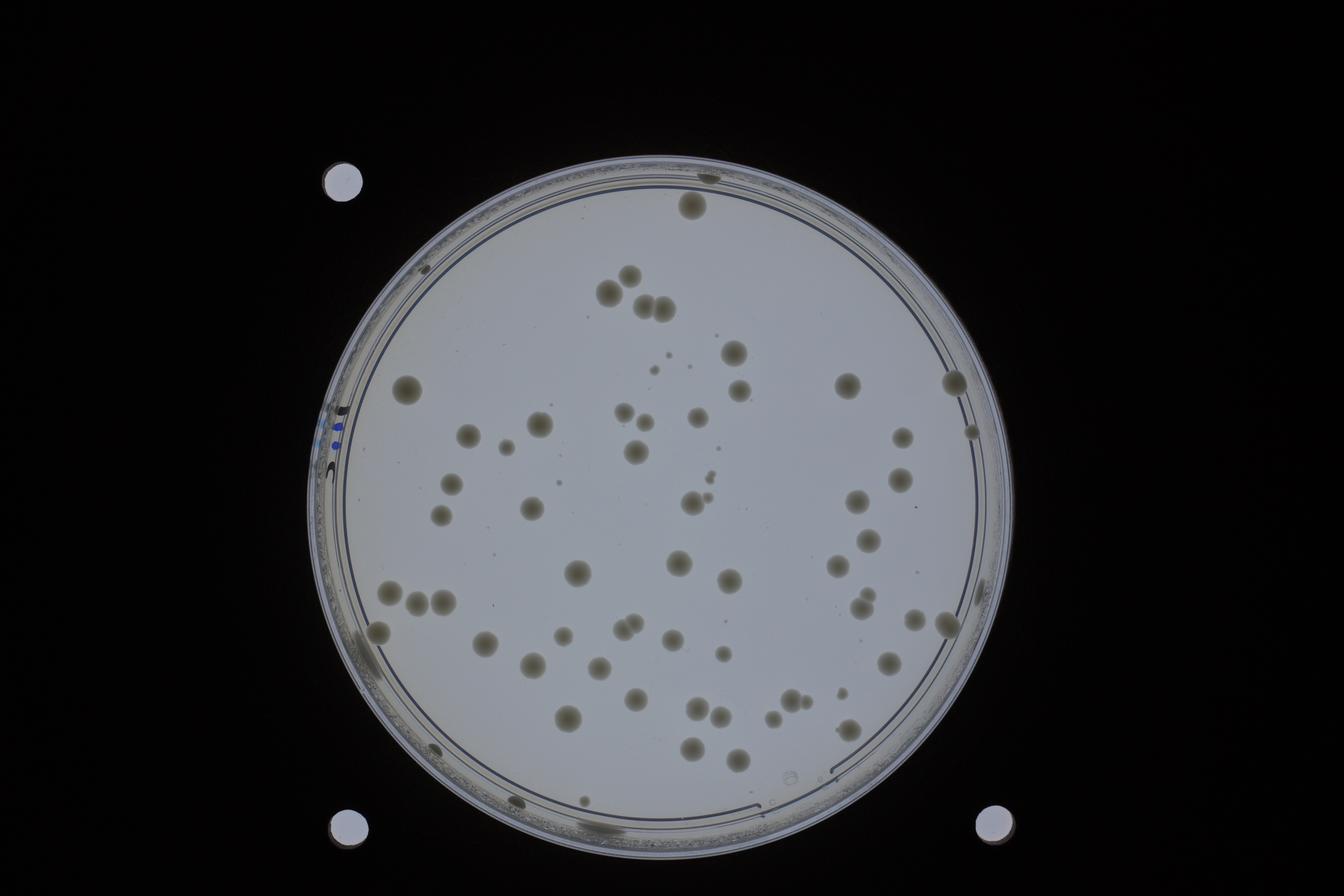

Supplement: Supplementary file 19 — Figure EV1 Source Data [file 44319_2026_702_MOESM19_ESM.zip › Figure EV1_SourceData/EV1A/Images/No fluconazole_H2O_Control_5FOA_5.TIFF]

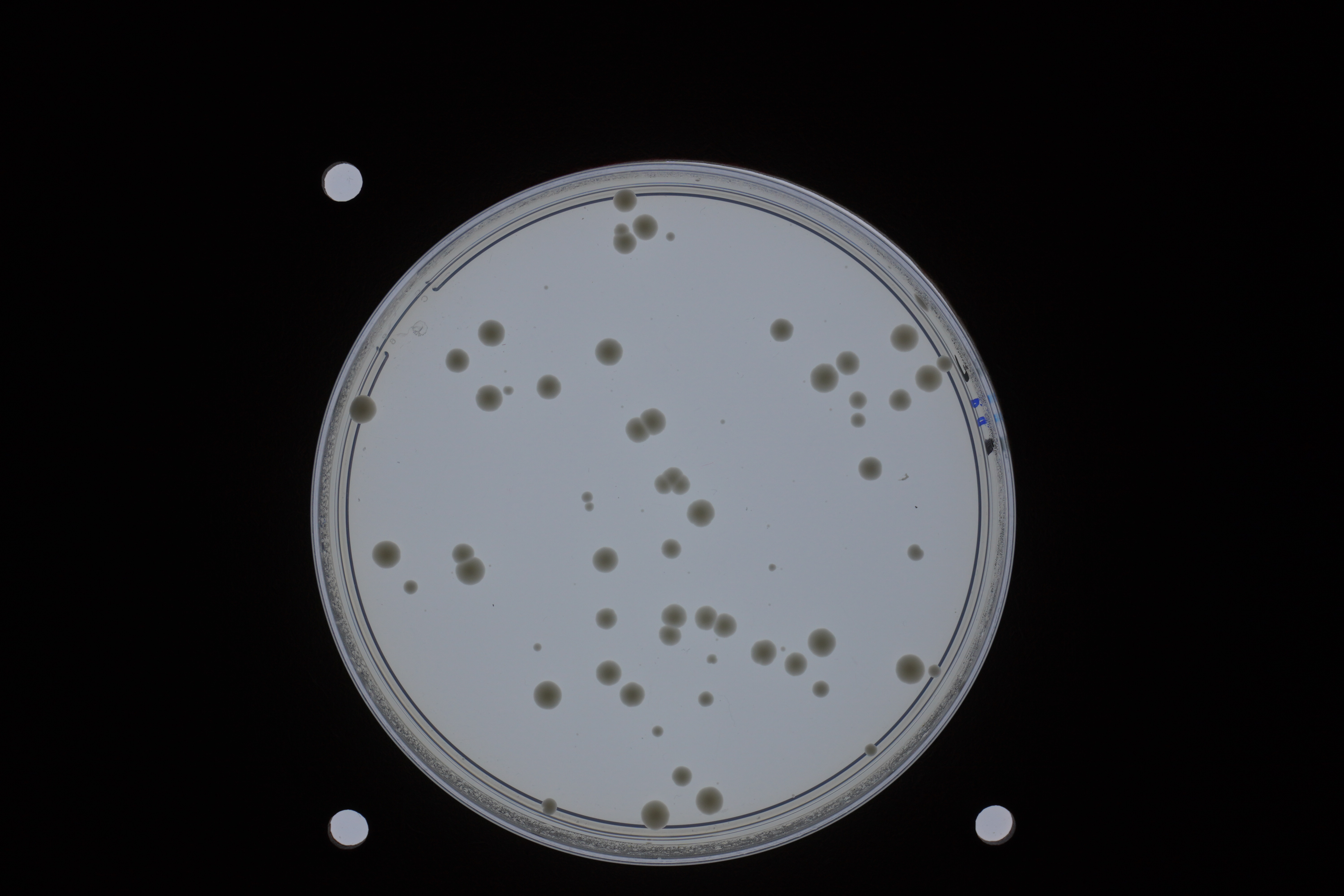

Supplement: Supplementary file 19 — Figure EV1 Source Data [file 44319_2026_702_MOESM19_ESM.zip › Figure EV1_SourceData/EV1A/Images/No fluconazole_H2O_Control_5FOA_6.TIFF]

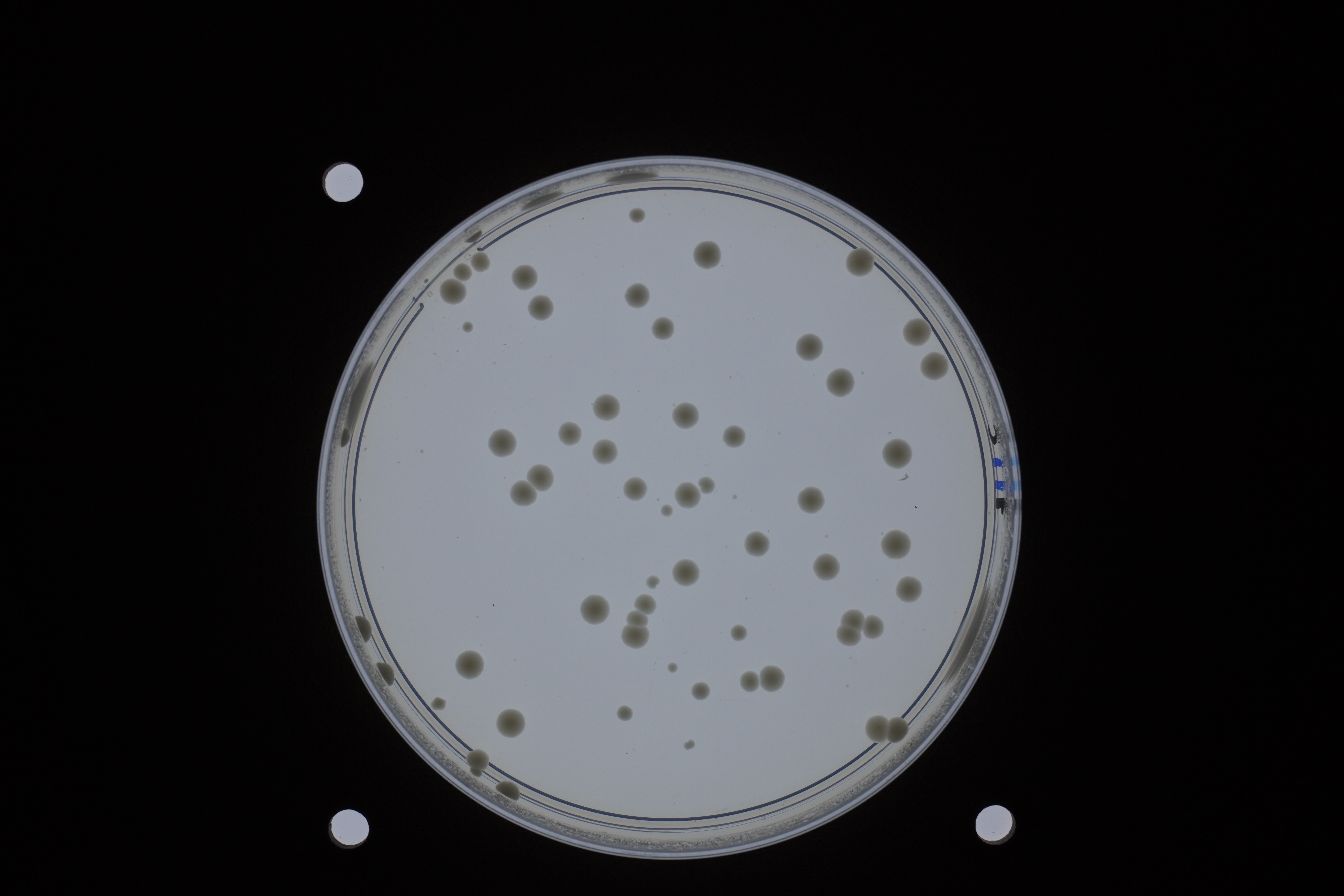

Supplement: Supplementary file 19 — Figure EV1 Source Data [file 44319_2026_702_MOESM19_ESM.zip › Figure EV1_SourceData/EV1A/Images/No fluconazole_H2O_Control_5FOA_7.TIFF]

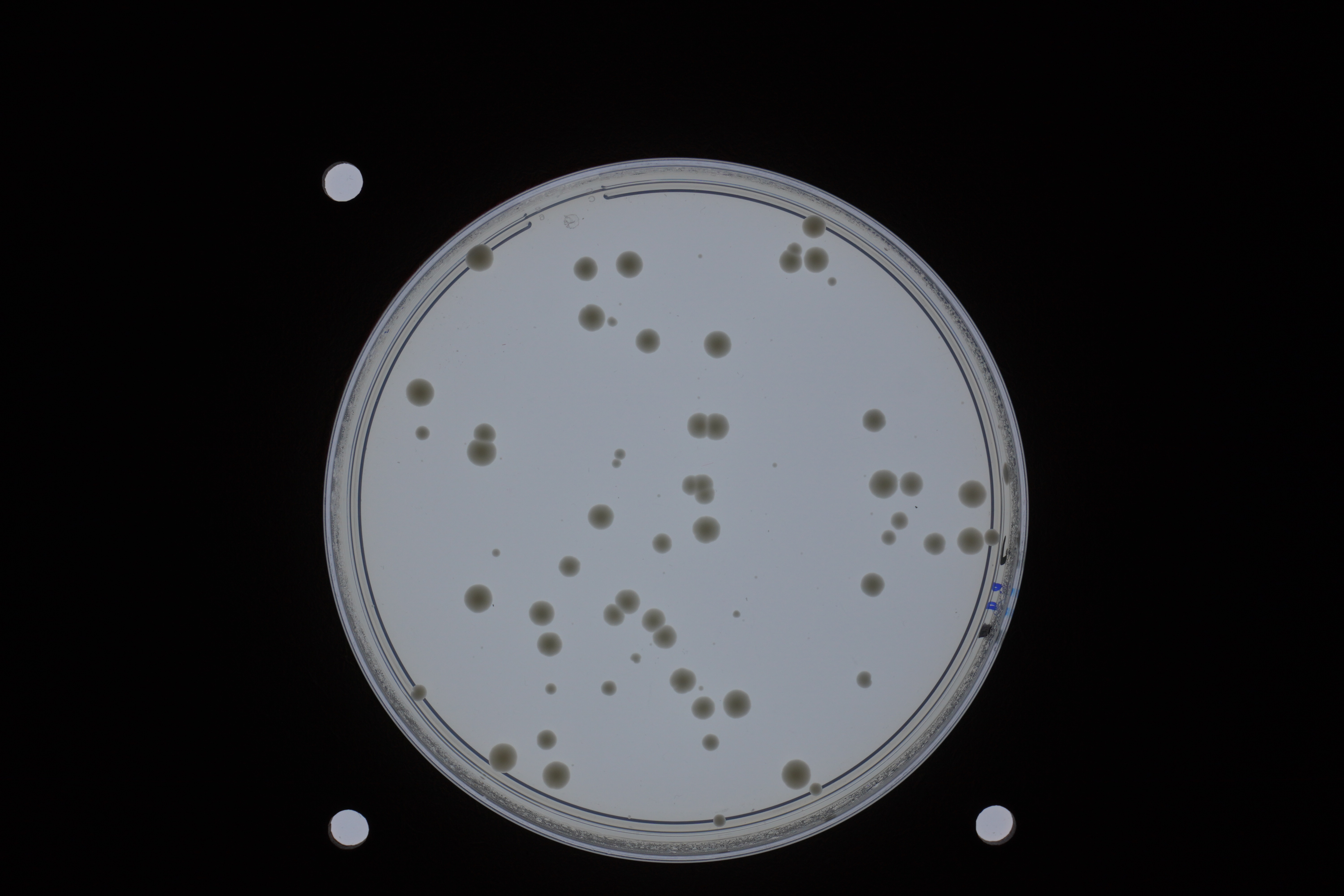

Supplement: Supplementary file 19 — Figure EV1 Source Data [file 44319_2026_702_MOESM19_ESM.zip › Figure EV1_SourceData/EV1A/Images/No fluconazole_H2O_Control_5FOA_8.TIFF]

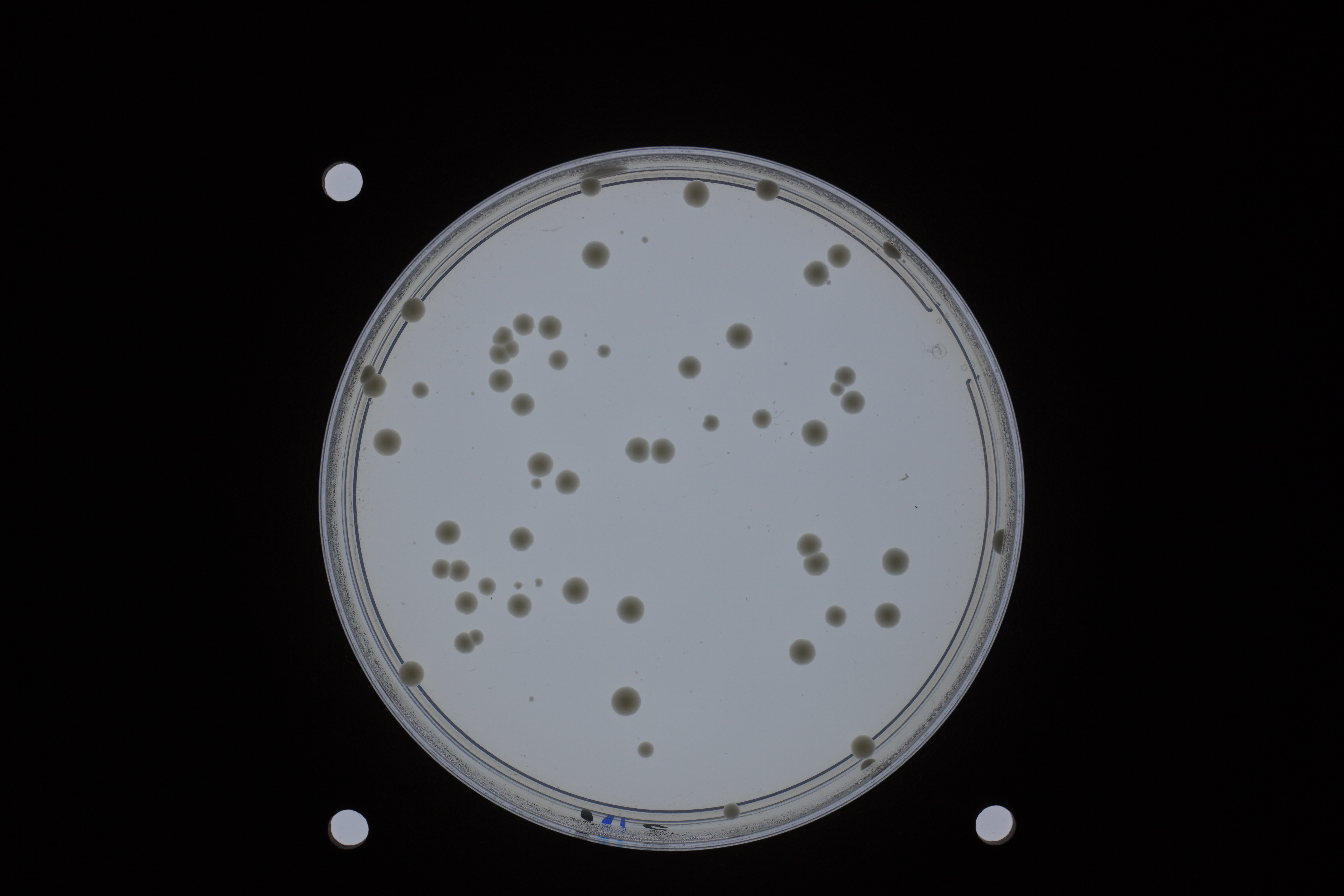

Supplement: Supplementary file 19 — Figure EV1 Source Data [file 44319_2026_702_MOESM19_ESM.zip › Figure EV1_SourceData/EV1A/Images/No fluconazole_H2O_Control_5FOA_9.TIFF]

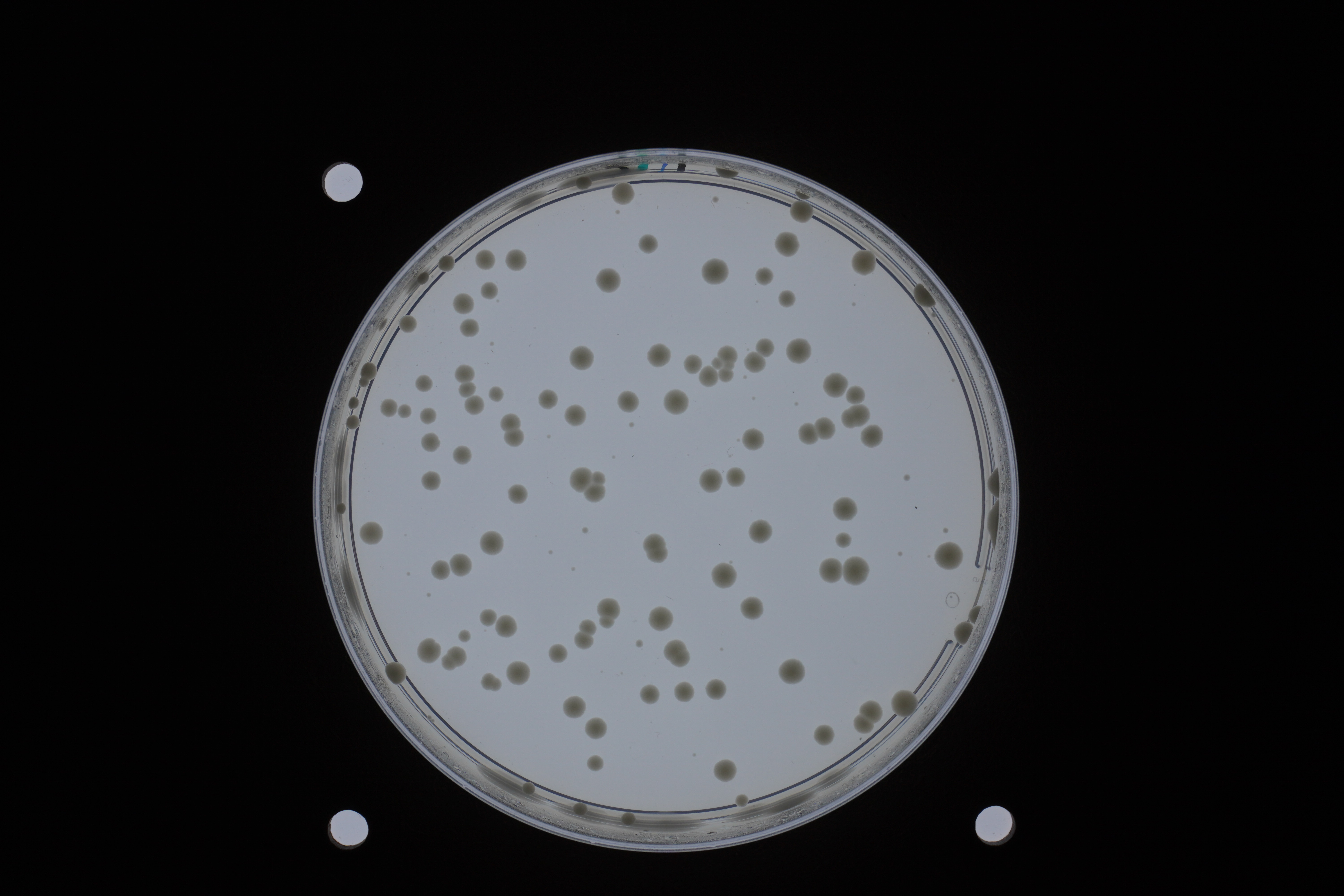

Supplement: Supplementary file 19 — Figure EV1 Source Data [file 44319_2026_702_MOESM19_ESM.zip › Figure EV1_SourceData/EV1A/Images/No fluconazole_H2O_Control_SCmURA_1.TIFF]

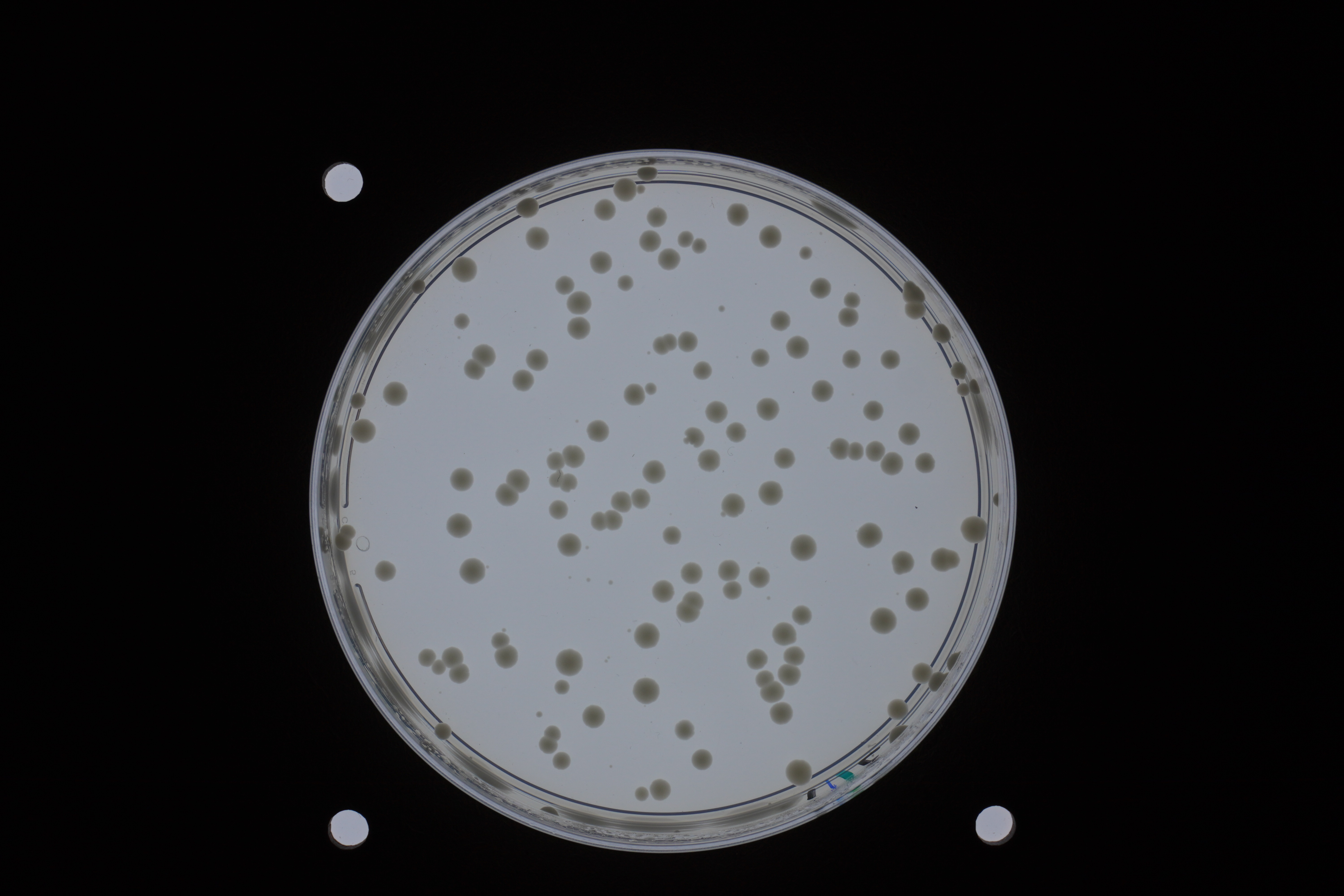

Supplement: Supplementary file 19 — Figure EV1 Source Data [file 44319_2026_702_MOESM19_ESM.zip › Figure EV1_SourceData/EV1A/Images/No fluconazole_H2O_Control_SCmURA_10.TIFF]

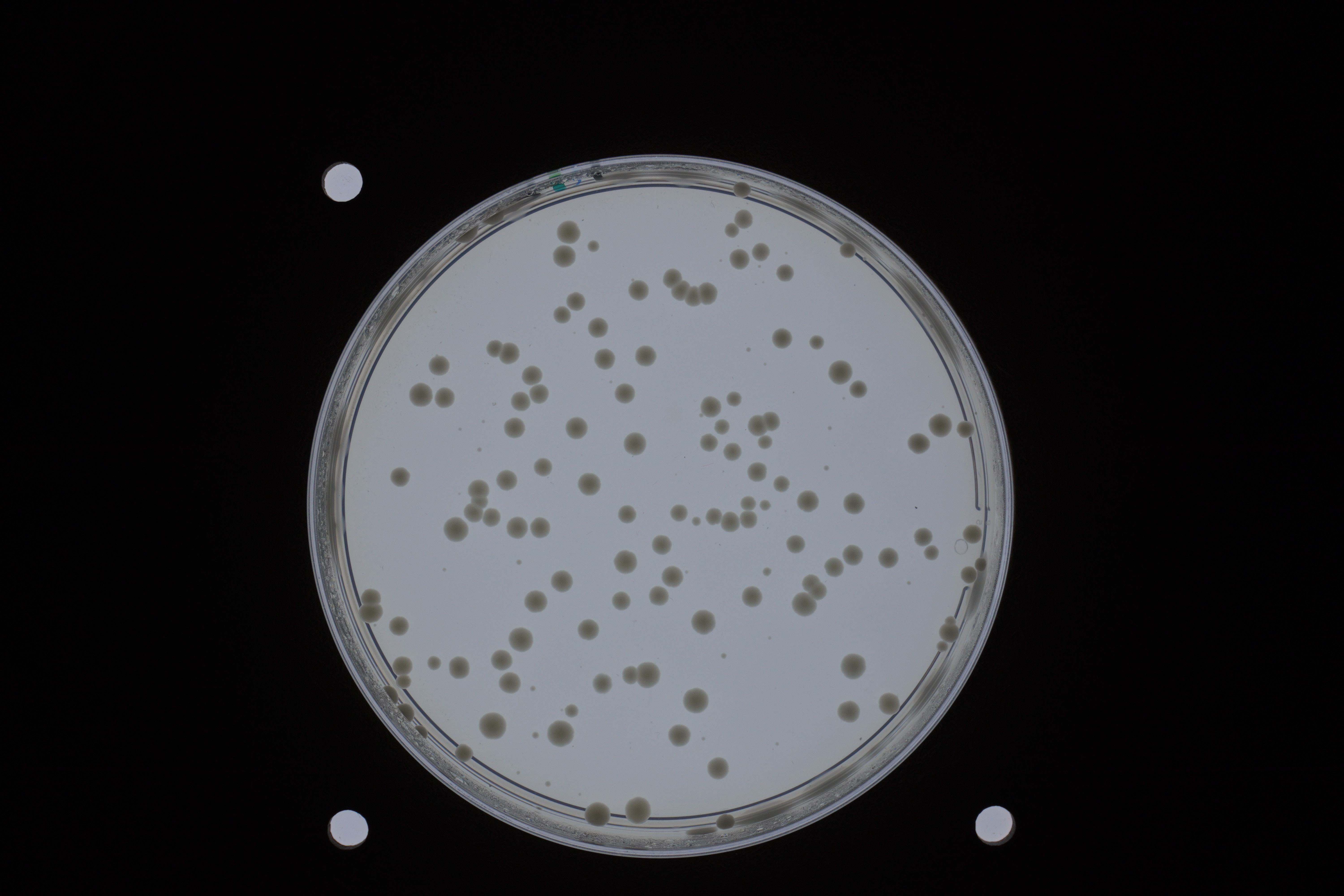

Supplement: Supplementary file 19 — Figure EV1 Source Data [file 44319_2026_702_MOESM19_ESM.zip › Figure EV1_SourceData/EV1A/Images/No fluconazole_H2O_Control_SCmURA_2.TIFF]

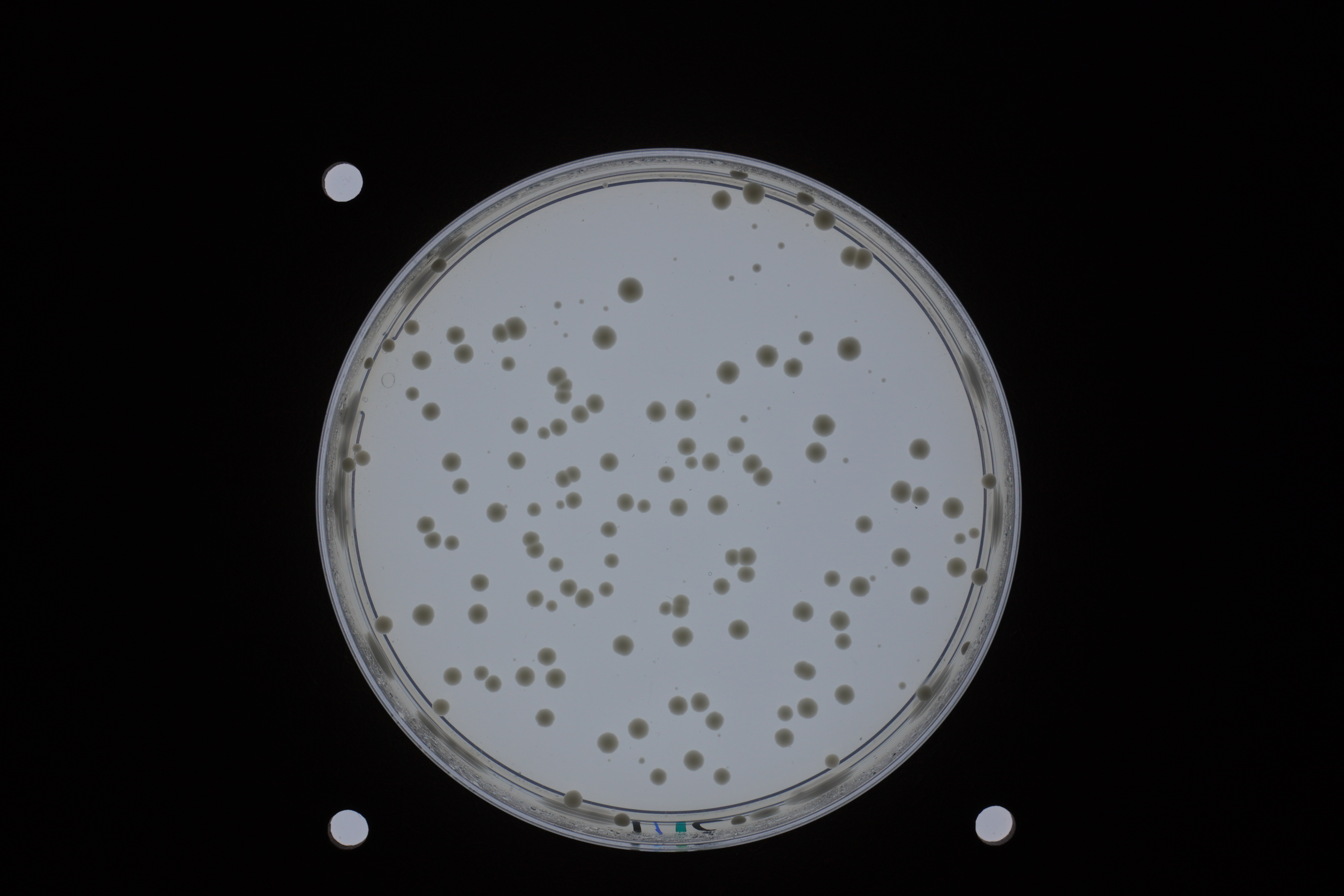

Supplement: Supplementary file 19 — Figure EV1 Source Data [file 44319_2026_702_MOESM19_ESM.zip › Figure EV1_SourceData/EV1A/Images/No fluconazole_H2O_Control_SCmURA_3.TIFF]

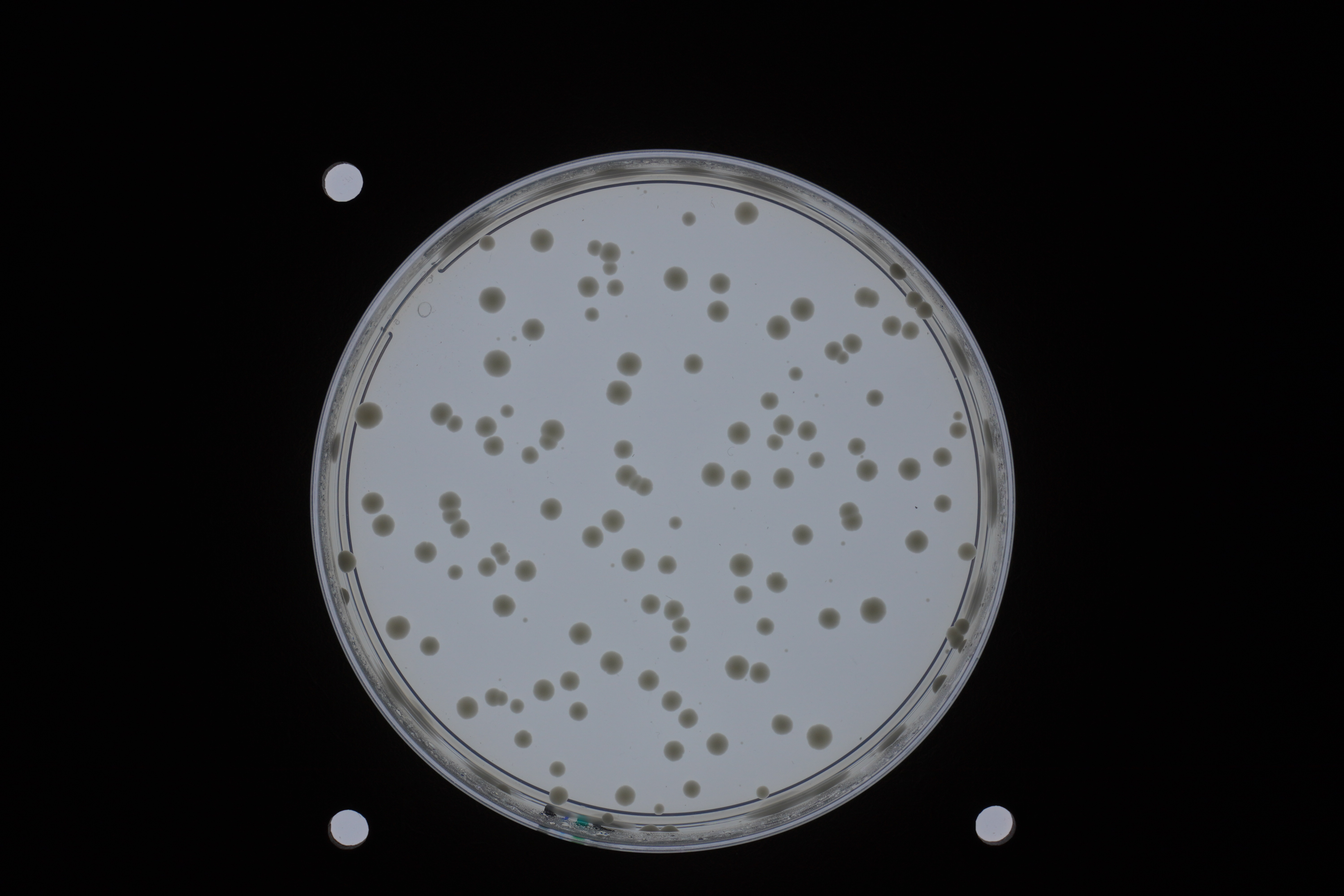

Supplement: Supplementary file 19 — Figure EV1 Source Data [file 44319_2026_702_MOESM19_ESM.zip › Figure EV1_SourceData/EV1A/Images/No fluconazole_H2O_Control_SCmURA_4.TIFF]

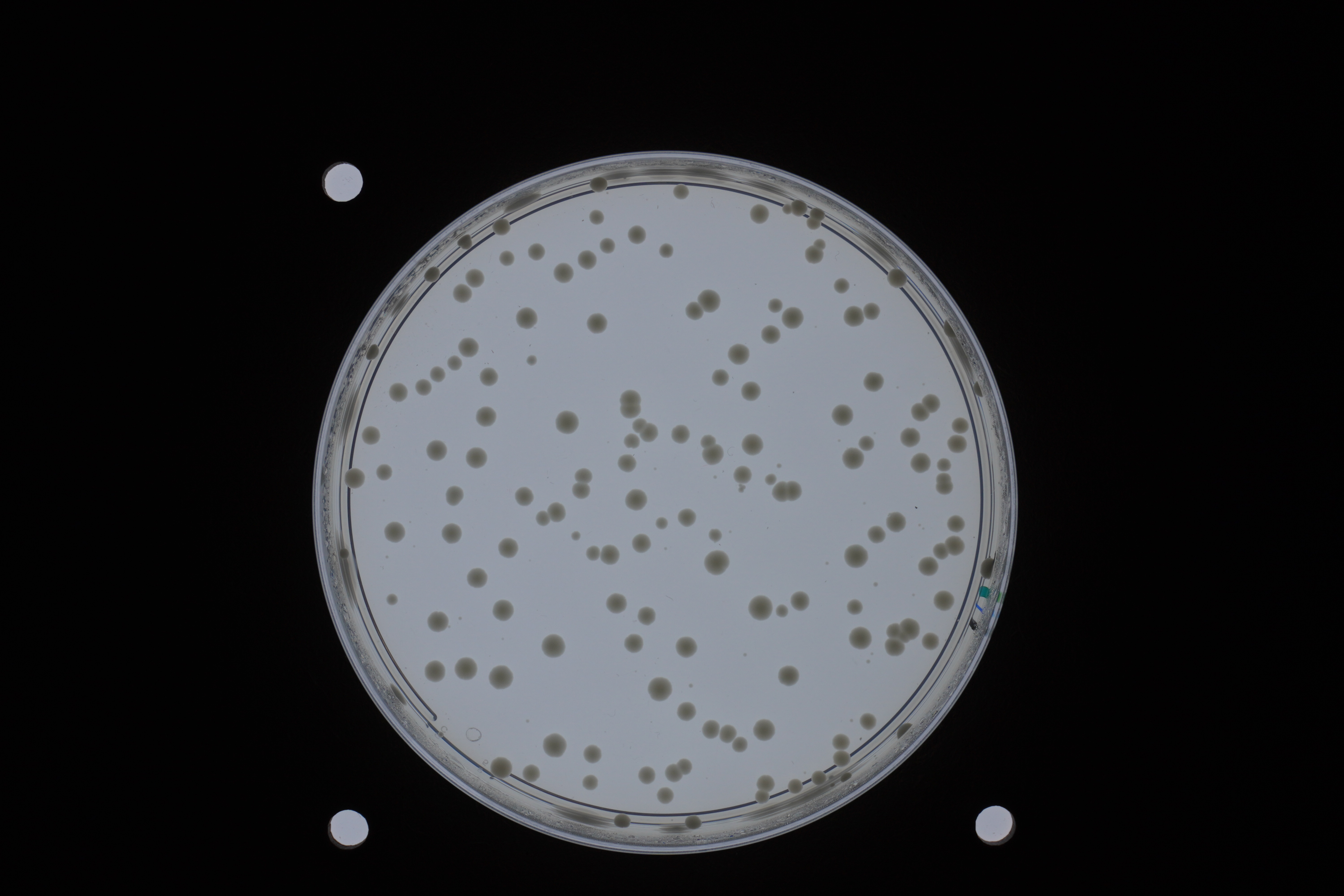

Supplement: Supplementary file 19 — Figure EV1 Source Data [file 44319_2026_702_MOESM19_ESM.zip › Figure EV1_SourceData/EV1A/Images/No fluconazole_H2O_Control_SCmURA_5.TIFF]

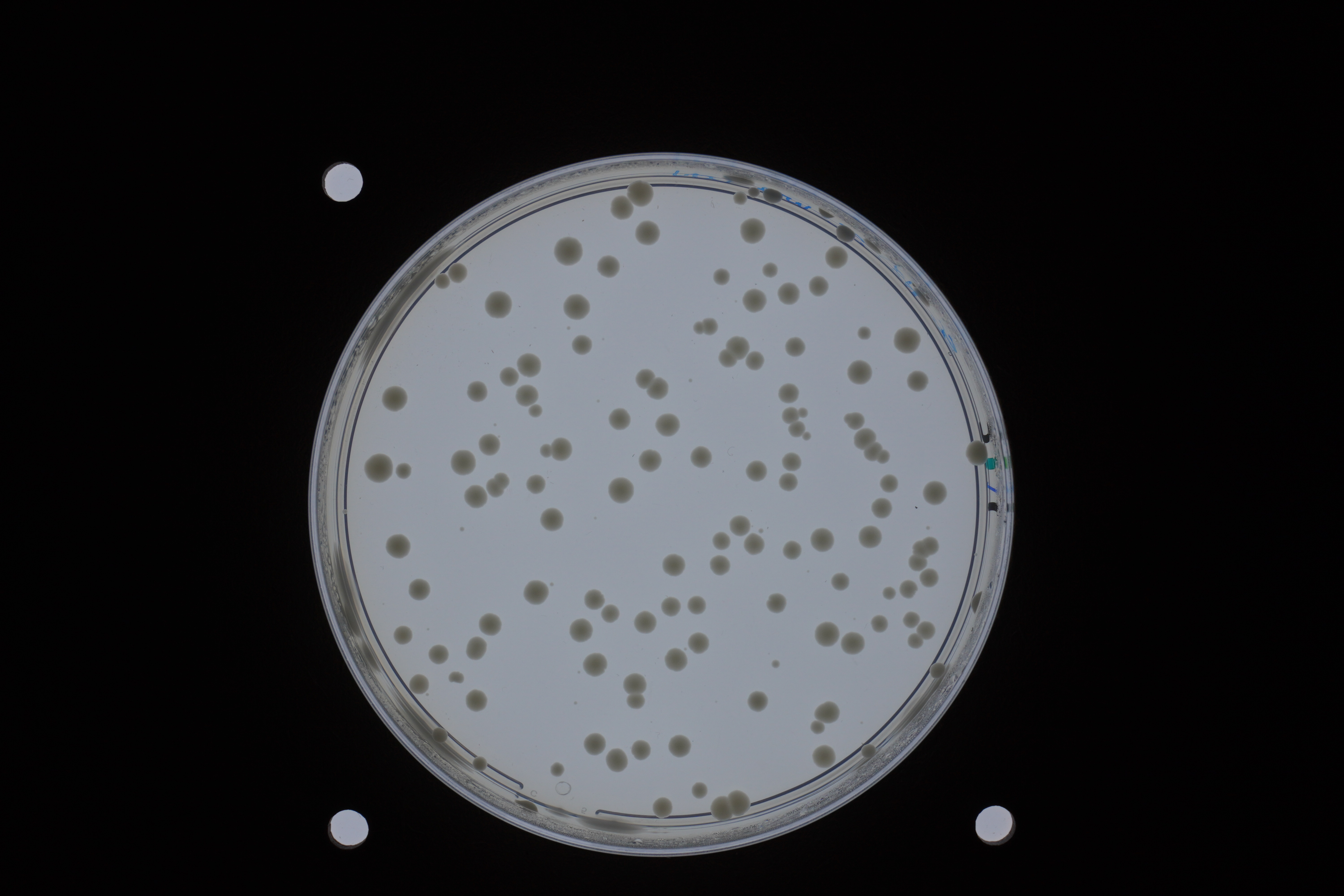

Supplement: Supplementary file 19 — Figure EV1 Source Data [file 44319_2026_702_MOESM19_ESM.zip › Figure EV1_SourceData/EV1A/Images/No fluconazole_H2O_Control_SCmURA_6.TIFF]

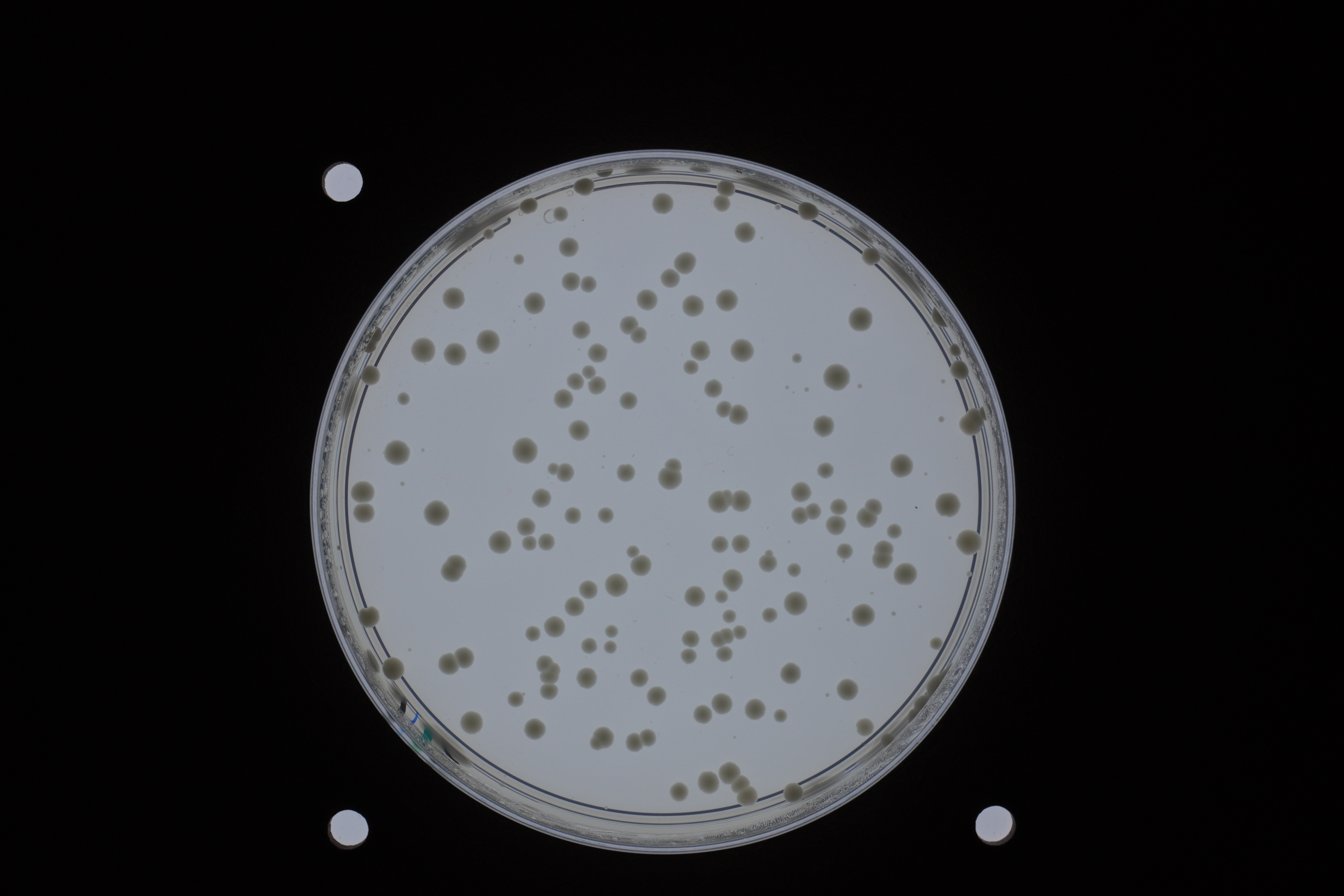

Supplement: Supplementary file 19 — Figure EV1 Source Data [file 44319_2026_702_MOESM19_ESM.zip › Figure EV1_SourceData/EV1A/Images/No fluconazole_H2O_Control_SCmURA_7.TIFF]

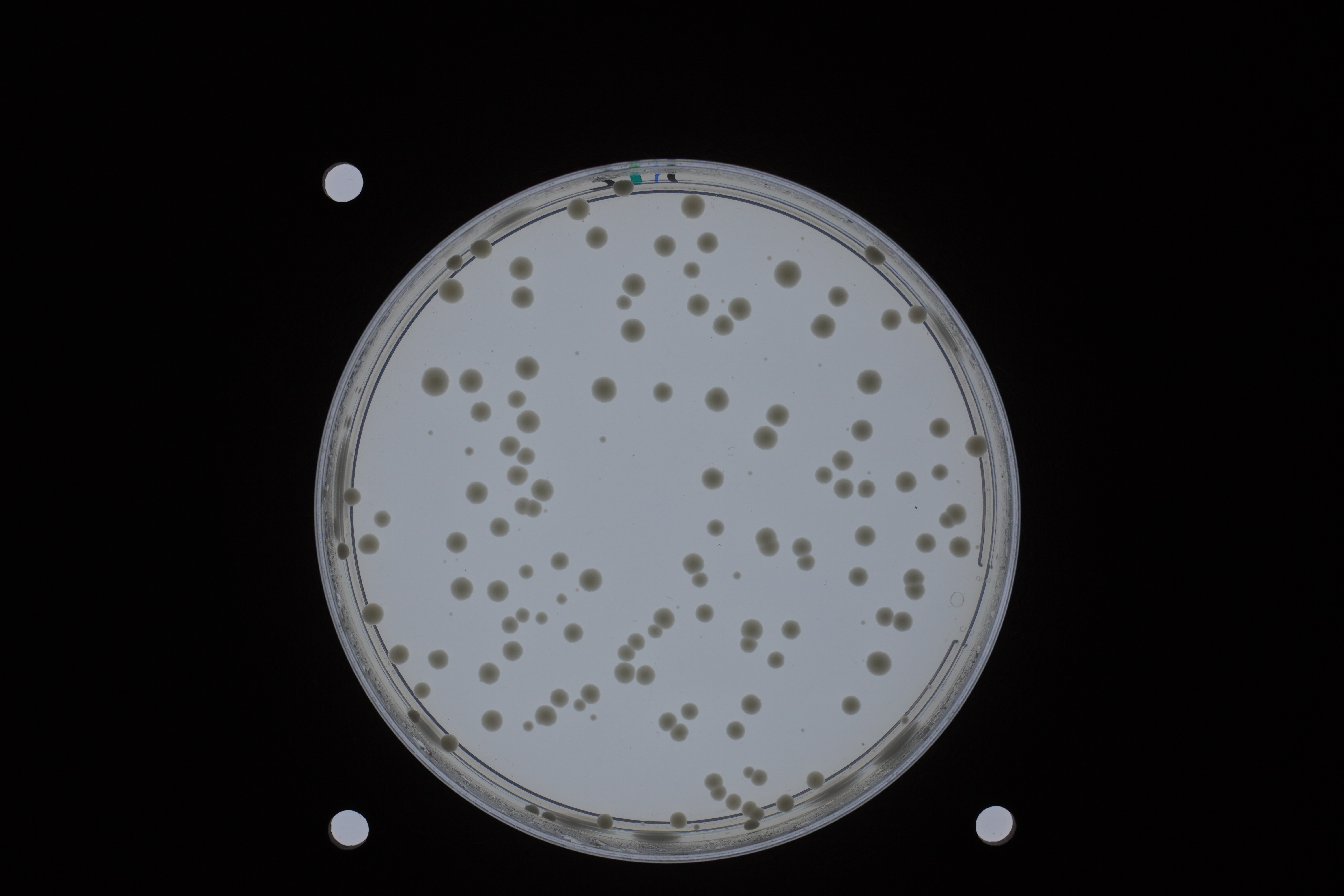

Supplement: Supplementary file 19 — Figure EV1 Source Data [file 44319_2026_702_MOESM19_ESM.zip › Figure EV1_SourceData/EV1A/Images/No fluconazole_H2O_Control_SCmURA_8..TIFF]

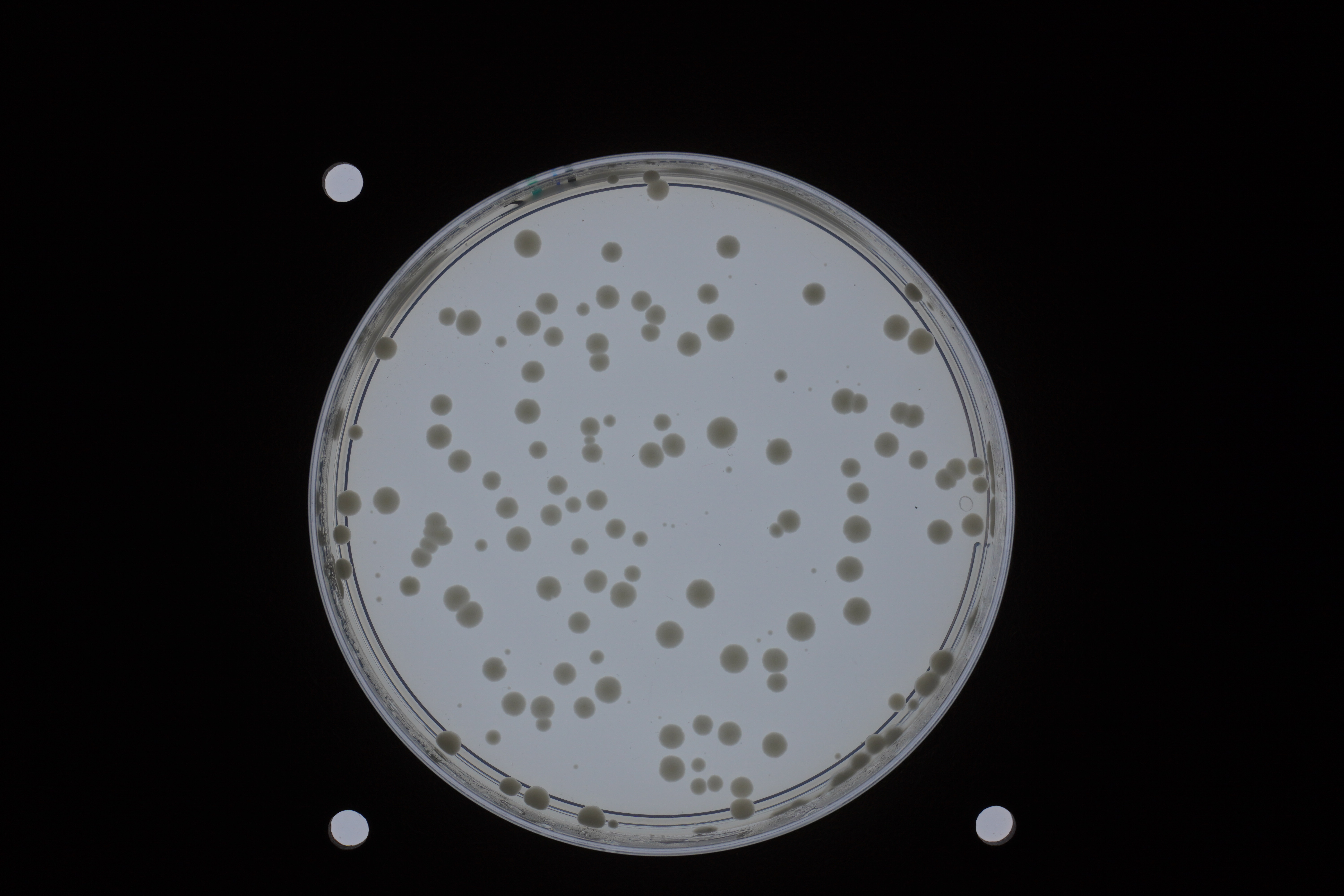

Supplement: Supplementary file 19 — Figure EV1 Source Data [file 44319_2026_702_MOESM19_ESM.zip › Figure EV1_SourceData/EV1A/Images/No fluconazole_H2O_Control_SCmURA_9.TIFF]

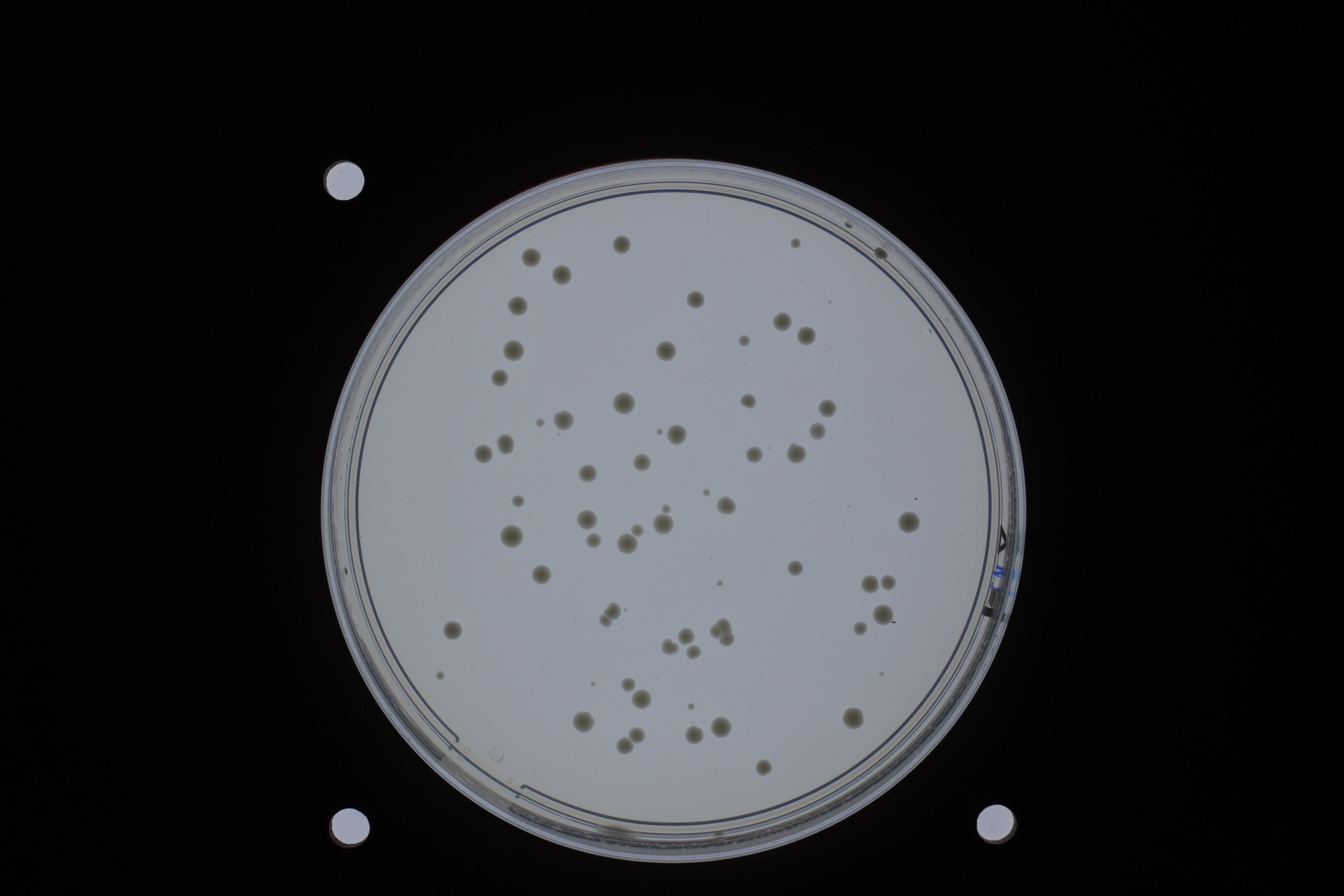

Supplement: Supplementary file 19 — Figure EV1 Source Data [file 44319_2026_702_MOESM19_ESM.zip › Figure EV1_SourceData/EV1A/Images/No fluconazole_H2O_Deletion_5FOA_1.TIFF]

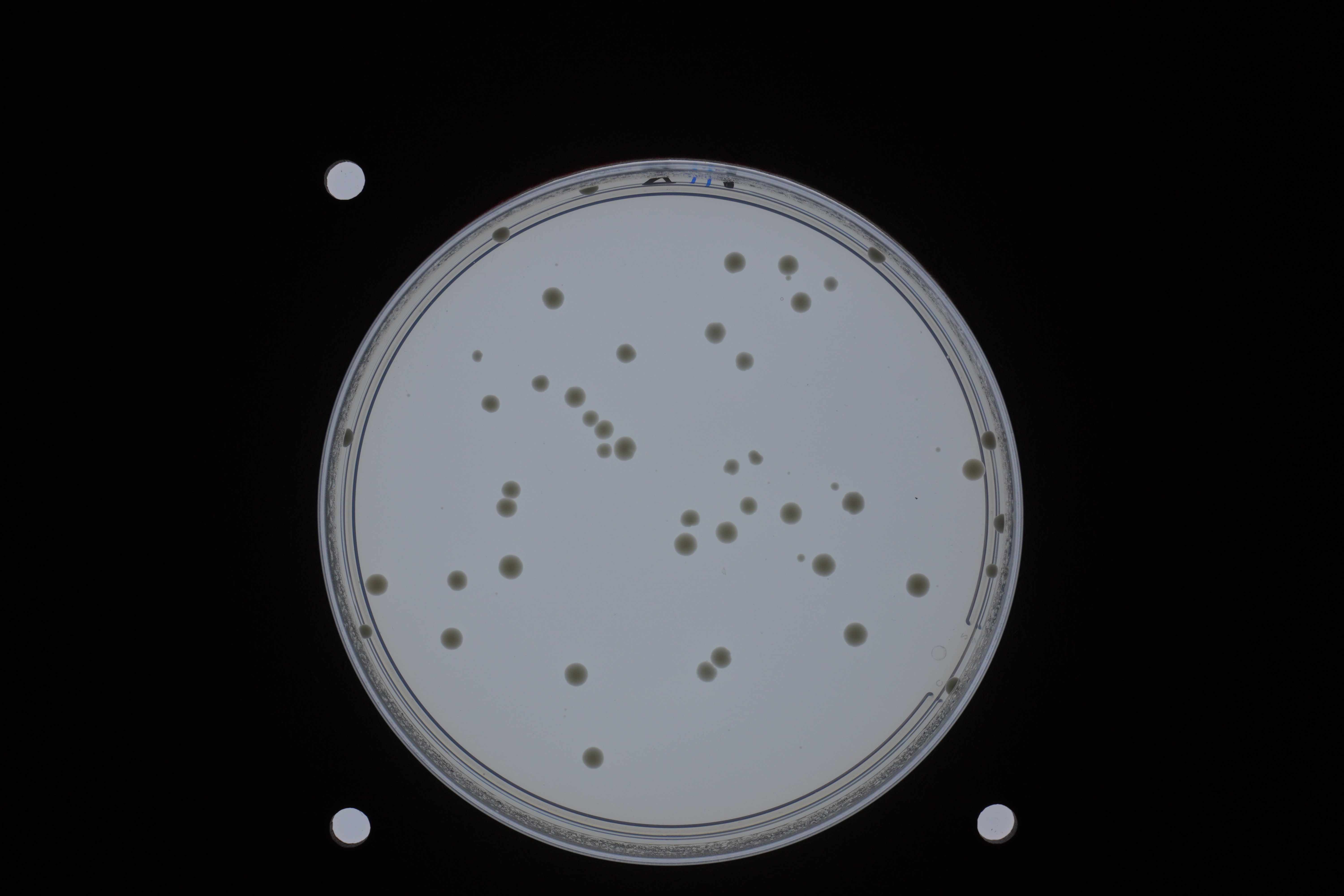

Supplement: Supplementary file 19 — Figure EV1 Source Data [file 44319_2026_702_MOESM19_ESM.zip › Figure EV1_SourceData/EV1A/Images/No fluconazole_H2O_Deletion_5FOA_10.TIFF]

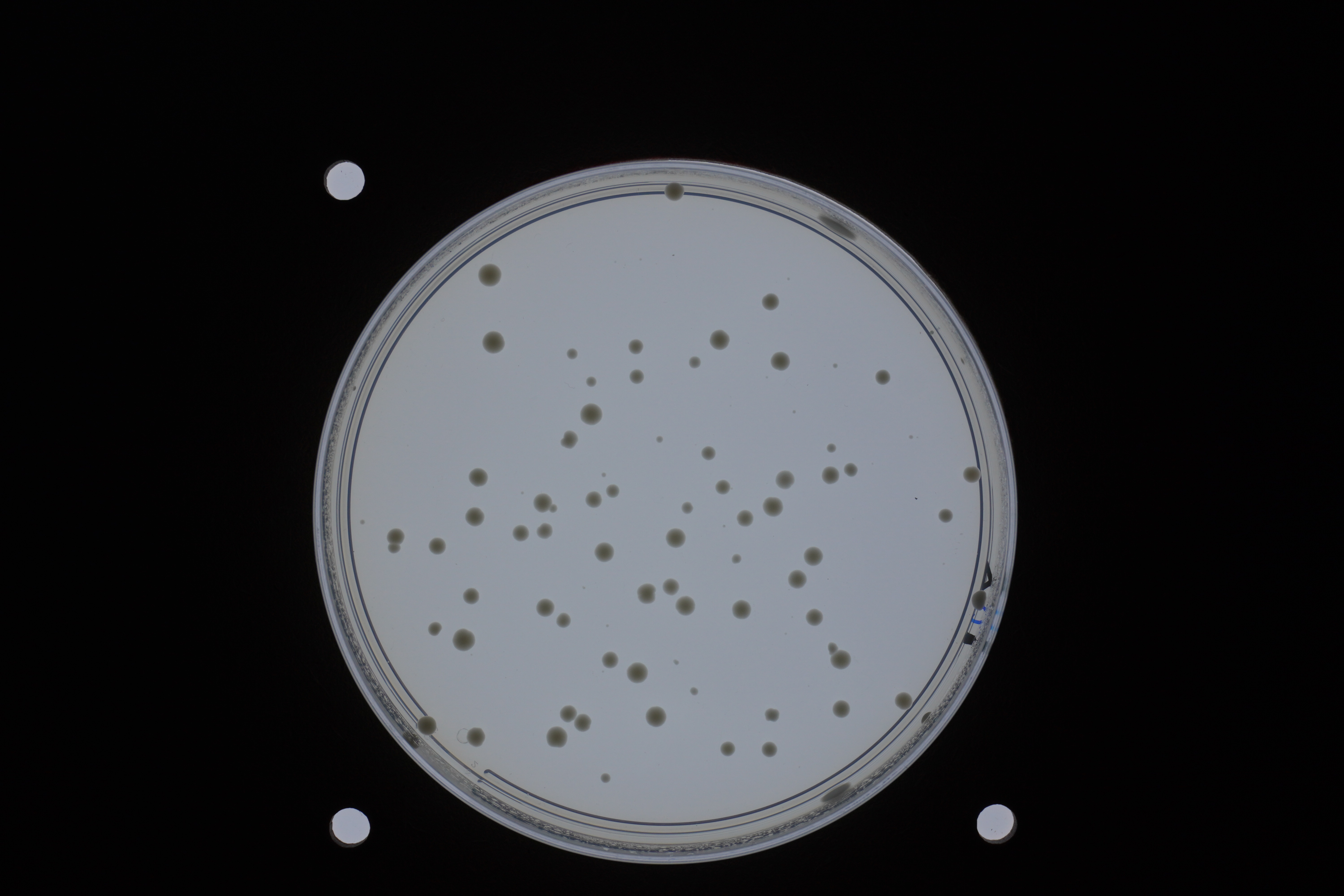

Supplement: Supplementary file 19 — Figure EV1 Source Data [file 44319_2026_702_MOESM19_ESM.zip › Figure EV1_SourceData/EV1A/Images/No fluconazole_H2O_Deletion_5FOA_2.TIFF]

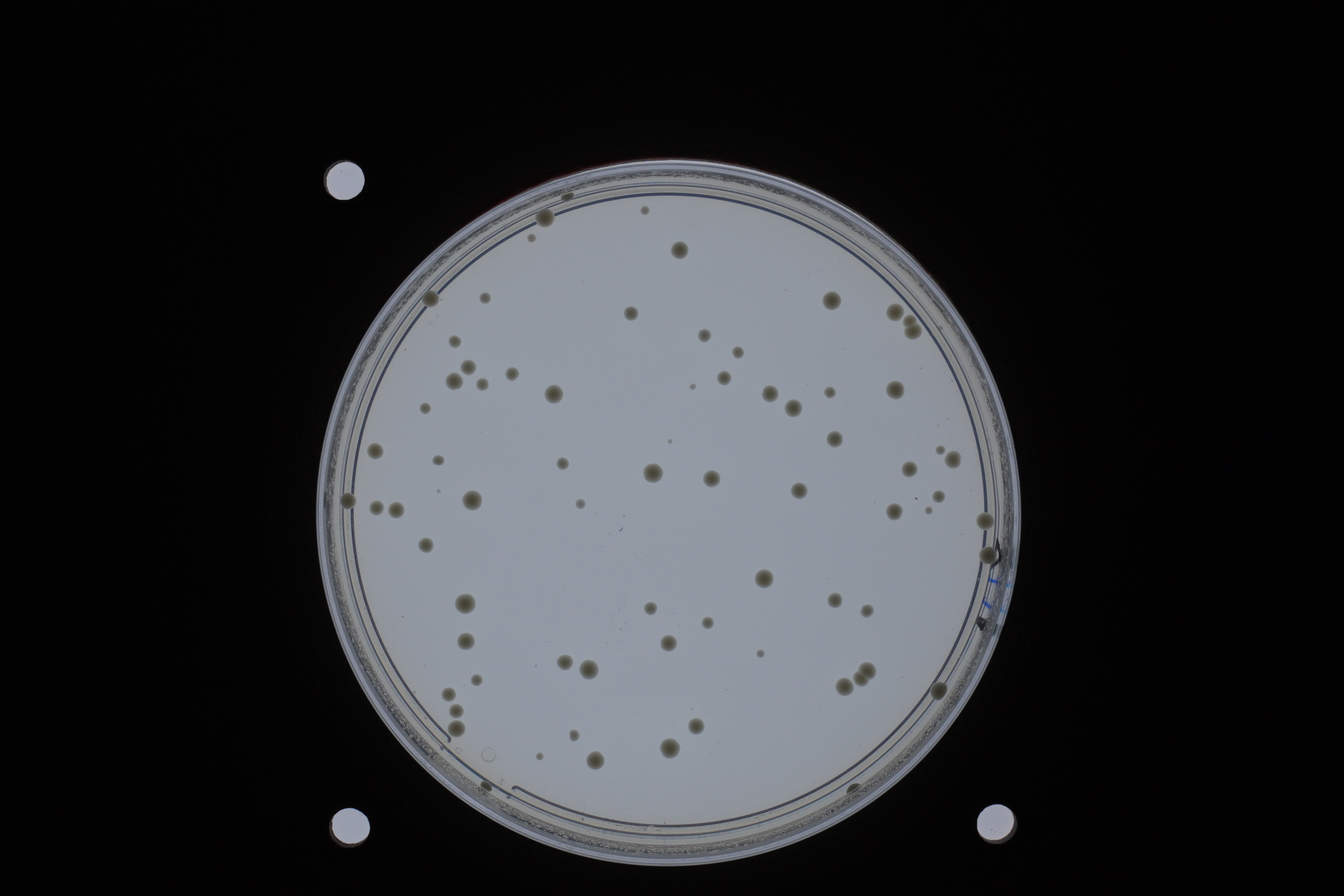

Supplement: Supplementary file 19 — Figure EV1 Source Data [file 44319_2026_702_MOESM19_ESM.zip › Figure EV1_SourceData/EV1A/Images/No fluconazole_H2O_Deletion_5FOA_3.TIFF]

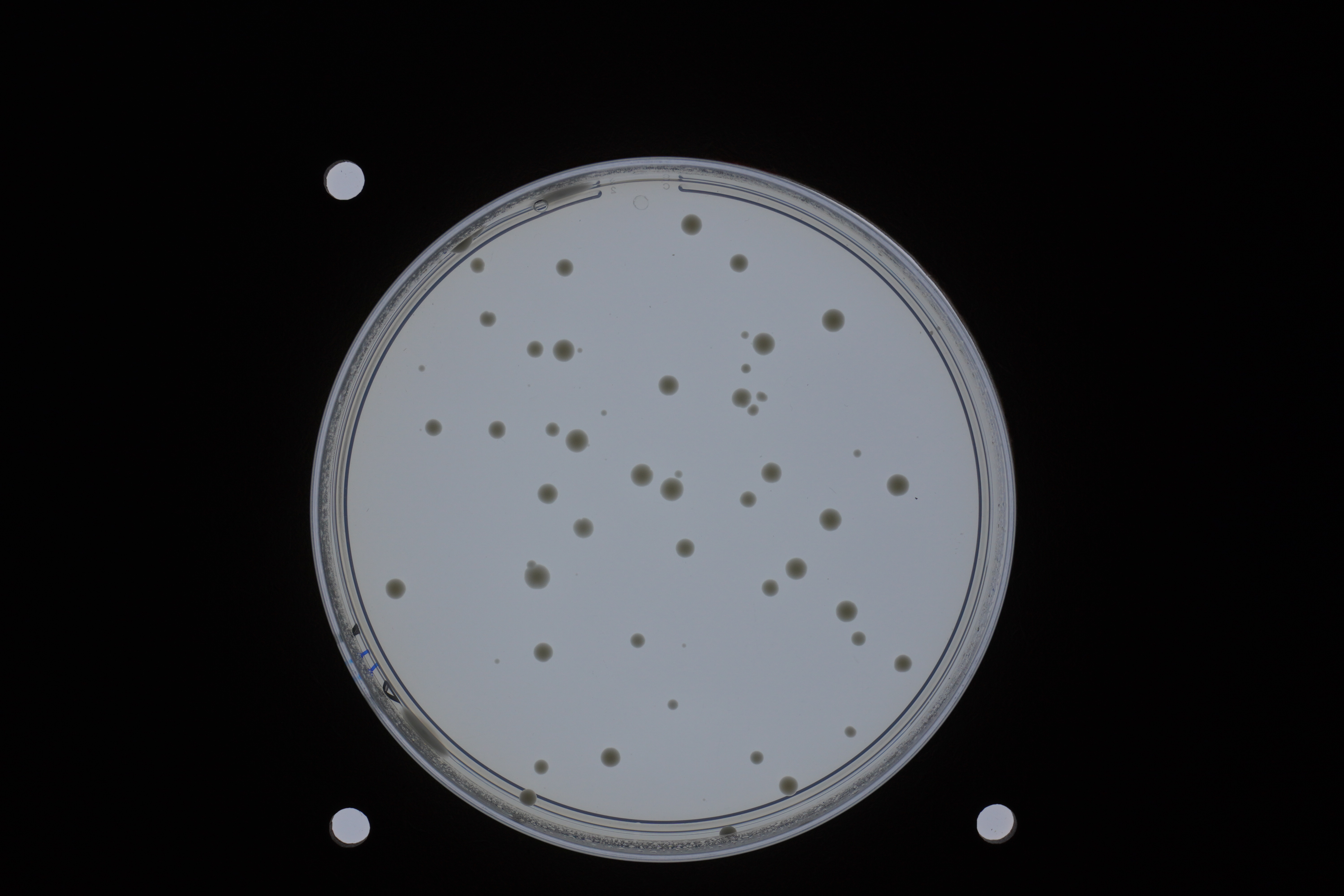

Supplement: Supplementary file 19 — Figure EV1 Source Data [file 44319_2026_702_MOESM19_ESM.zip › Figure EV1_SourceData/EV1A/Images/No fluconazole_H2O_Deletion_5FOA_4.TIFF]

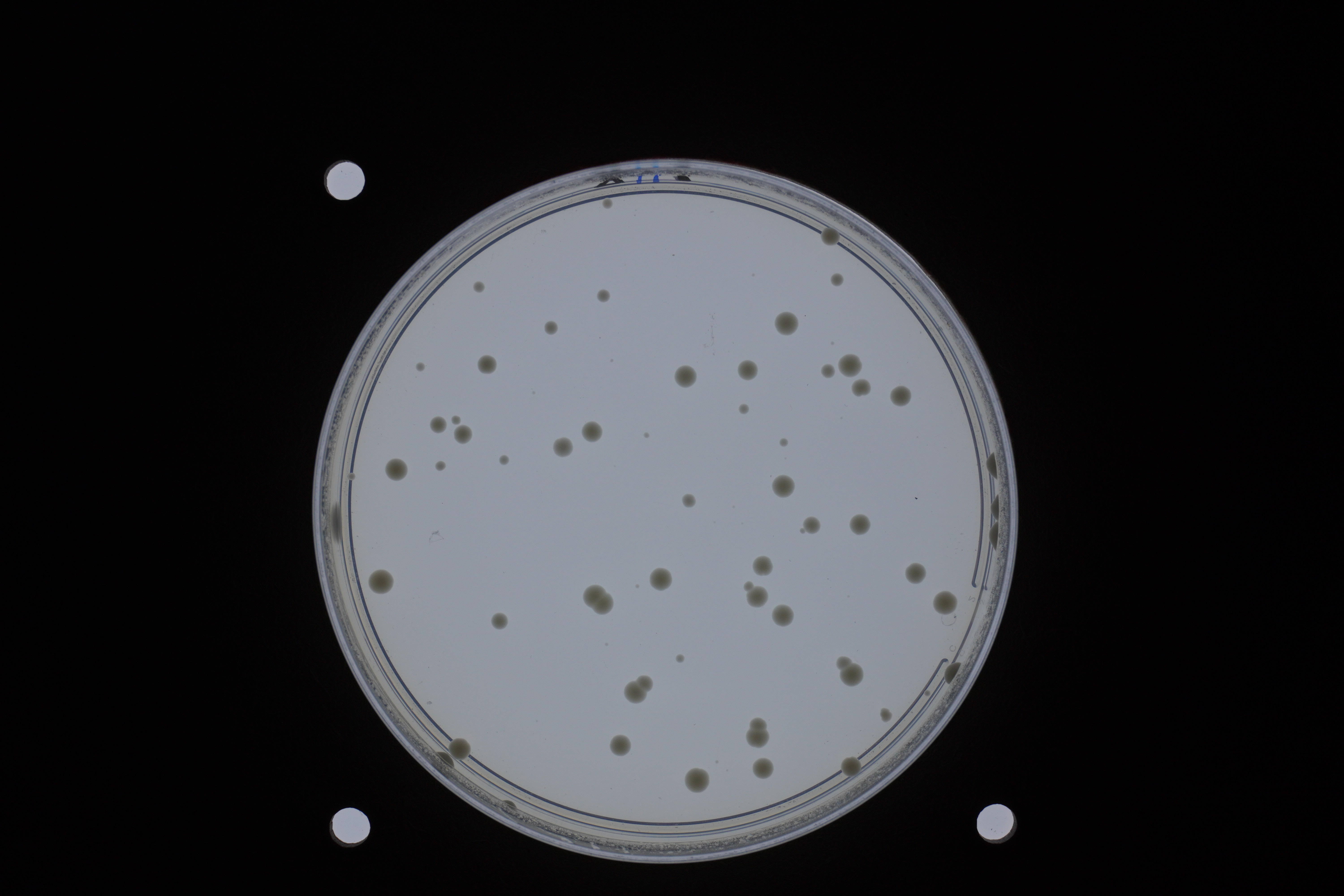

Supplement: Supplementary file 19 — Figure EV1 Source Data [file 44319_2026_702_MOESM19_ESM.zip › Figure EV1_SourceData/EV1A/Images/No fluconazole_H2O_Deletion_5FOA_5.TIFF]

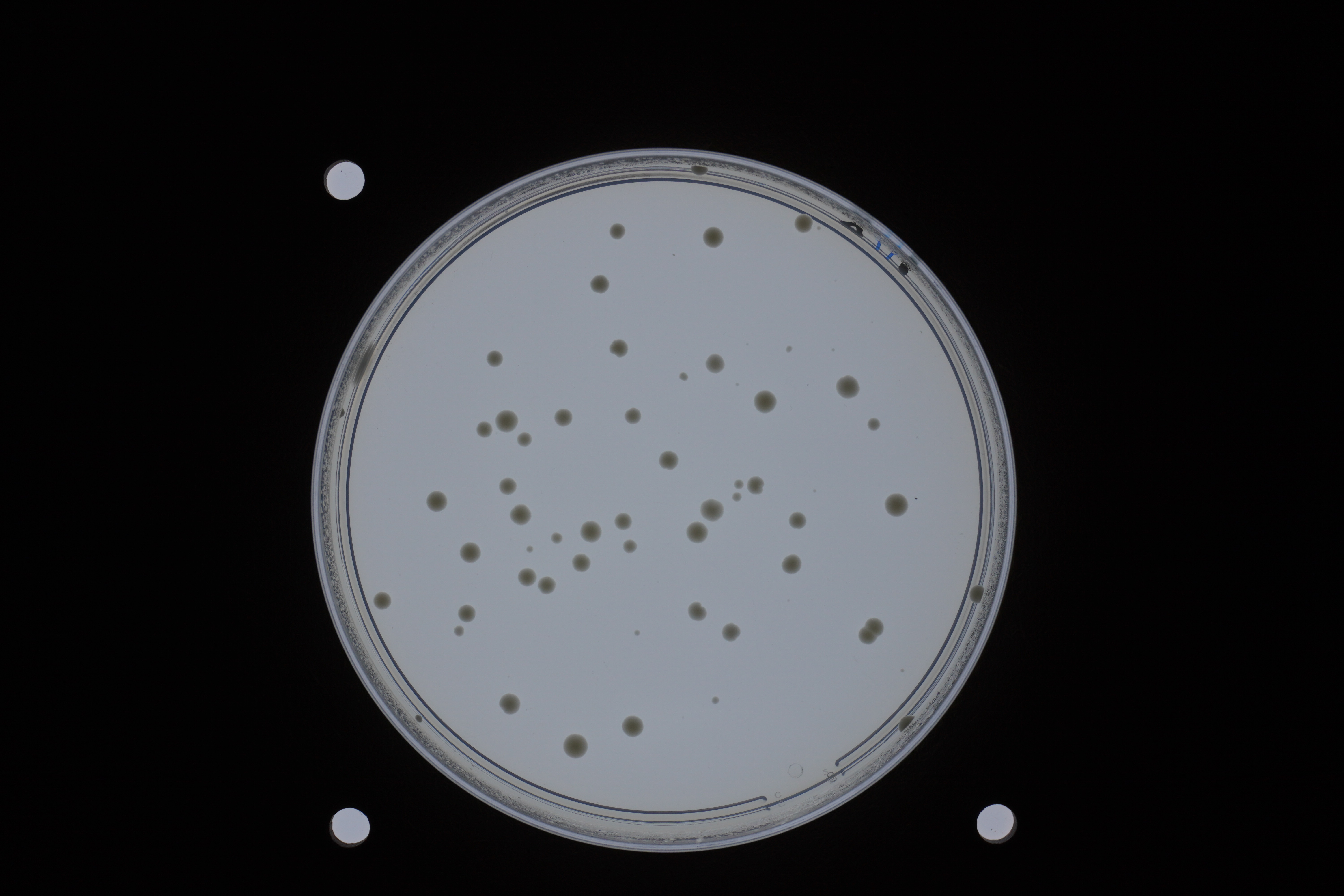

Supplement: Supplementary file 19 — Figure EV1 Source Data [file 44319_2026_702_MOESM19_ESM.zip › Figure EV1_SourceData/EV1A/Images/No fluconazole_H2O_Deletion_5FOA_6.TIFF]

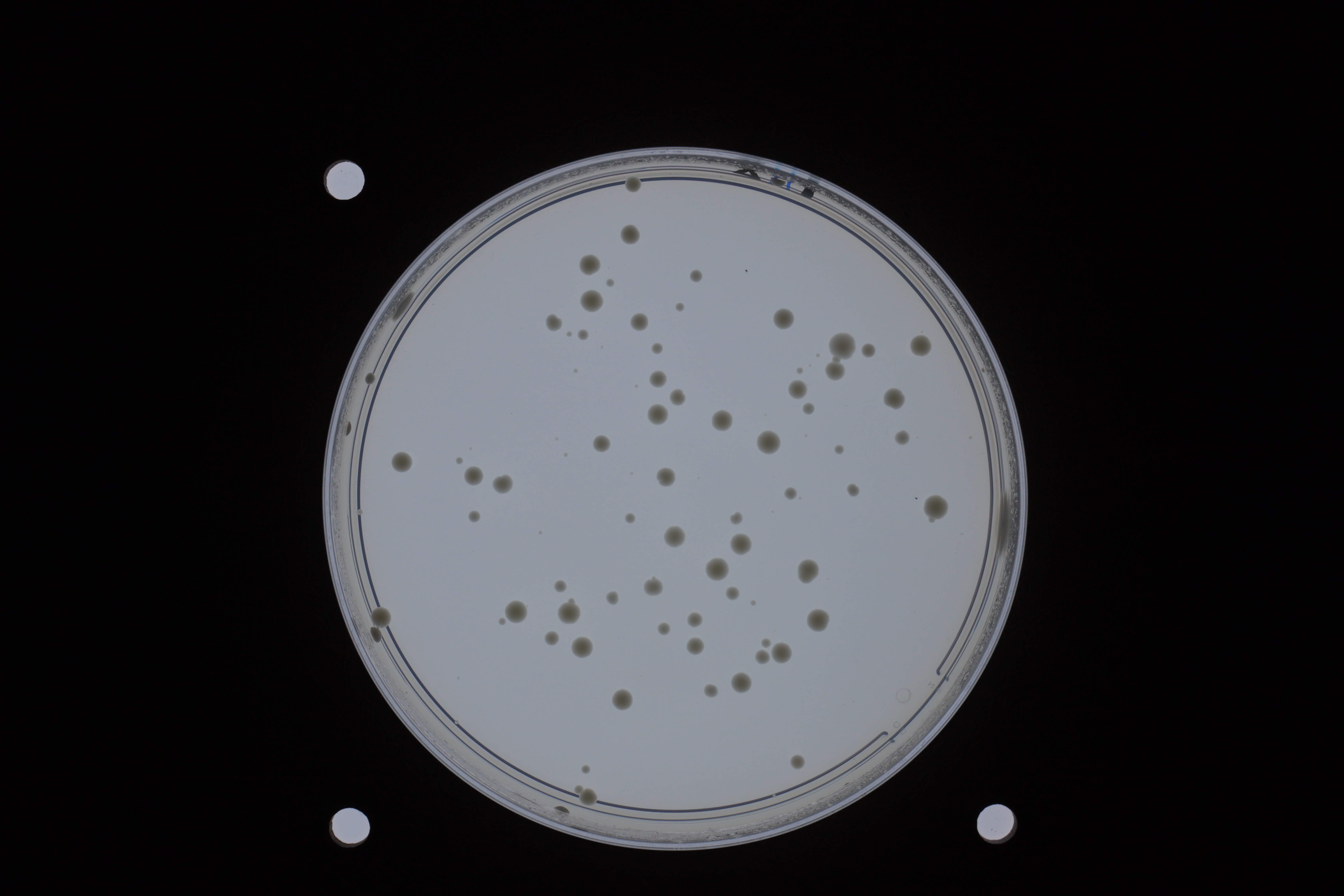

Supplement: Supplementary file 19 — Figure EV1 Source Data [file 44319_2026_702_MOESM19_ESM.zip › Figure EV1_SourceData/EV1A/Images/No fluconazole_H2O_Deletion_5FOA_7.TIFF]

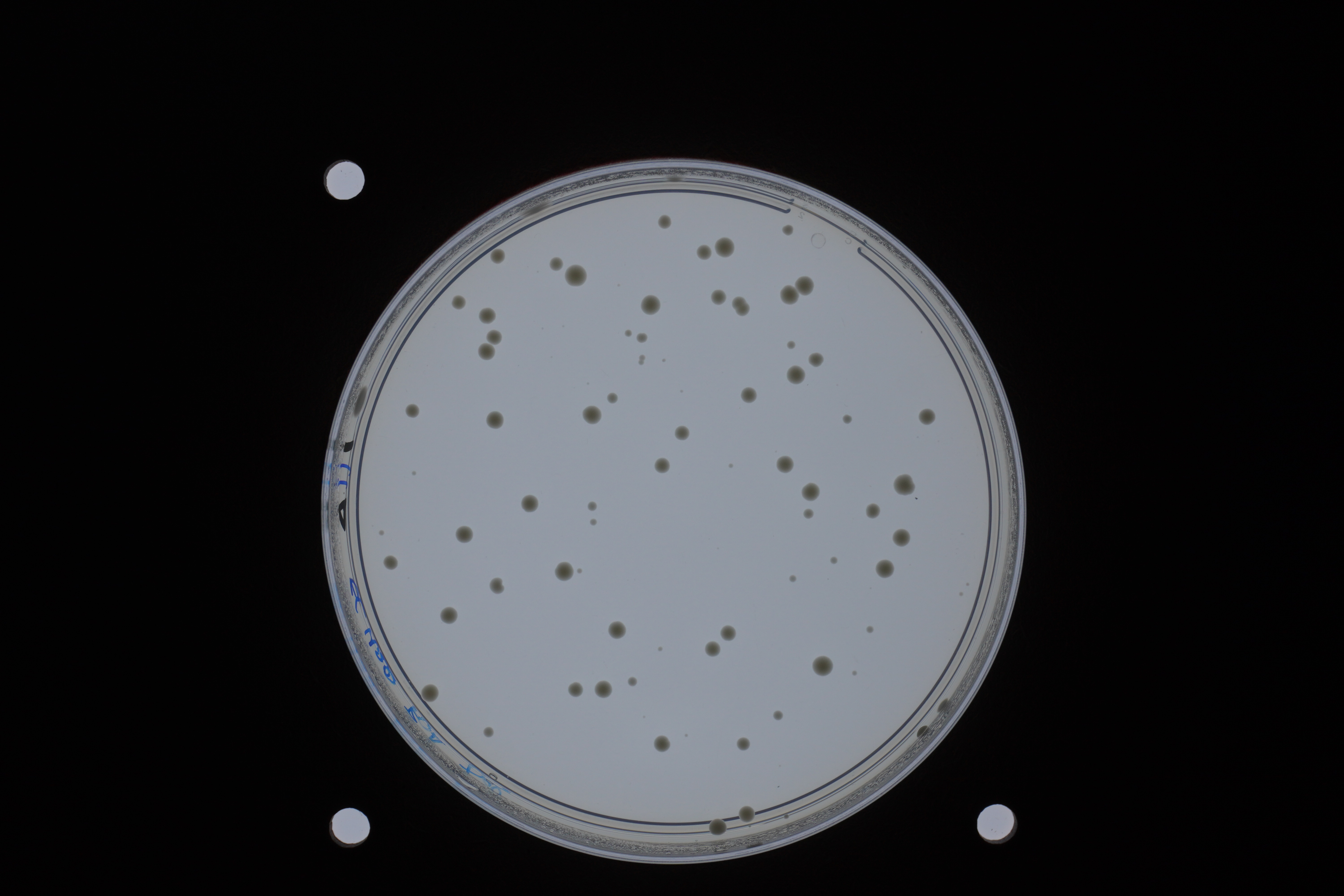

Supplement: Supplementary file 19 — Figure EV1 Source Data [file 44319_2026_702_MOESM19_ESM.zip › Figure EV1_SourceData/EV1A/Images/No fluconazole_H2O_Deletion_5FOA_8.TIFF]

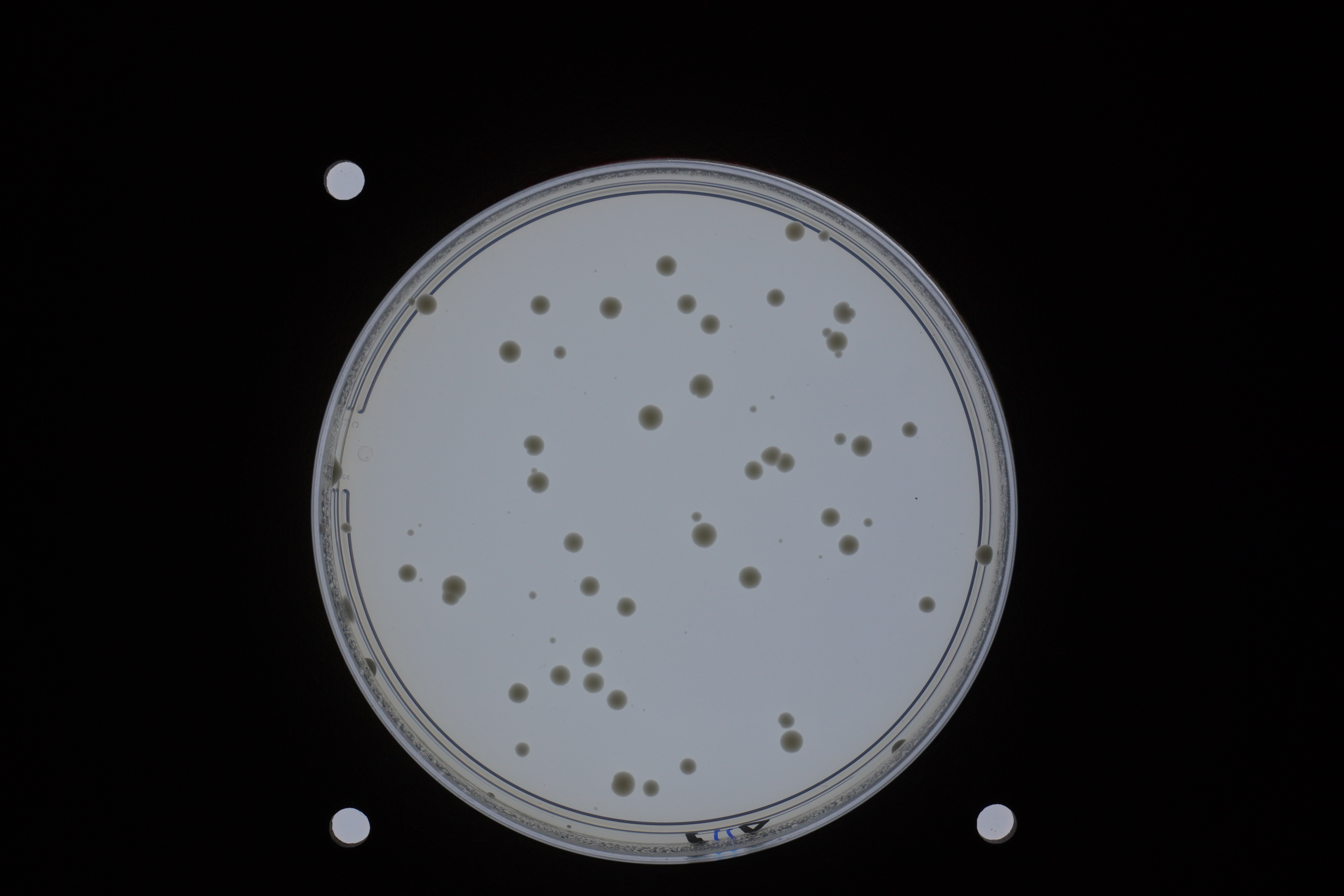

Supplement: Supplementary file 19 — Figure EV1 Source Data [file 44319_2026_702_MOESM19_ESM.zip › Figure EV1_SourceData/EV1A/Images/No fluconazole_H2O_Deletion_5FOA_9.TIFF]

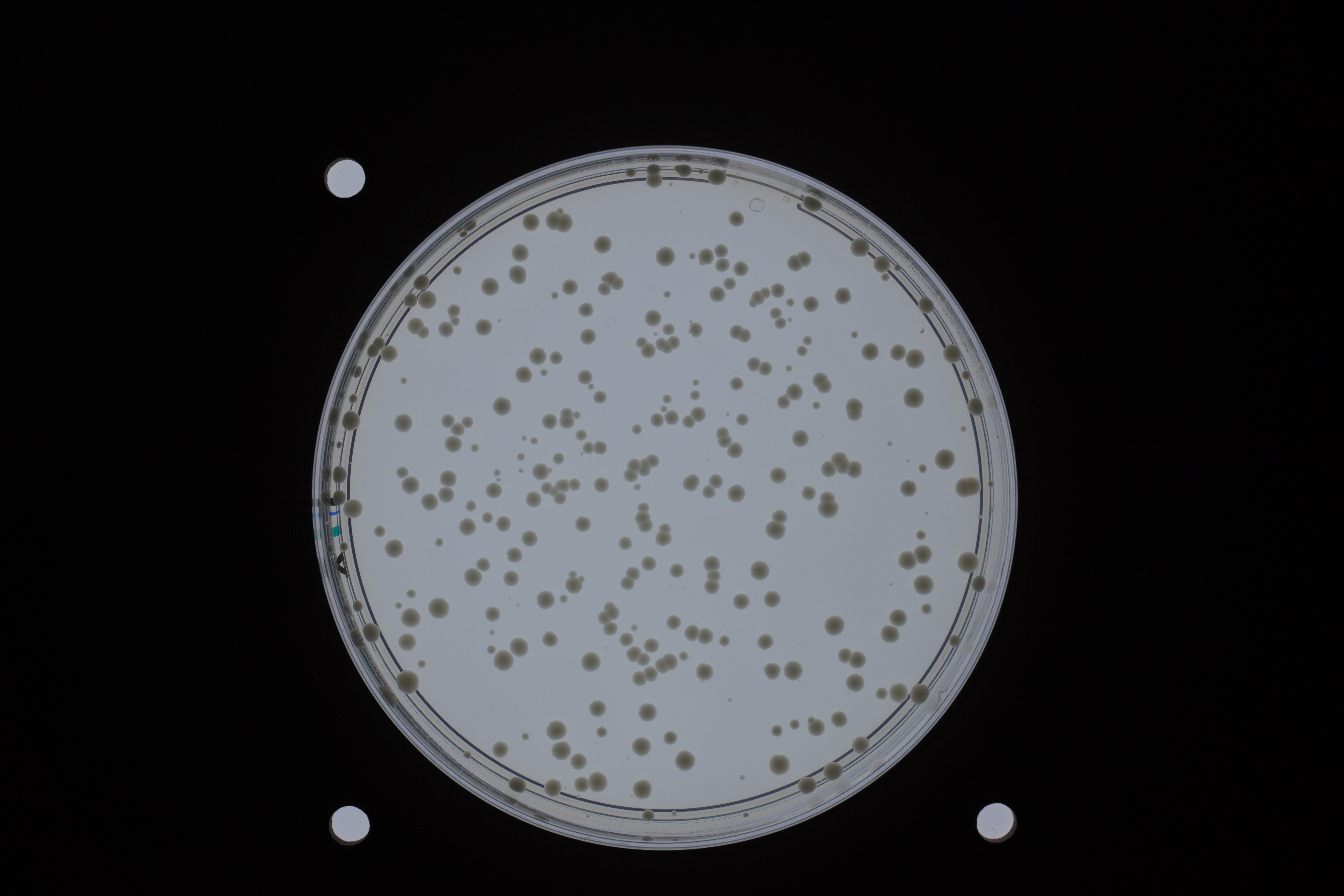

Supplement: Supplementary file 19 — Figure EV1 Source Data [file 44319_2026_702_MOESM19_ESM.zip › Figure EV1_SourceData/EV1A/Images/No fluconazole_H2O_Deletion_SCmURA_1.TIFF]

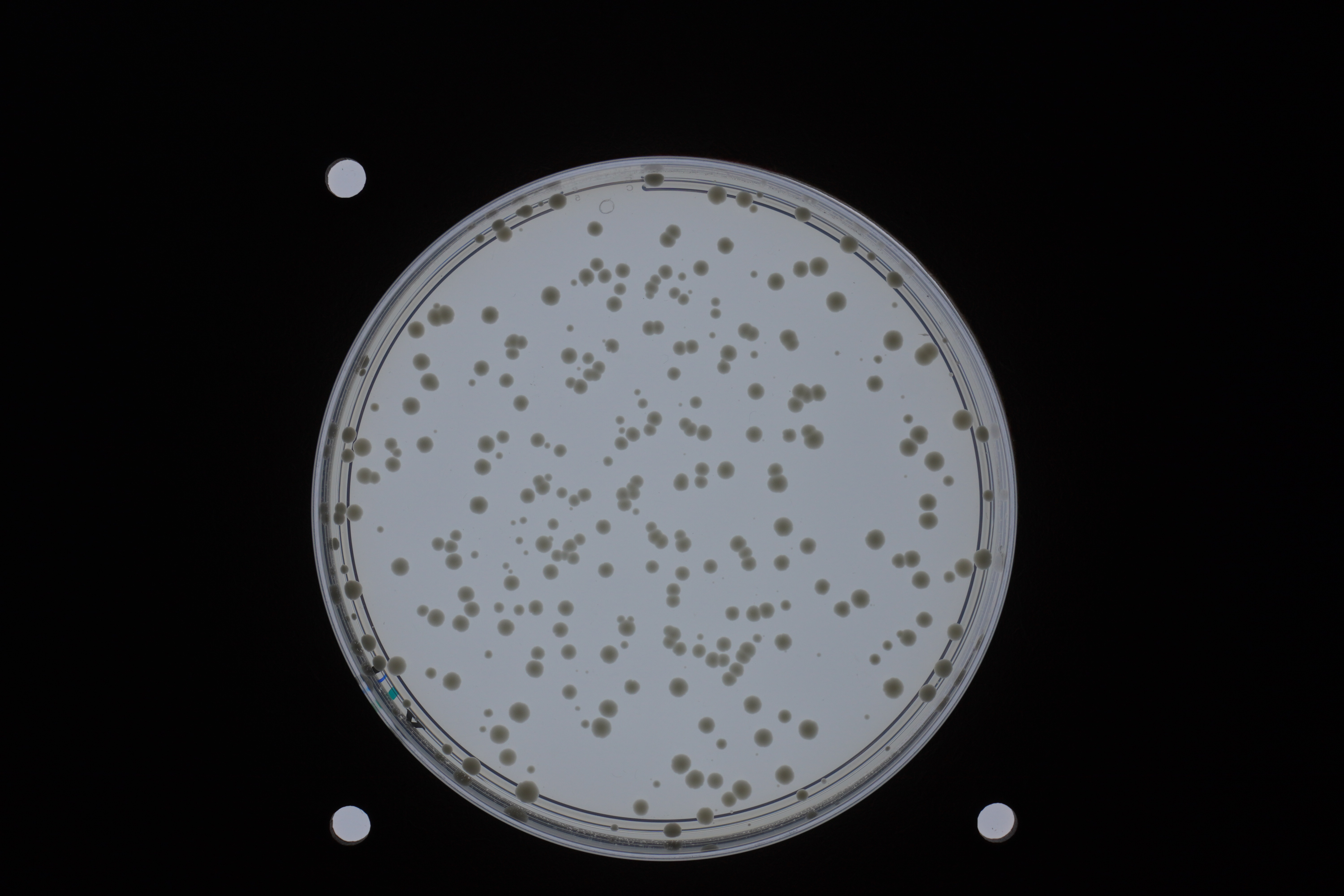

Supplement: Supplementary file 19 — Figure EV1 Source Data [file 44319_2026_702_MOESM19_ESM.zip › Figure EV1_SourceData/EV1A/Images/No fluconazole_H2O_Deletion_SCmURA_10.TIFF]

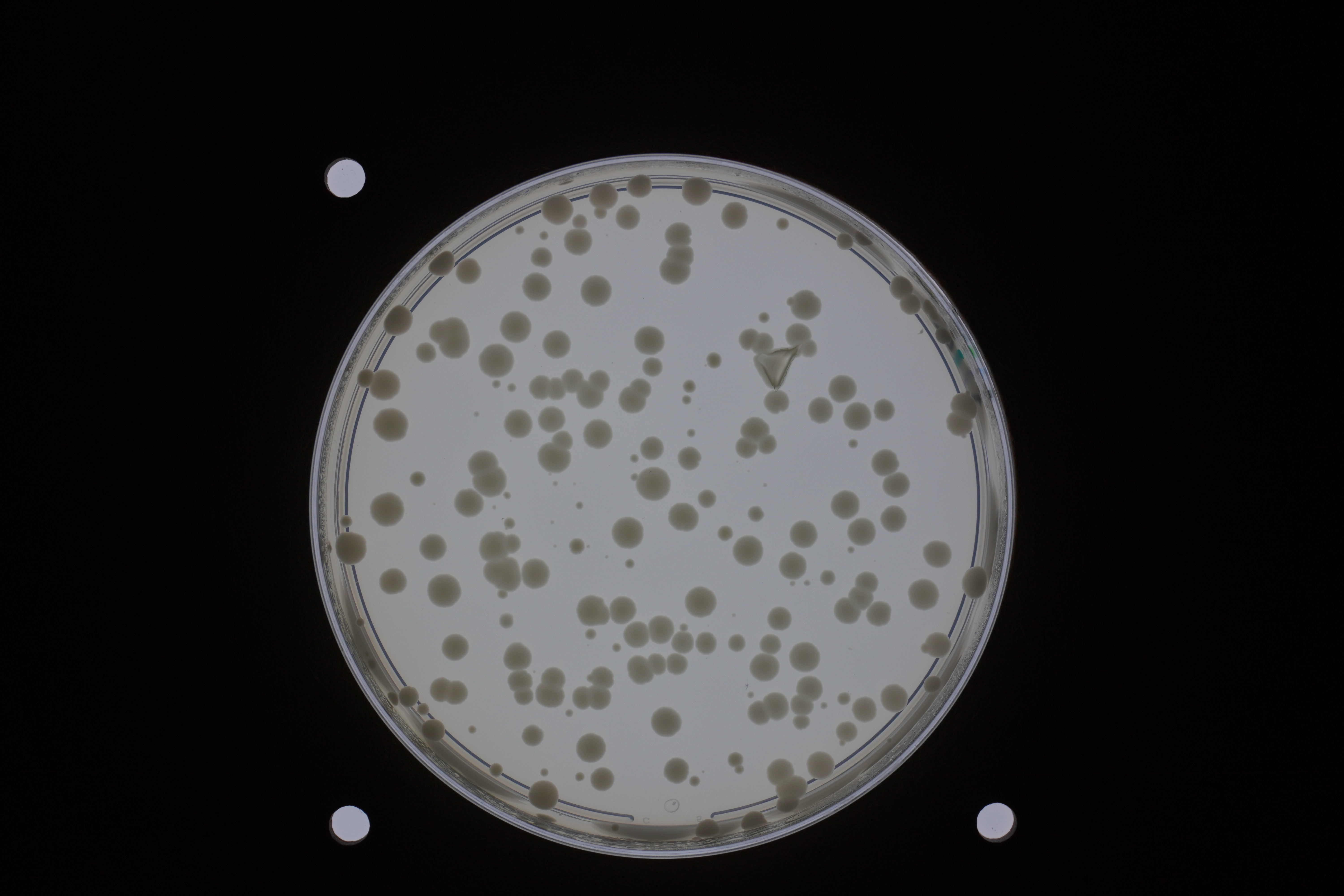

Supplement: Supplementary file 19 — Figure EV1 Source Data [file 44319_2026_702_MOESM19_ESM.zip › Figure EV1_SourceData/EV1A/Images/No fluconazole_H2O_Deletion_SCmURA_2.TIFF]

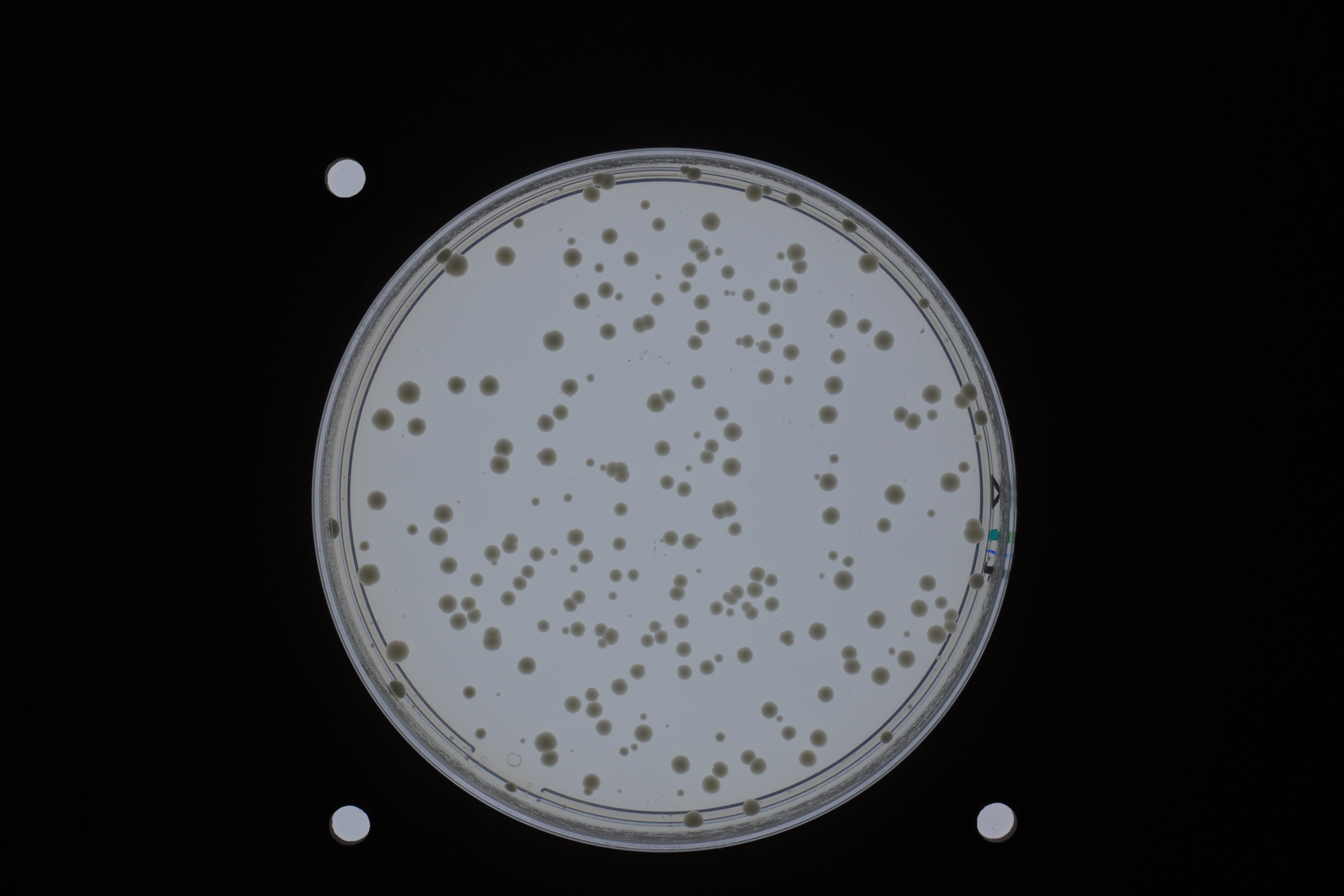

Supplement: Supplementary file 19 — Figure EV1 Source Data [file 44319_2026_702_MOESM19_ESM.zip › Figure EV1_SourceData/EV1A/Images/No fluconazole_H2O_Deletion_SCmURA_3.TIFF]

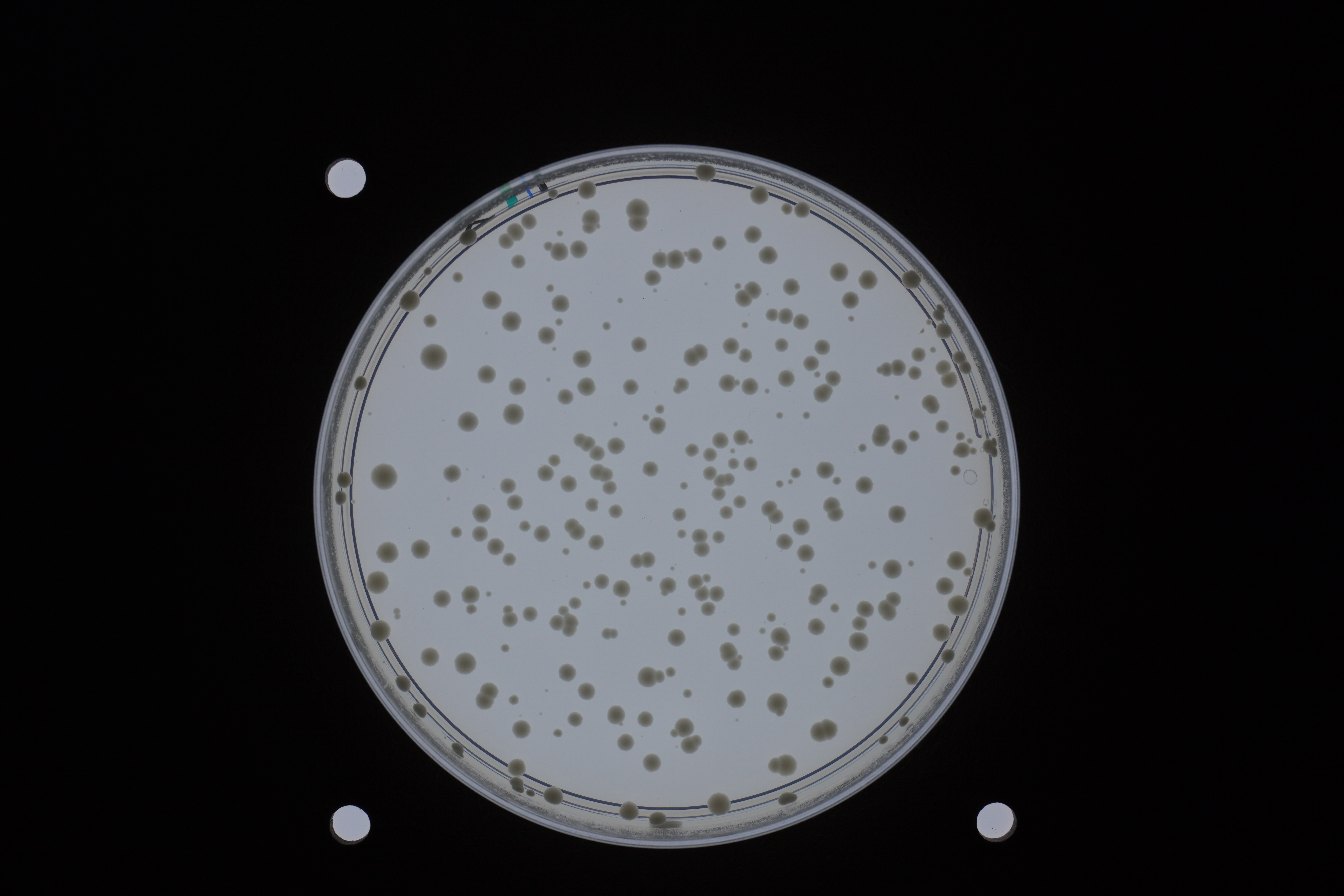

Supplement: Supplementary file 19 — Figure EV1 Source Data [file 44319_2026_702_MOESM19_ESM.zip › Figure EV1_SourceData/EV1A/Images/No fluconazole_H2O_Deletion_SCmURA_4.TIFF]

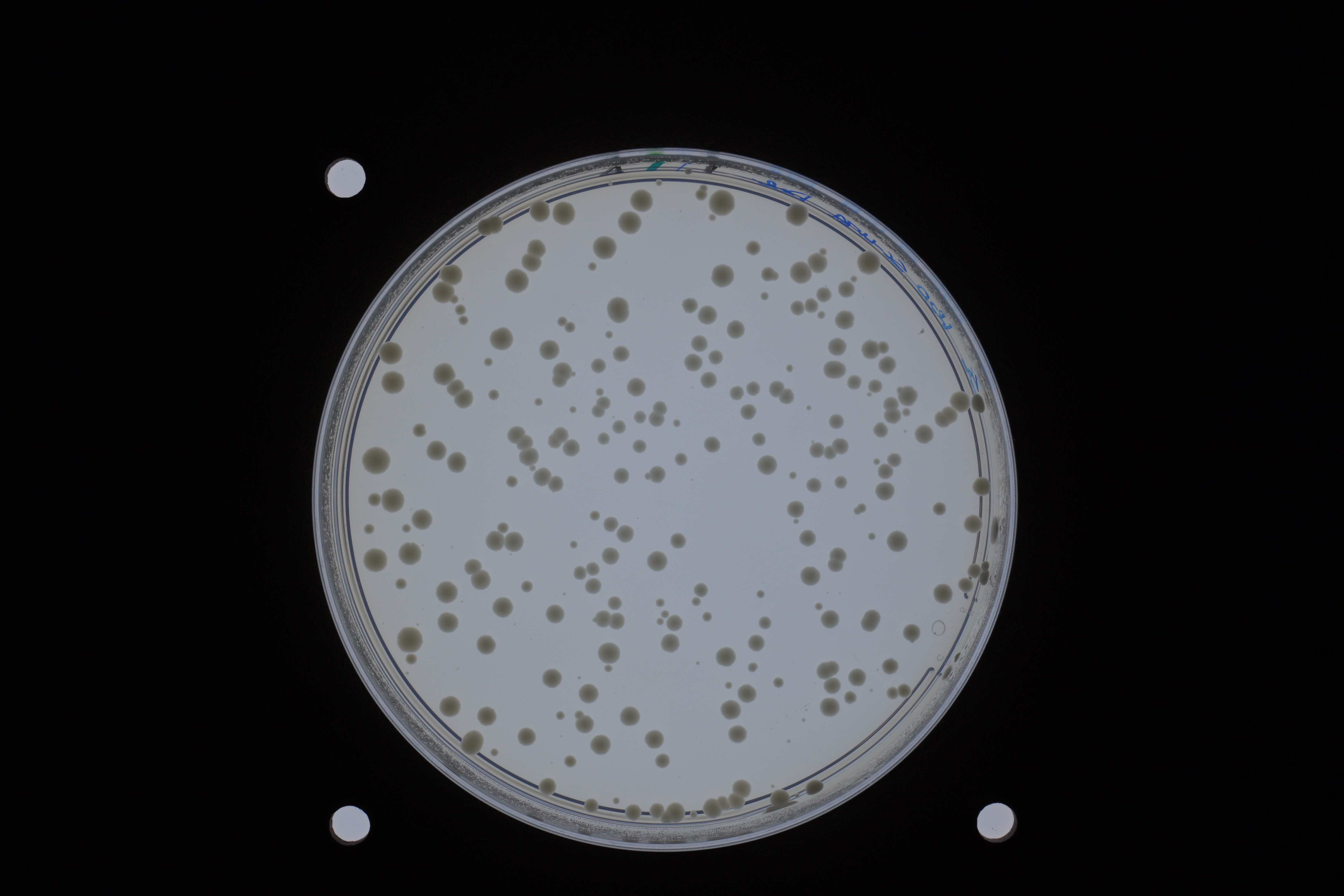

Supplement: Supplementary file 19 — Figure EV1 Source Data [file 44319_2026_702_MOESM19_ESM.zip › Figure EV1_SourceData/EV1A/Images/No fluconazole_H2O_Deletion_SCmURA_5.TIFF]

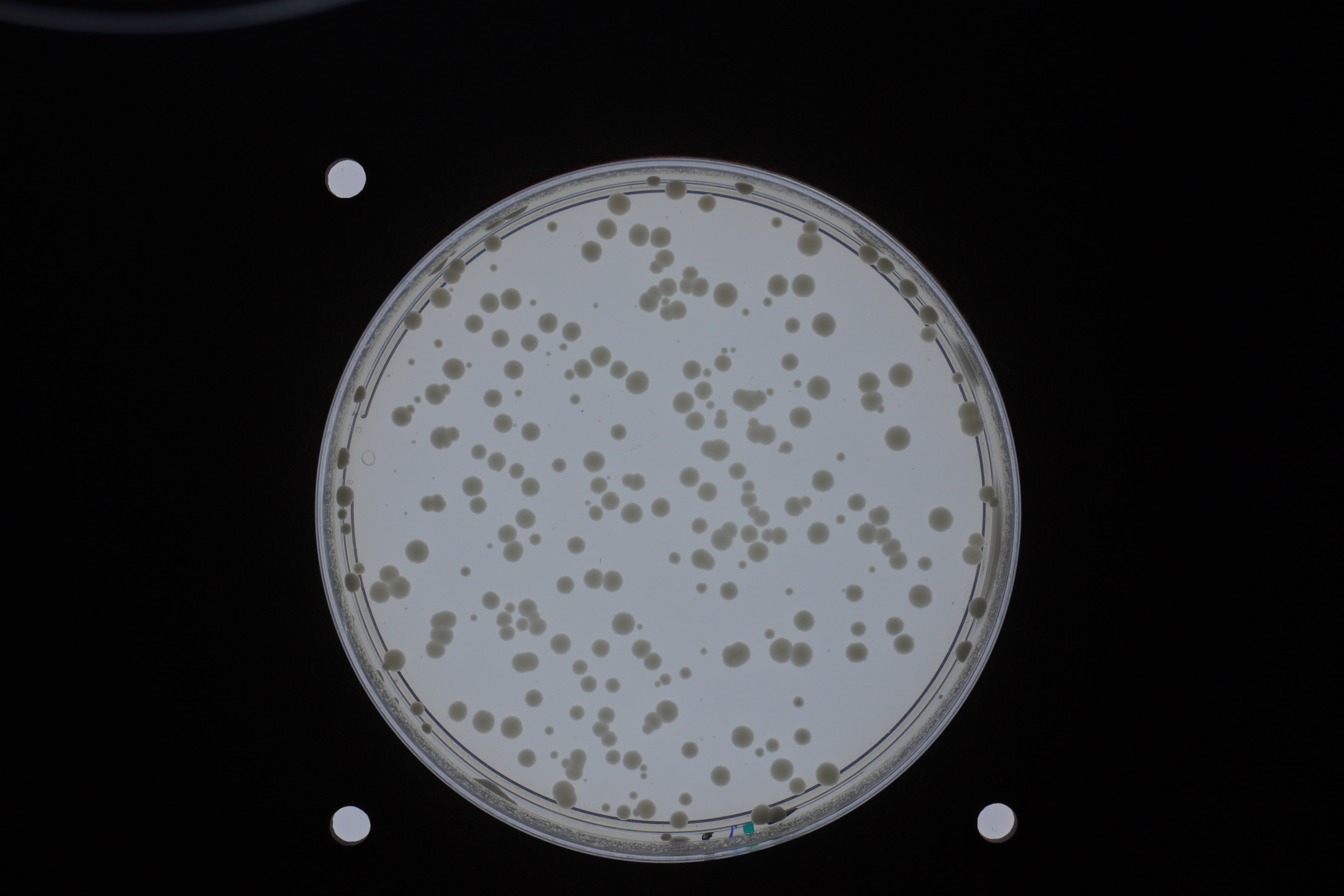

Supplement: Supplementary file 19 — Figure EV1 Source Data [file 44319_2026_702_MOESM19_ESM.zip › Figure EV1_SourceData/EV1A/Images/No fluconazole_H2O_Deletion_SCmURA_6.TIFF]

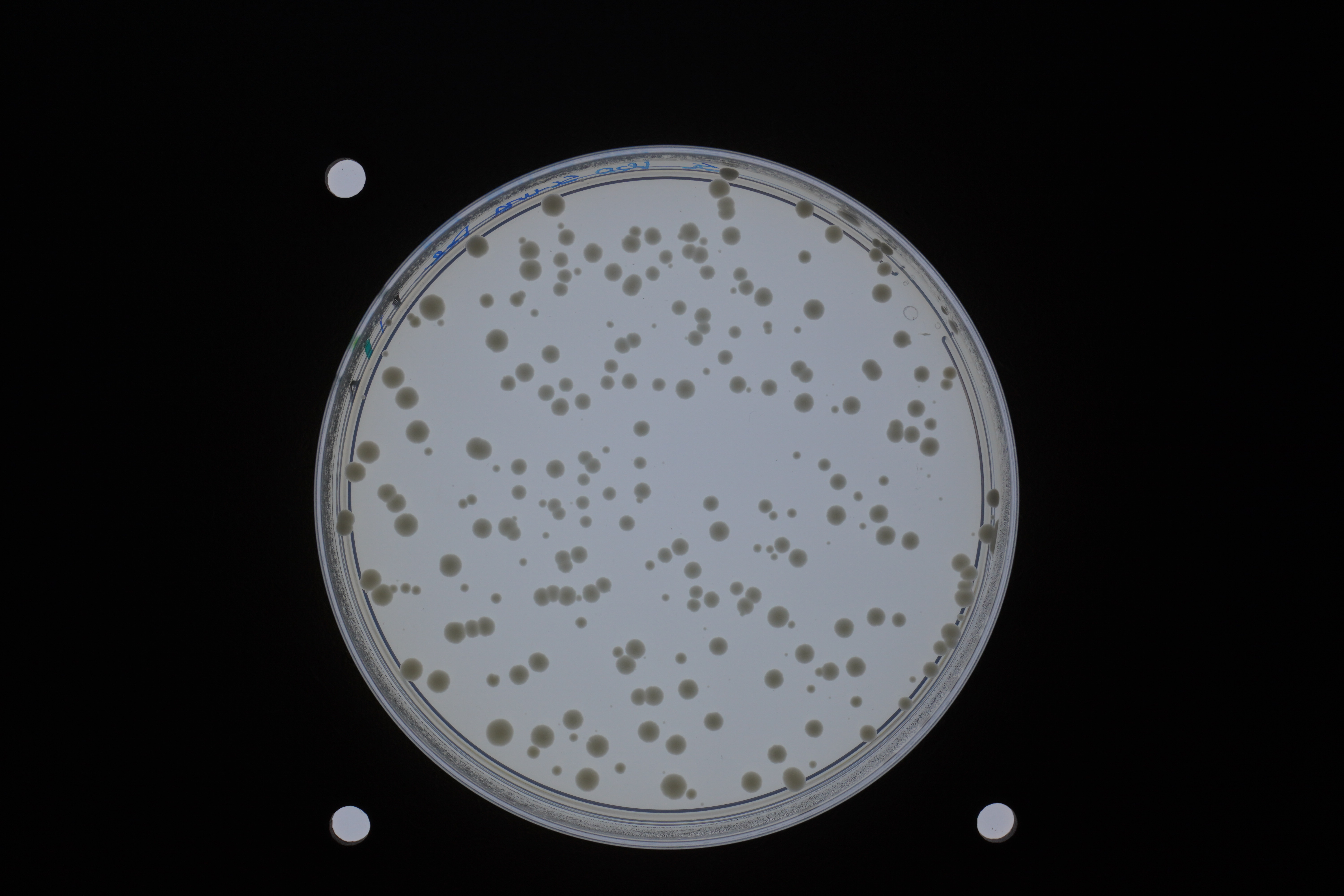

Supplement: Supplementary file 19 — Figure EV1 Source Data [file 44319_2026_702_MOESM19_ESM.zip › Figure EV1_SourceData/EV1A/Images/No fluconazole_H2O_Deletion_SCmURA_7.TIFF]

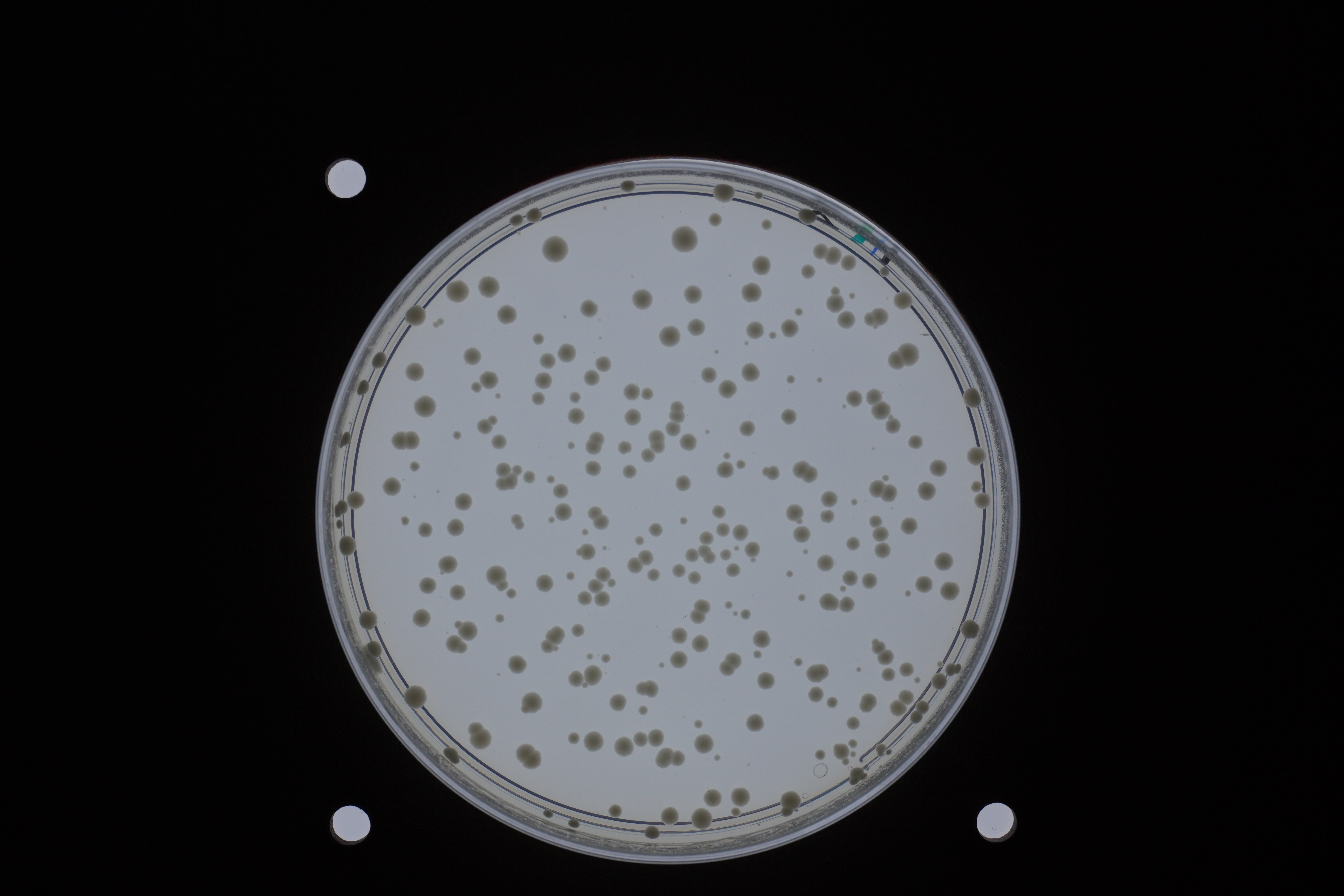

Supplement: Supplementary file 19 — Figure EV1 Source Data [file 44319_2026_702_MOESM19_ESM.zip › Figure EV1_SourceData/EV1A/Images/No fluconazole_H2O_Deletion_SCmURA_8.TIFF]

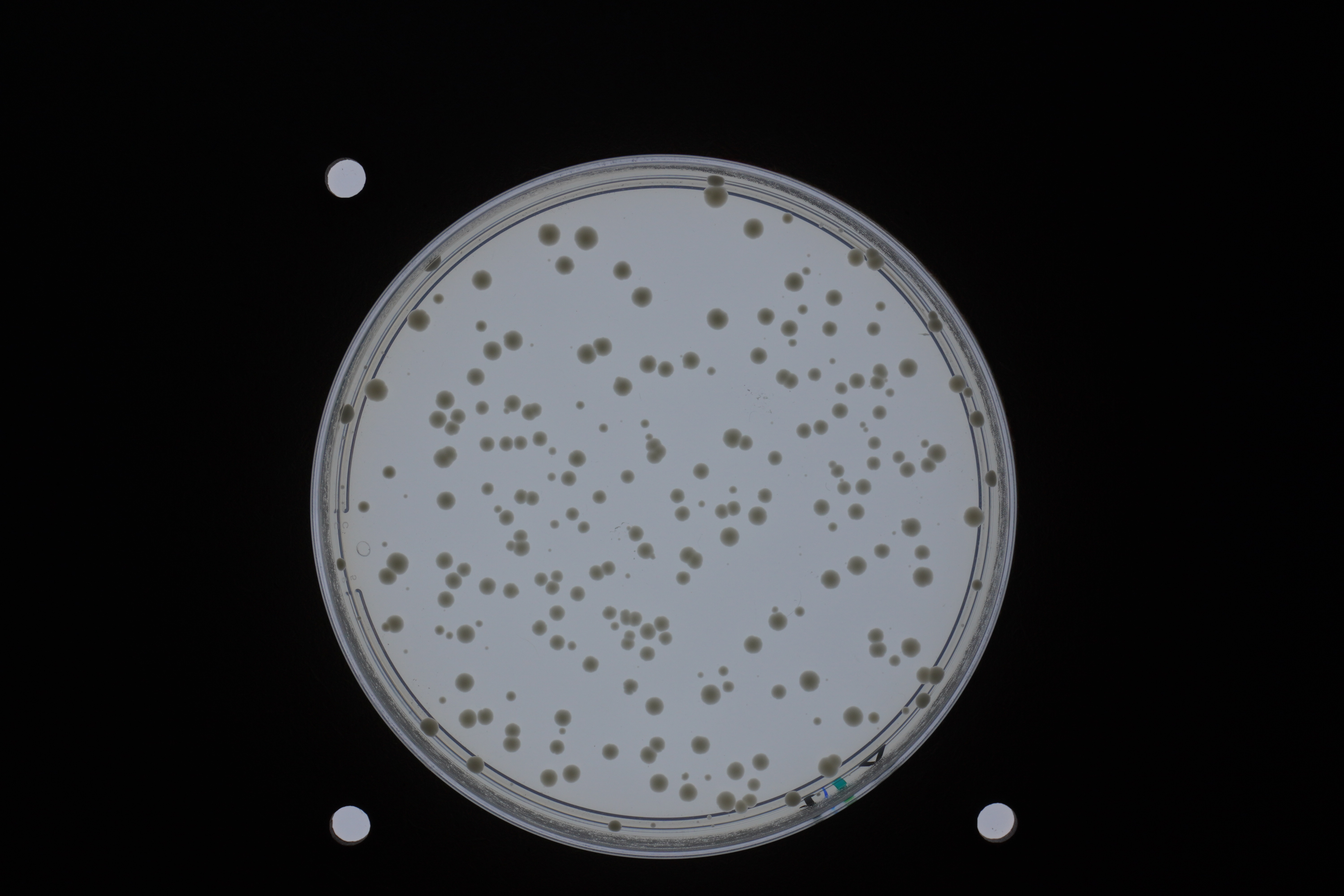

Supplement: Supplementary file 19 — Figure EV1 Source Data [file 44319_2026_702_MOESM19_ESM.zip › Figure EV1_SourceData/EV1A/Images/No fluconazole_H2O_Deletion_SCmURA_9.TIFF]

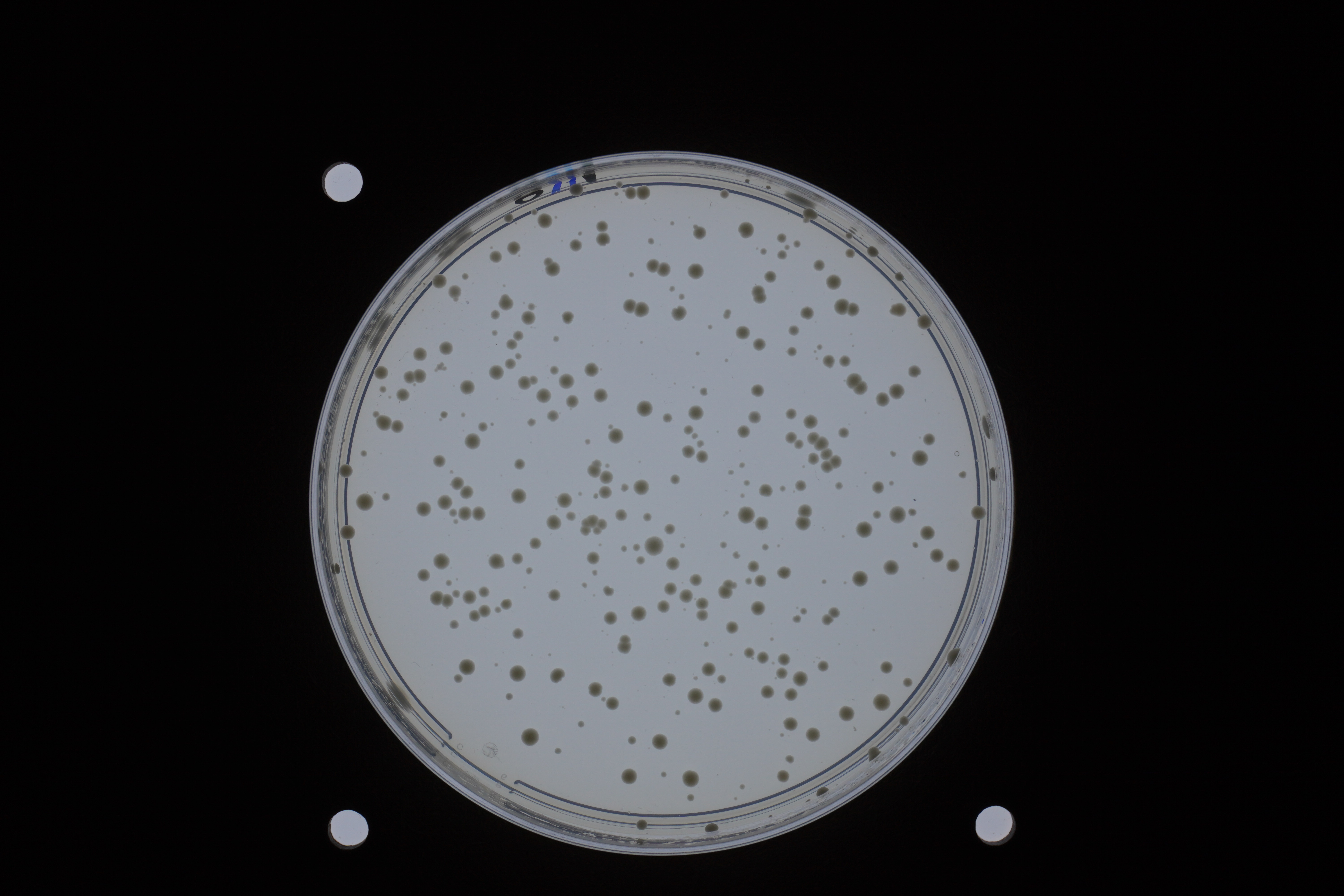

Supplement: Supplementary file 19 — Figure EV1 Source Data [file 44319_2026_702_MOESM19_ESM.zip › Figure EV1_SourceData/EV1A/Images/No fluconazole_H2O_Overexpression_5FOA_1.TIFF]

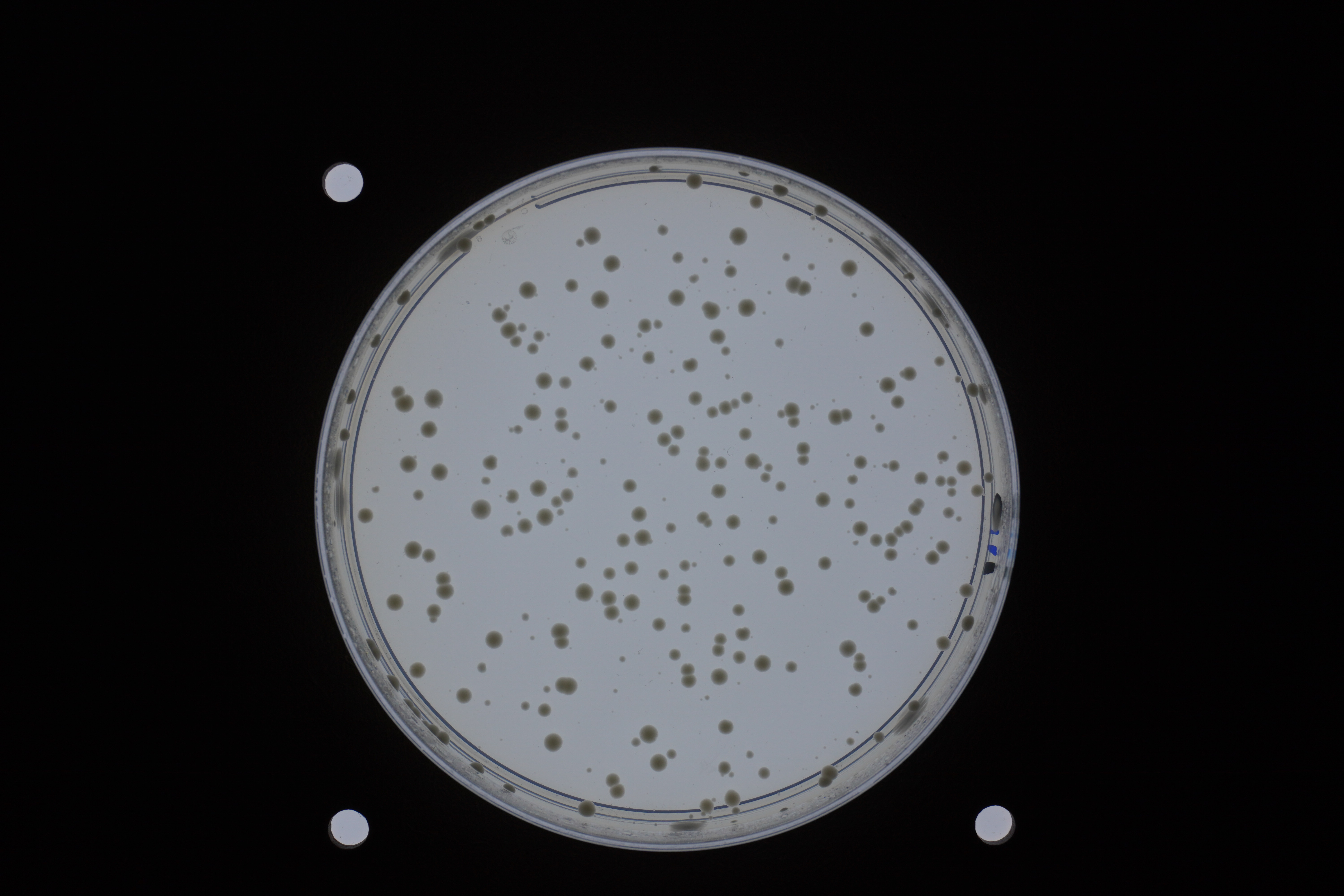

Supplement: Supplementary file 19 — Figure EV1 Source Data [file 44319_2026_702_MOESM19_ESM.zip › Figure EV1_SourceData/EV1A/Images/No fluconazole_H2O_Overexpression_5FOA_10.TIFF]

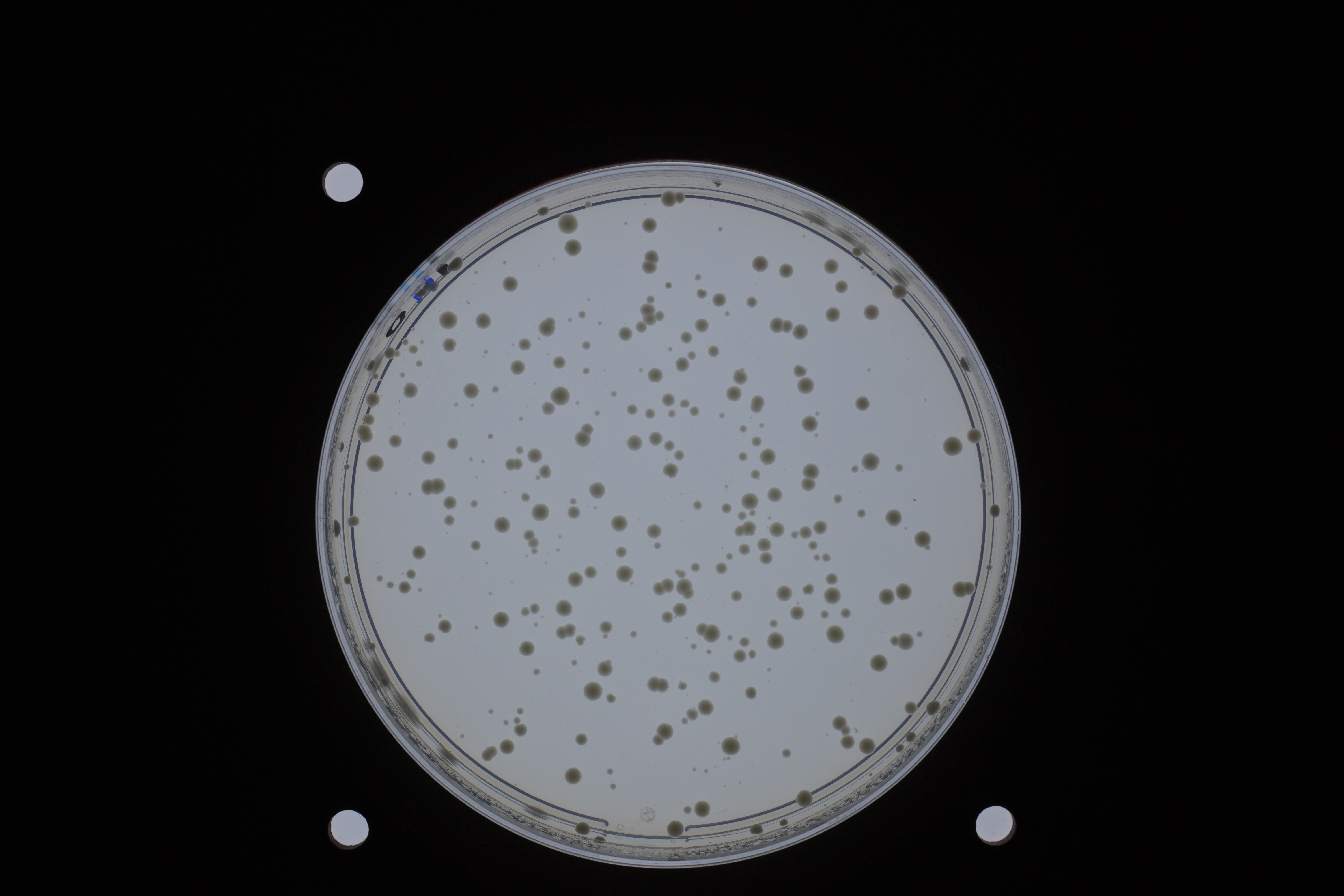

Supplement: Supplementary file 19 — Figure EV1 Source Data [file 44319_2026_702_MOESM19_ESM.zip › Figure EV1_SourceData/EV1A/Images/No fluconazole_H2O_Overexpression_5FOA_2.TIFF]

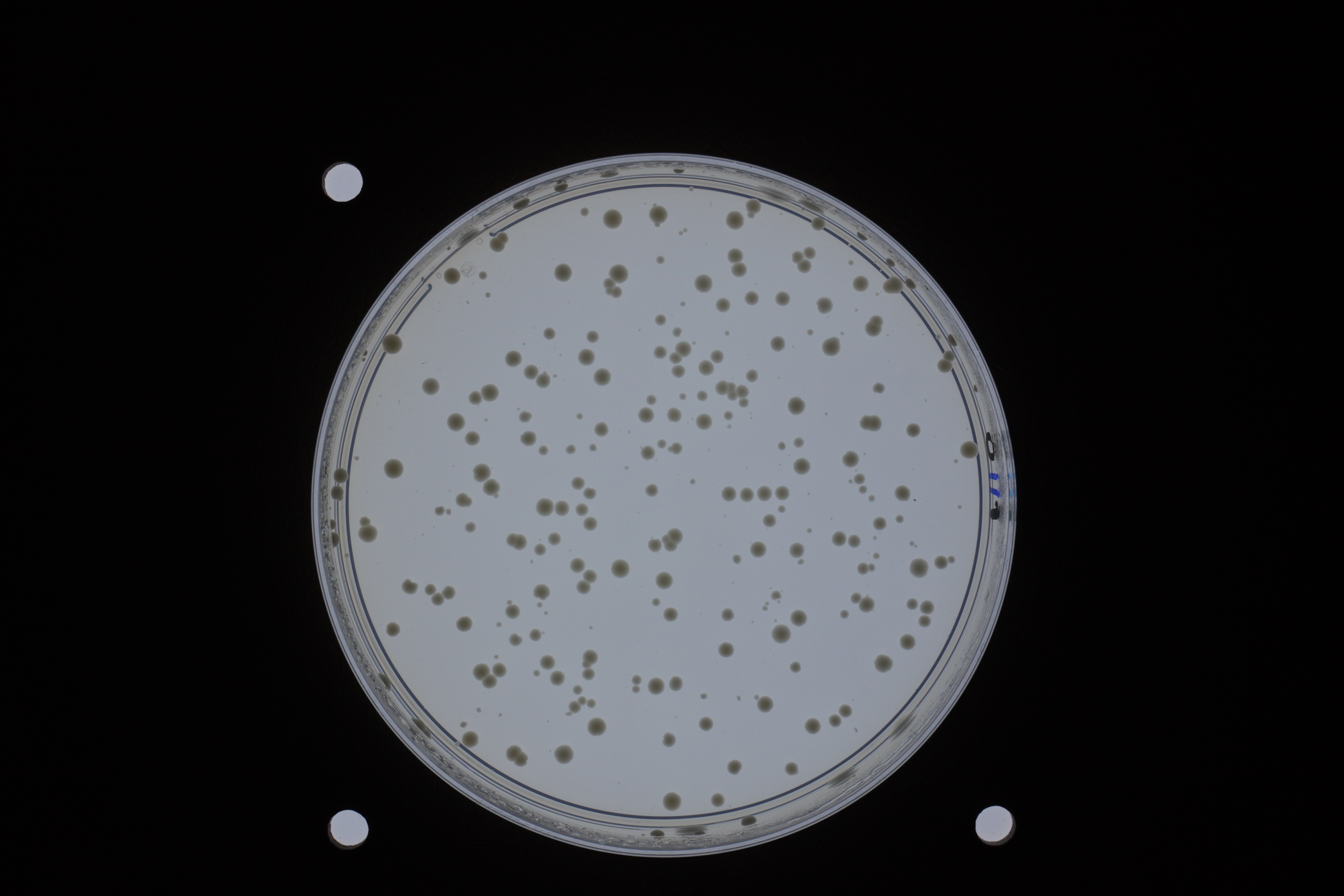

Supplement: Supplementary file 19 — Figure EV1 Source Data [file 44319_2026_702_MOESM19_ESM.zip › Figure EV1_SourceData/EV1A/Images/No fluconazole_H2O_Overexpression_5FOA_3.TIFF]

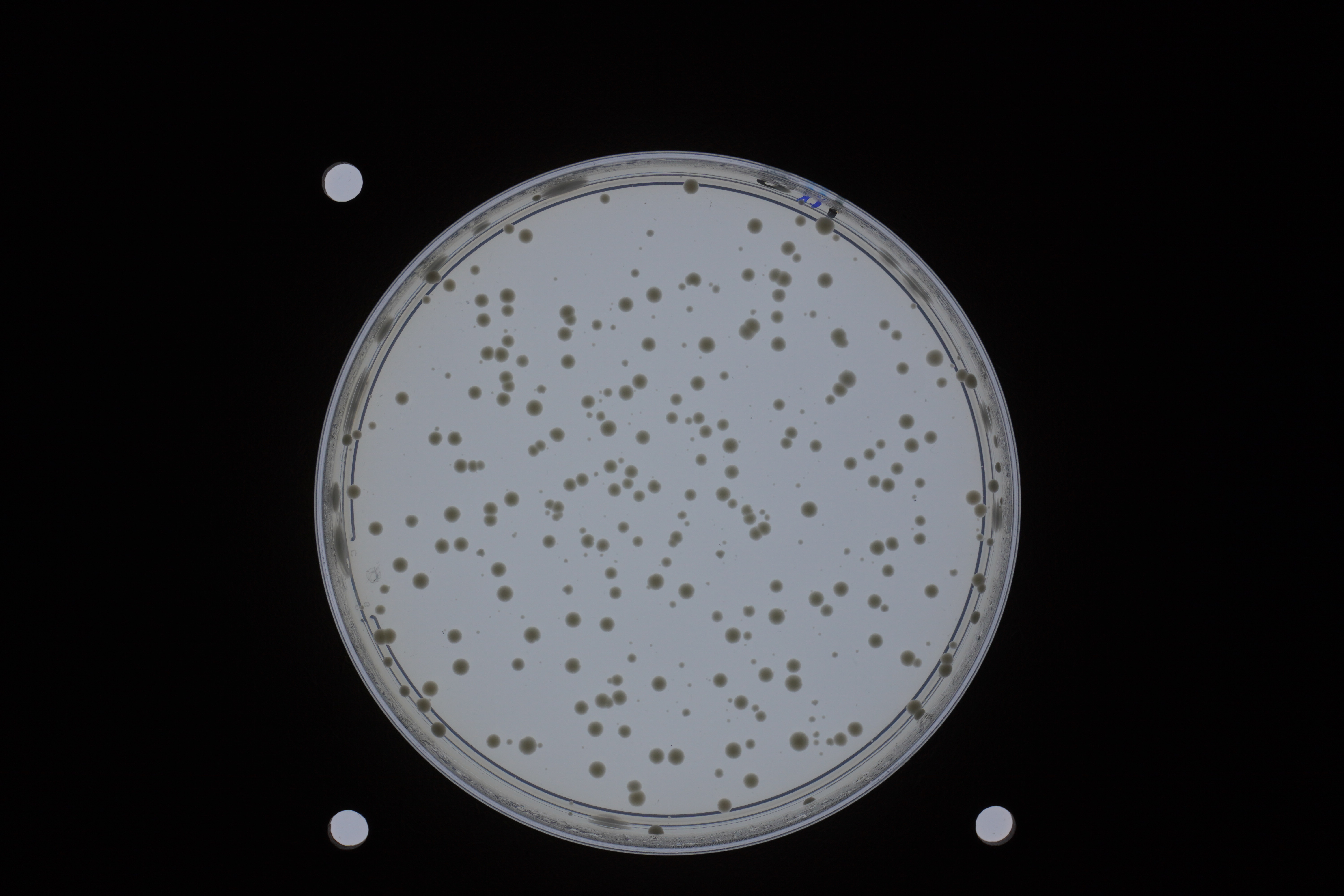

Supplement: Supplementary file 19 — Figure EV1 Source Data [file 44319_2026_702_MOESM19_ESM.zip › Figure EV1_SourceData/EV1A/Images/No fluconazole_H2O_Overexpression_5FOA_4..TIFF]

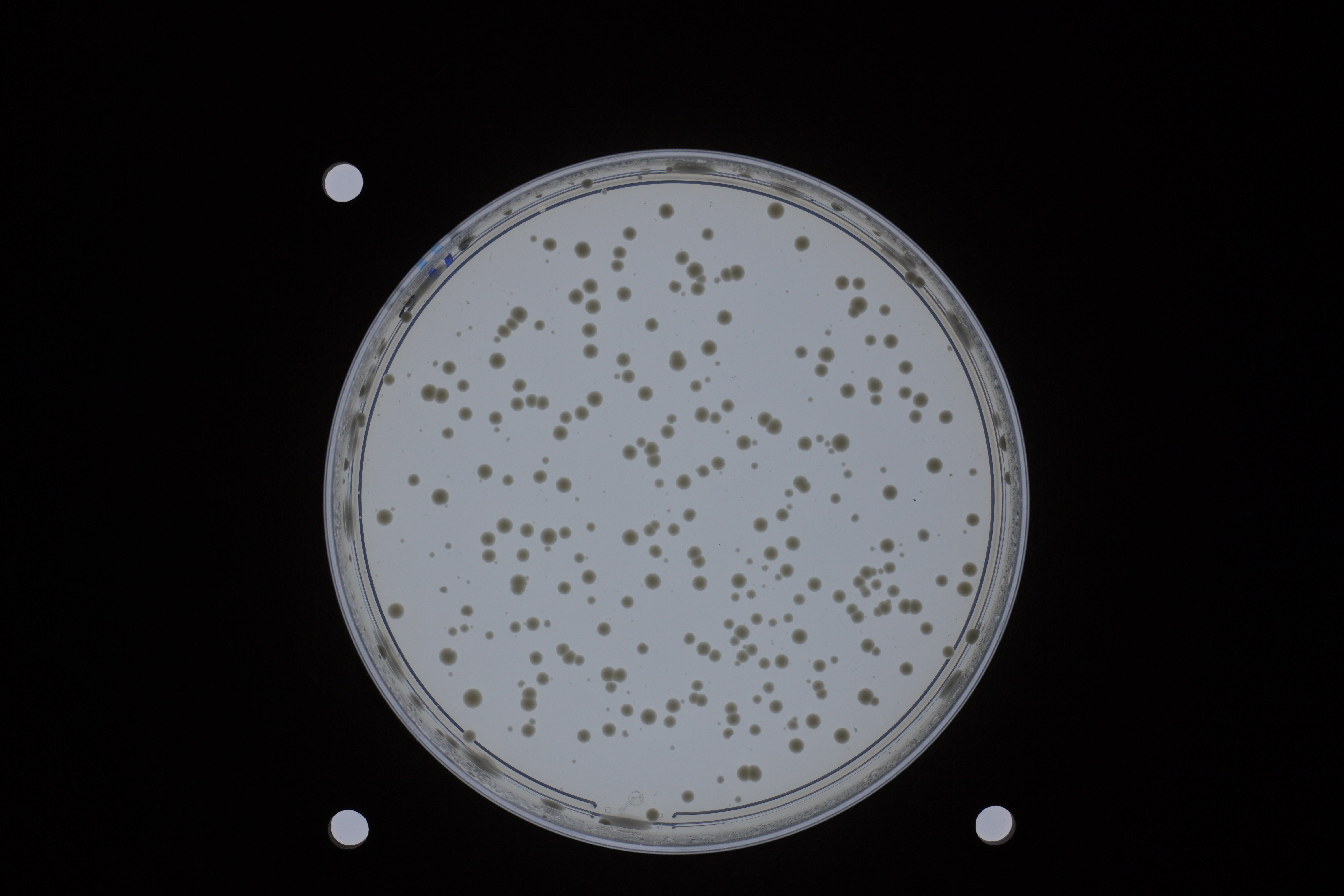

Supplement: Supplementary file 19 — Figure EV1 Source Data [file 44319_2026_702_MOESM19_ESM.zip › Figure EV1_SourceData/EV1A/Images/No fluconazole_H2O_Overexpression_5FOA_5.TIFF]

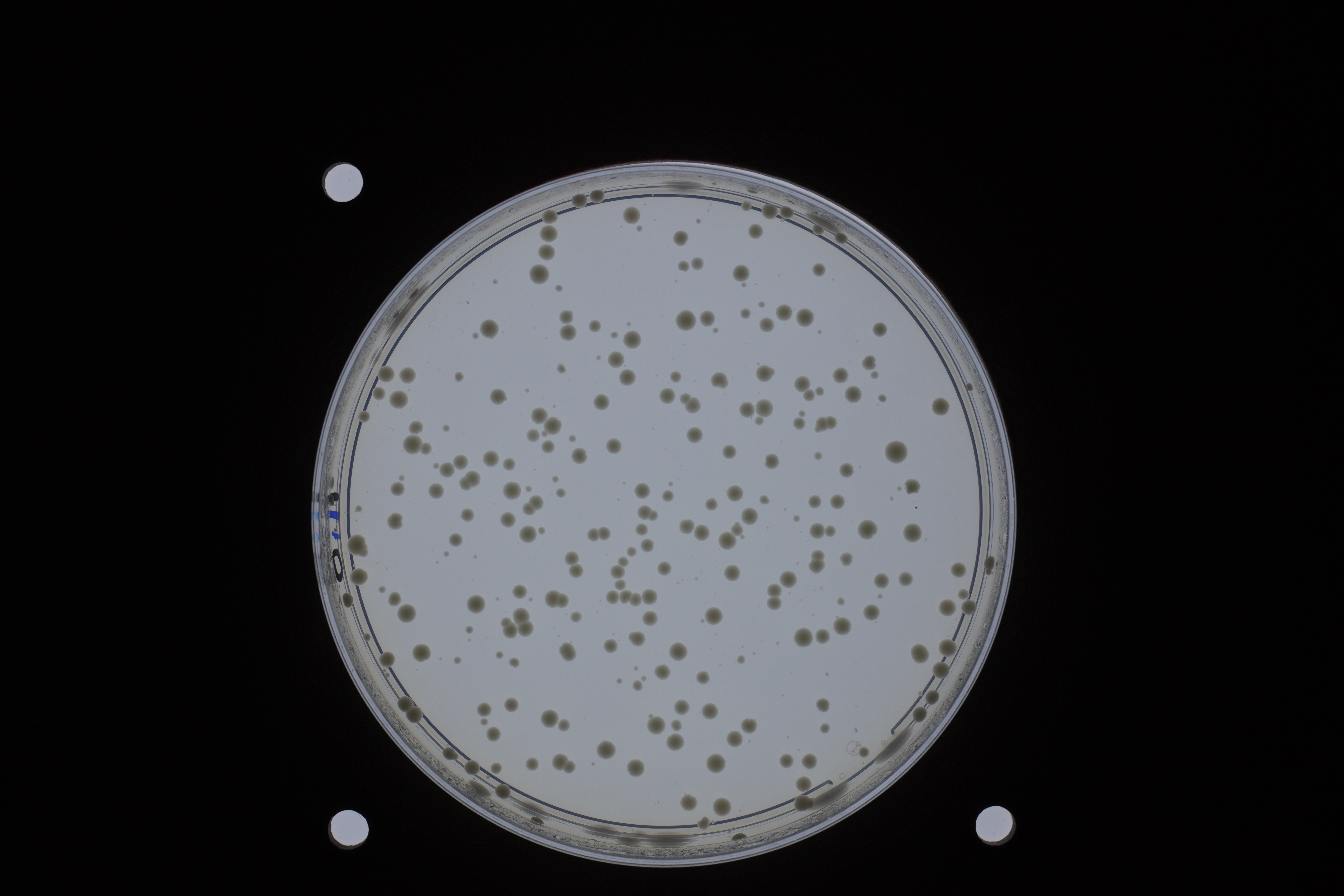

Supplement: Supplementary file 19 — Figure EV1 Source Data [file 44319_2026_702_MOESM19_ESM.zip › Figure EV1_SourceData/EV1A/Images/No fluconazole_H2O_Overexpression_5FOA_6.TIFF]

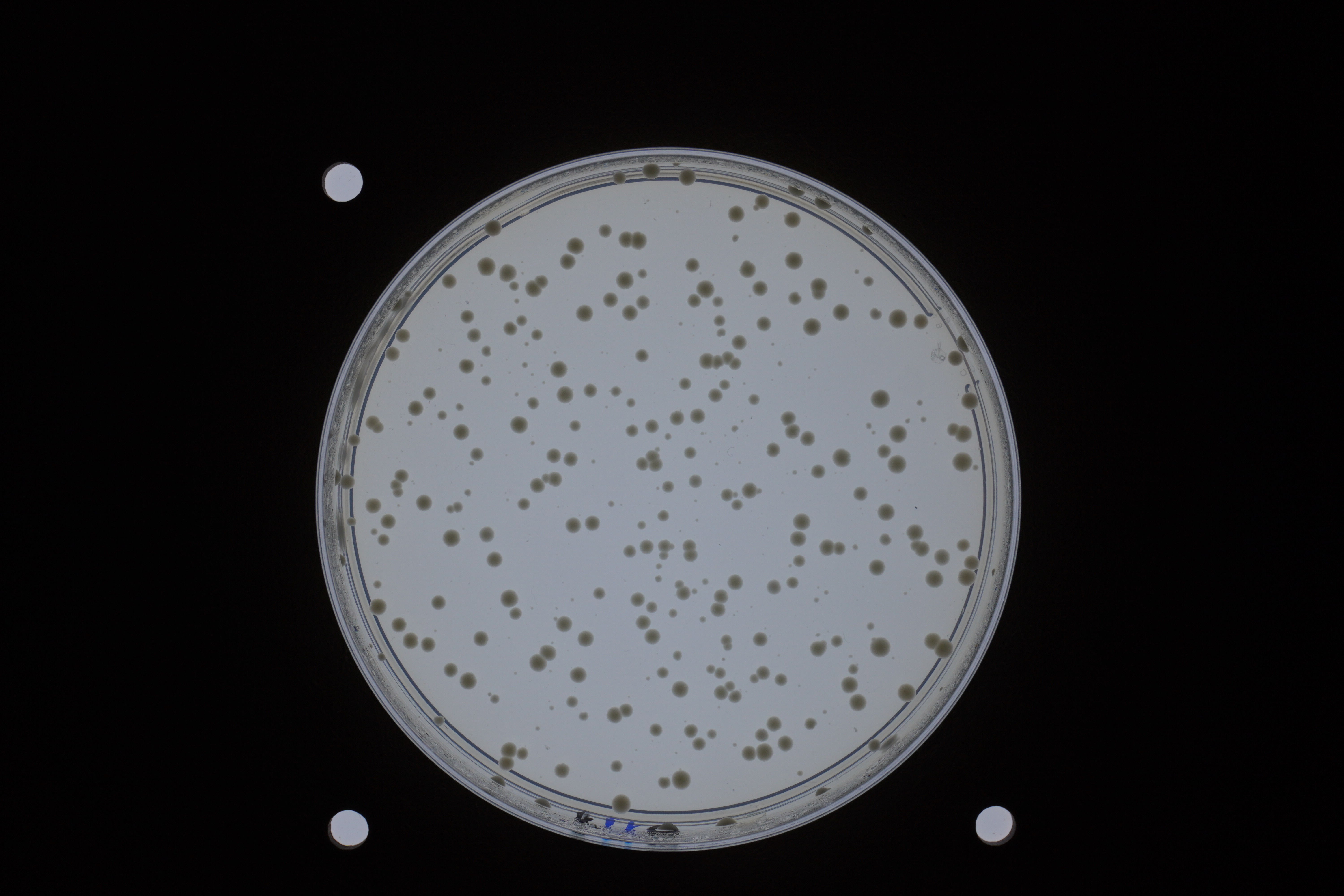

Supplement: Supplementary file 19 — Figure EV1 Source Data [file 44319_2026_702_MOESM19_ESM.zip › Figure EV1_SourceData/EV1A/Images/No fluconazole_H2O_Overexpression_5FOA_7.TIFF]

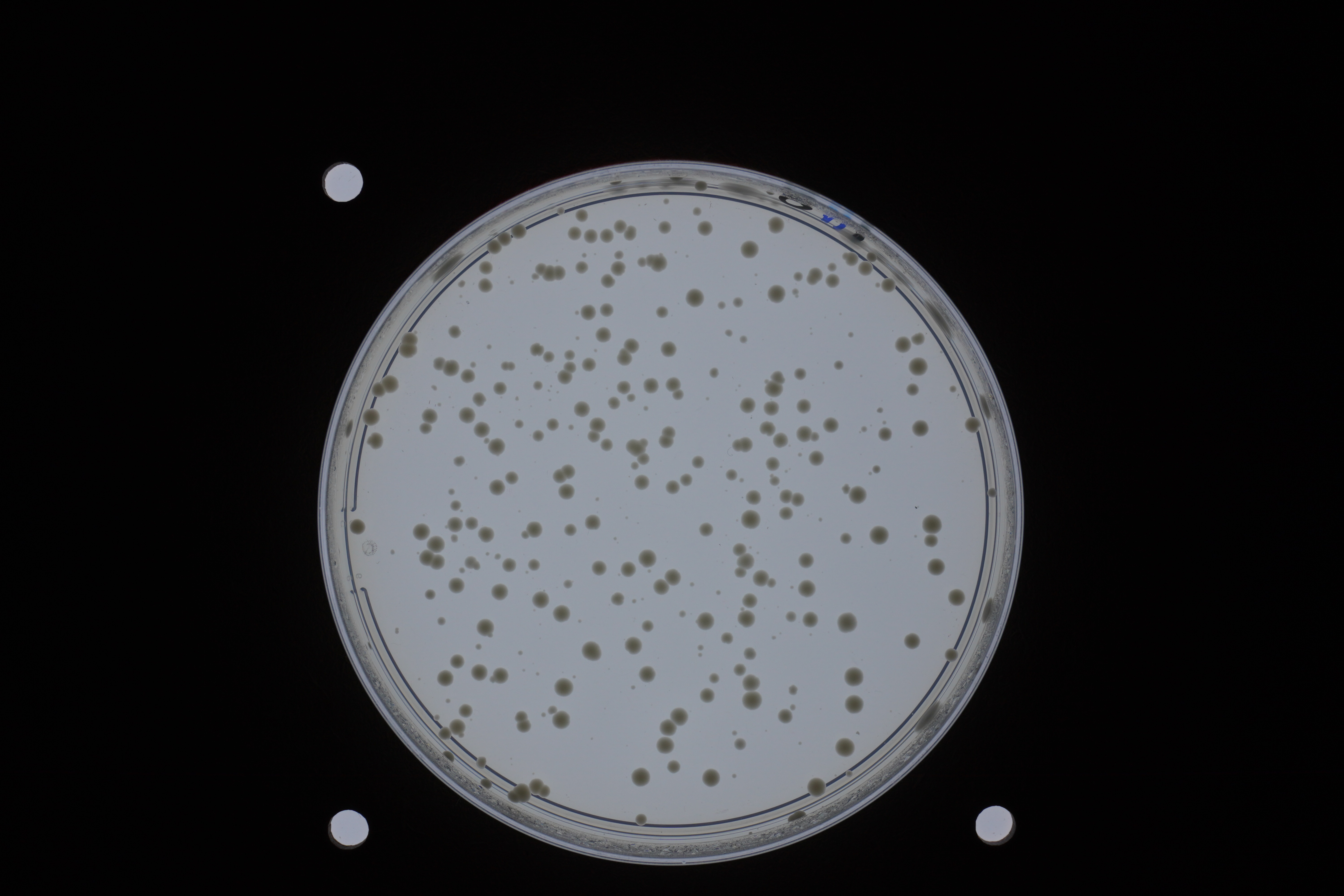

Supplement: Supplementary file 19 — Figure EV1 Source Data [file 44319_2026_702_MOESM19_ESM.zip › Figure EV1_SourceData/EV1A/Images/No fluconazole_H2O_Overexpression_5FOA_8..TIFF]

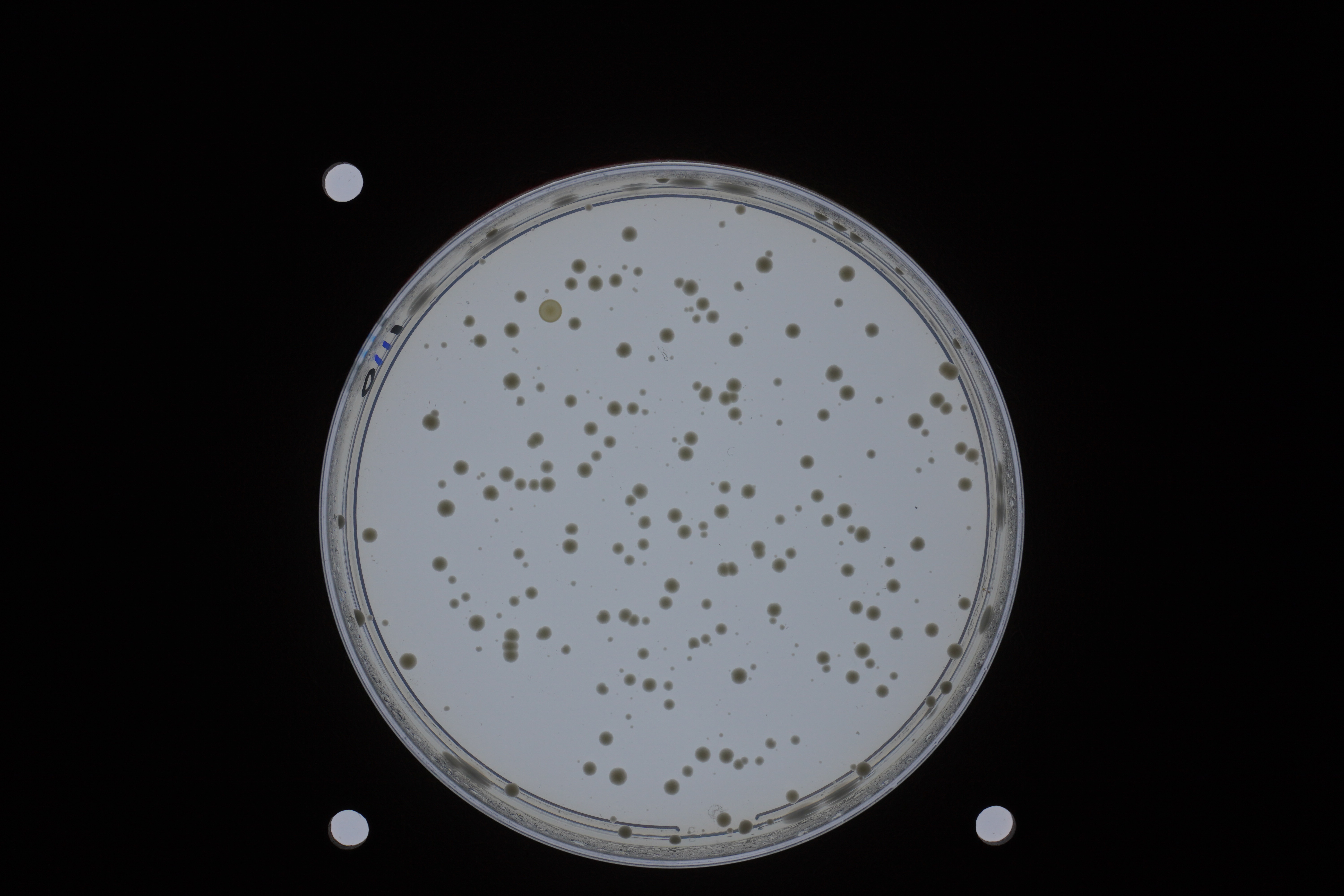

Supplement: Supplementary file 19 — Figure EV1 Source Data [file 44319_2026_702_MOESM19_ESM.zip › Figure EV1_SourceData/EV1A/Images/No fluconazole_H2O_Overexpression_5FOA_9.TIFF]

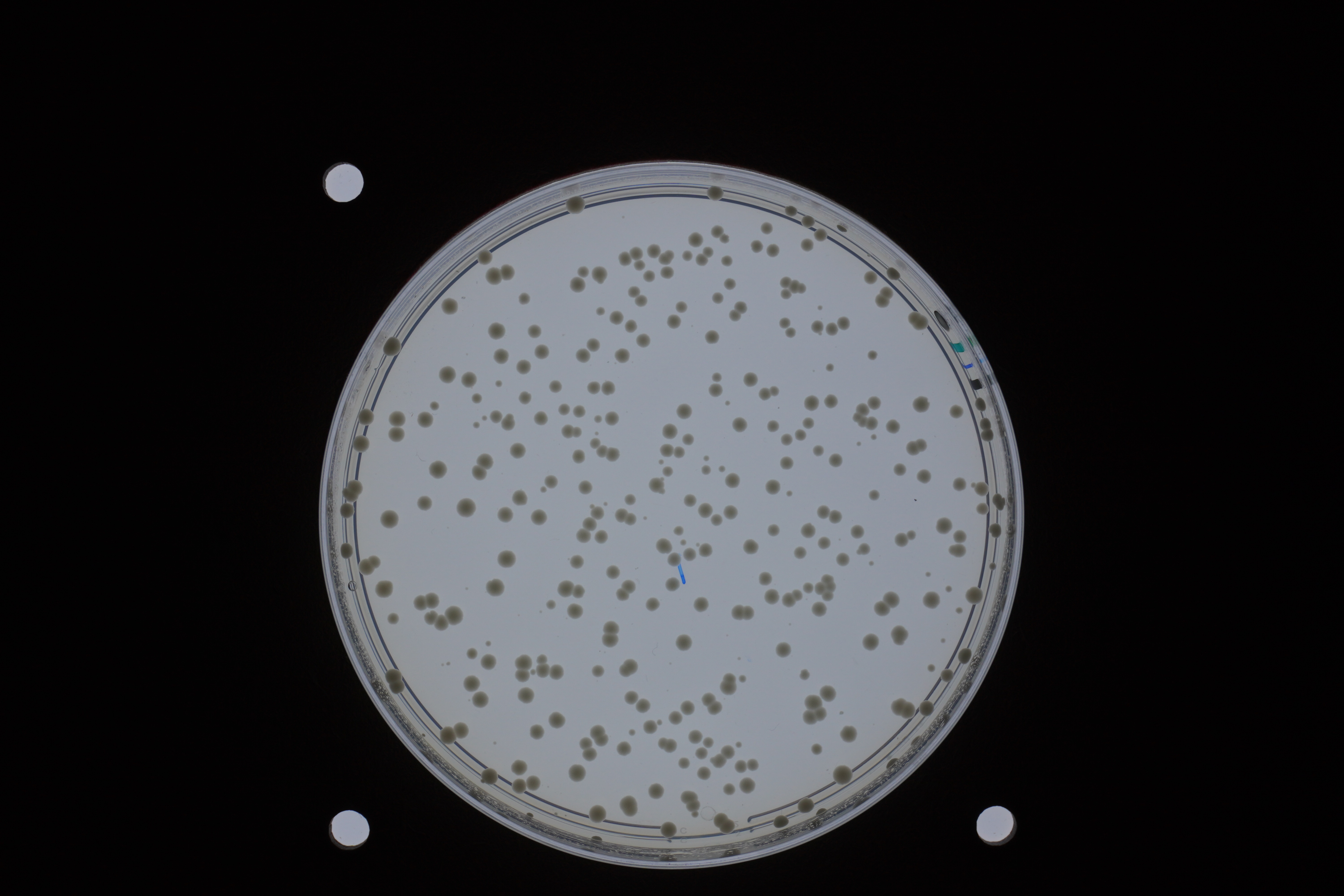

Supplement: Supplementary file 19 — Figure EV1 Source Data [file 44319_2026_702_MOESM19_ESM.zip › Figure EV1_SourceData/EV1A/Images/No fluconazole_H2O_Overexpression_SCmURA_1.TIFF]

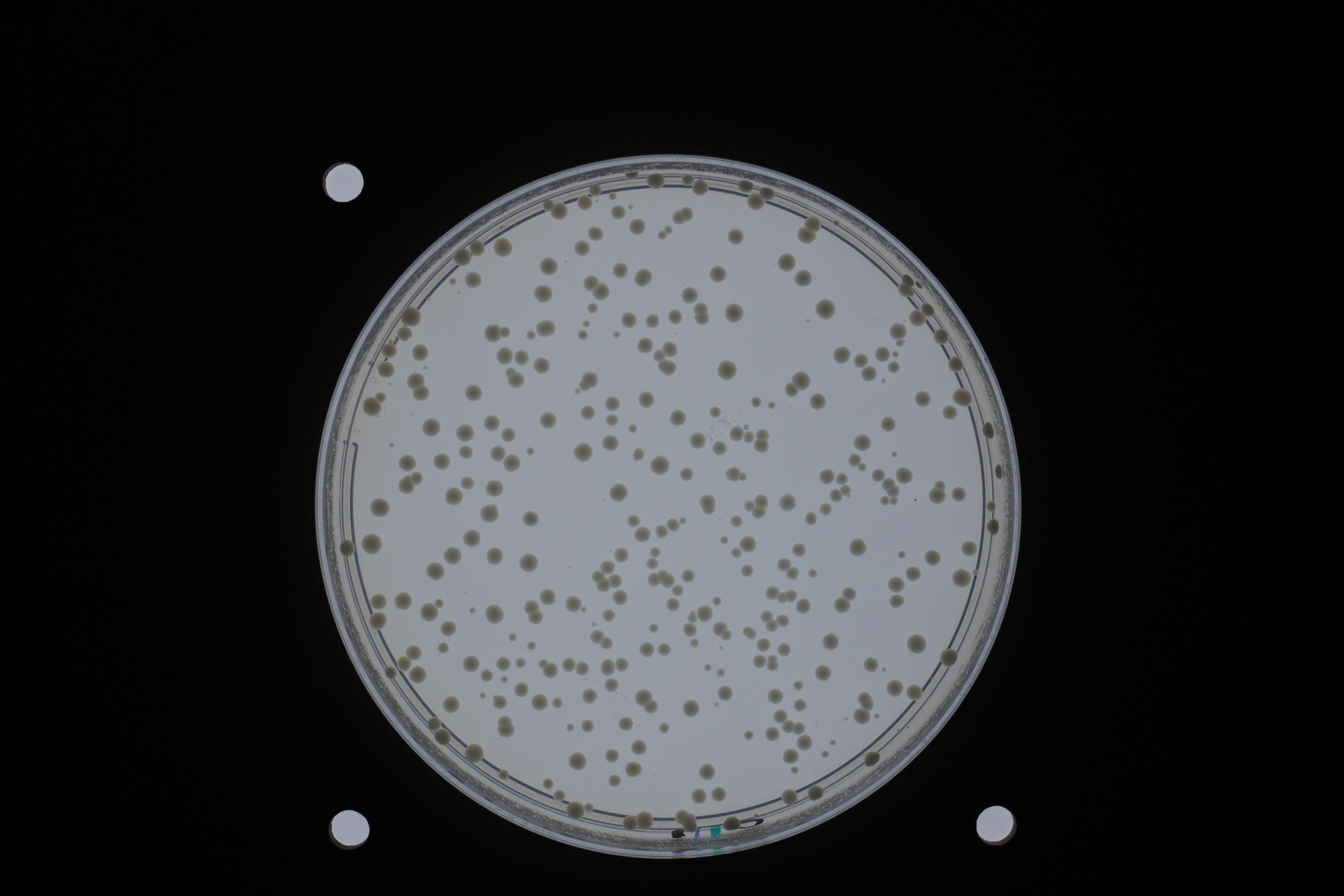

Supplement: Supplementary file 19 — Figure EV1 Source Data [file 44319_2026_702_MOESM19_ESM.zip › Figure EV1_SourceData/EV1A/Images/No fluconazole_H2O_Overexpression_SCmURA_10.TIFF]

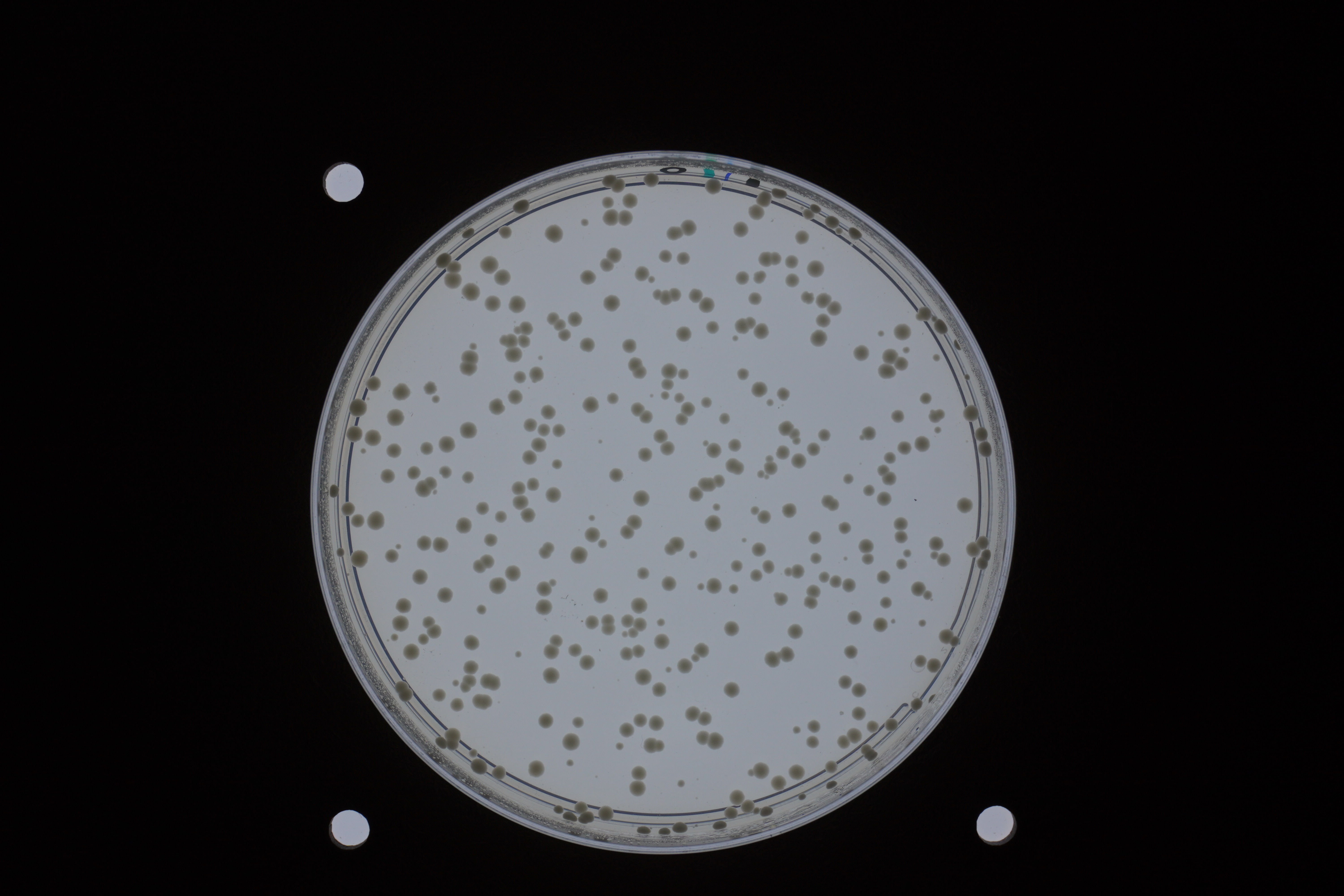

Supplement: Supplementary file 19 — Figure EV1 Source Data [file 44319_2026_702_MOESM19_ESM.zip › Figure EV1_SourceData/EV1A/Images/No fluconazole_H2O_Overexpression_SCmURA_2.TIFF]

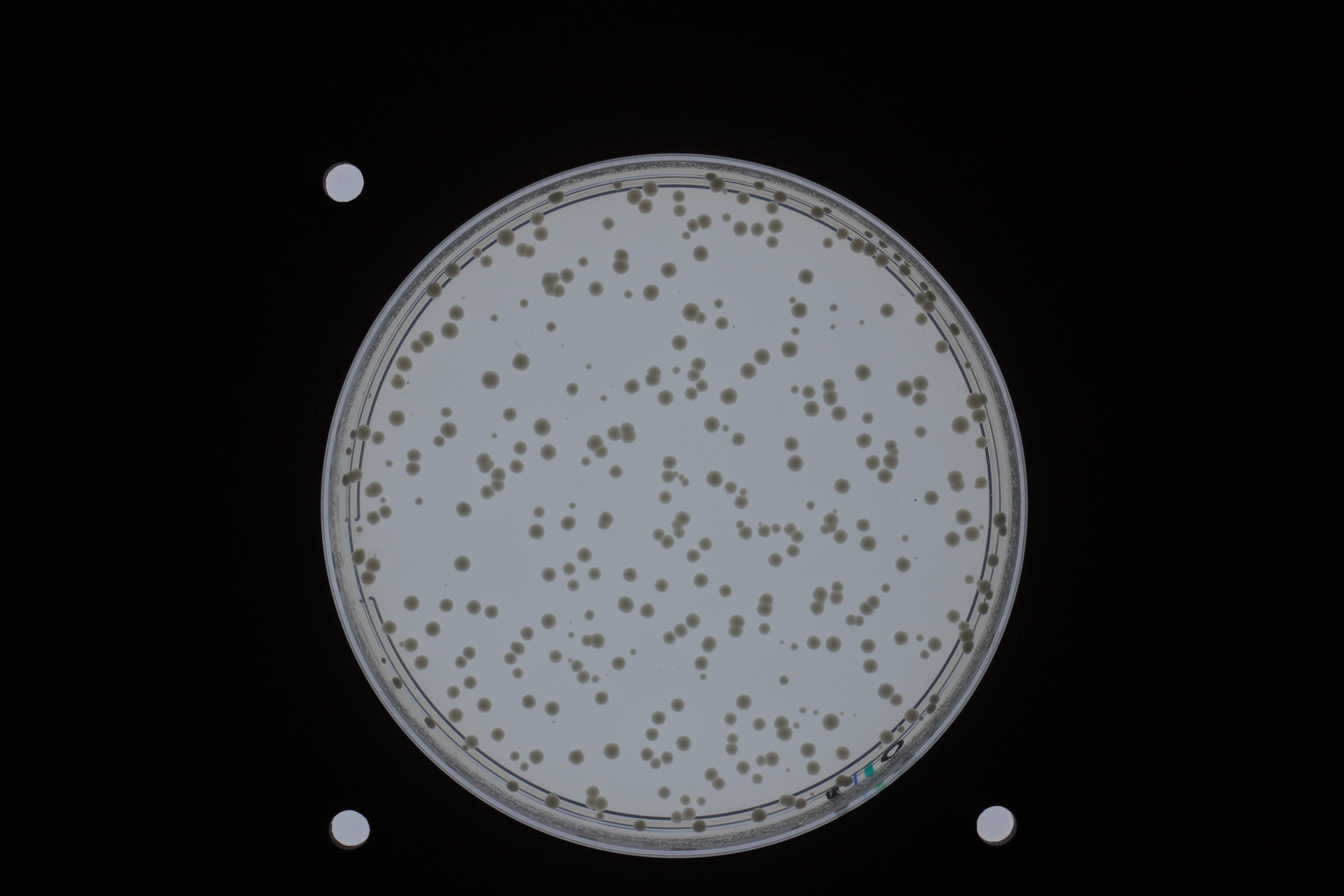

Supplement: Supplementary file 19 — Figure EV1 Source Data [file 44319_2026_702_MOESM19_ESM.zip › Figure EV1_SourceData/EV1A/Images/No fluconazole_H2O_Overexpression_SCmURA_3.TIFF]

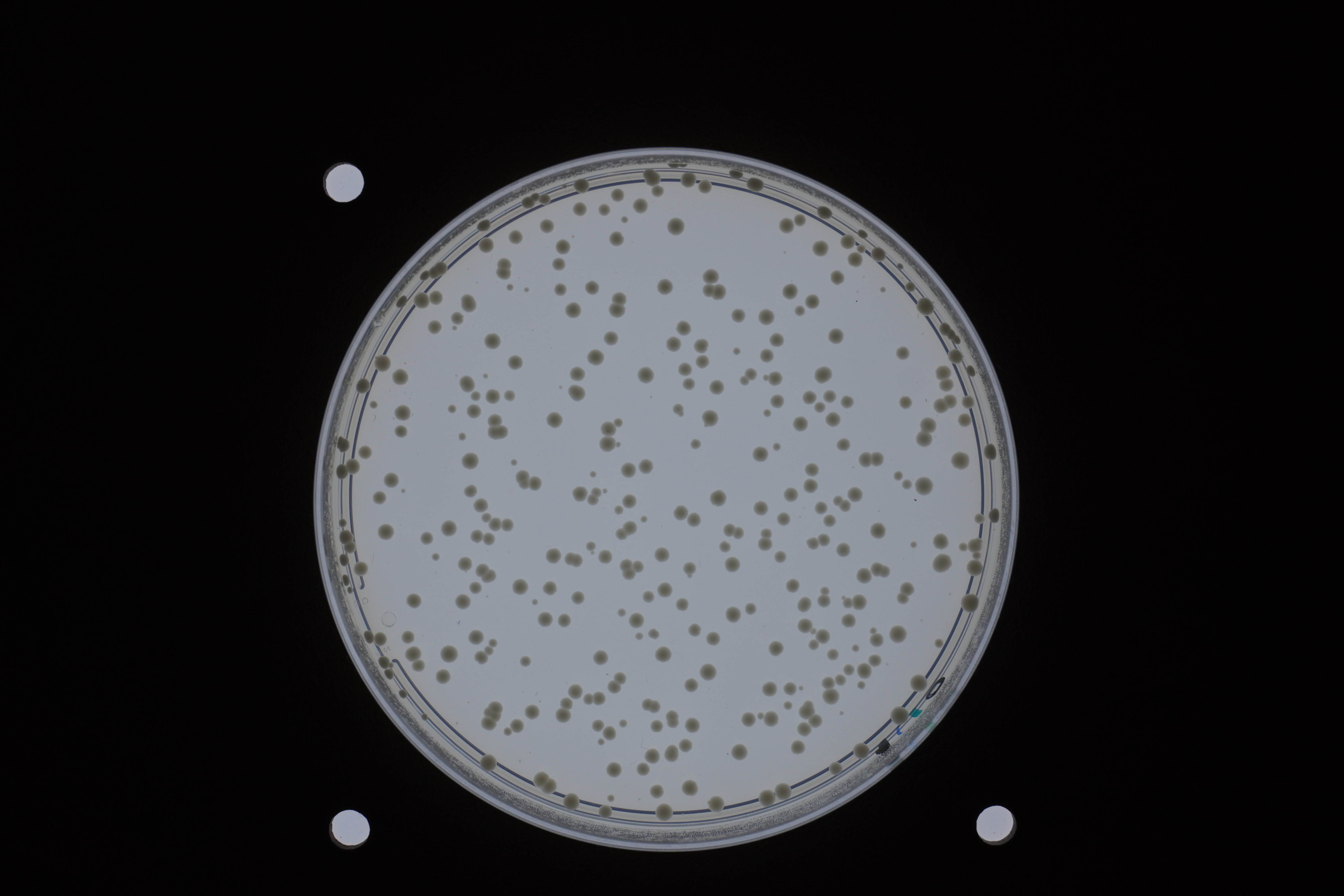

Supplement: Supplementary file 19 — Figure EV1 Source Data [file 44319_2026_702_MOESM19_ESM.zip › Figure EV1_SourceData/EV1A/Images/No fluconazole_H2O_Overexpression_SCmURA_4.TIFF]

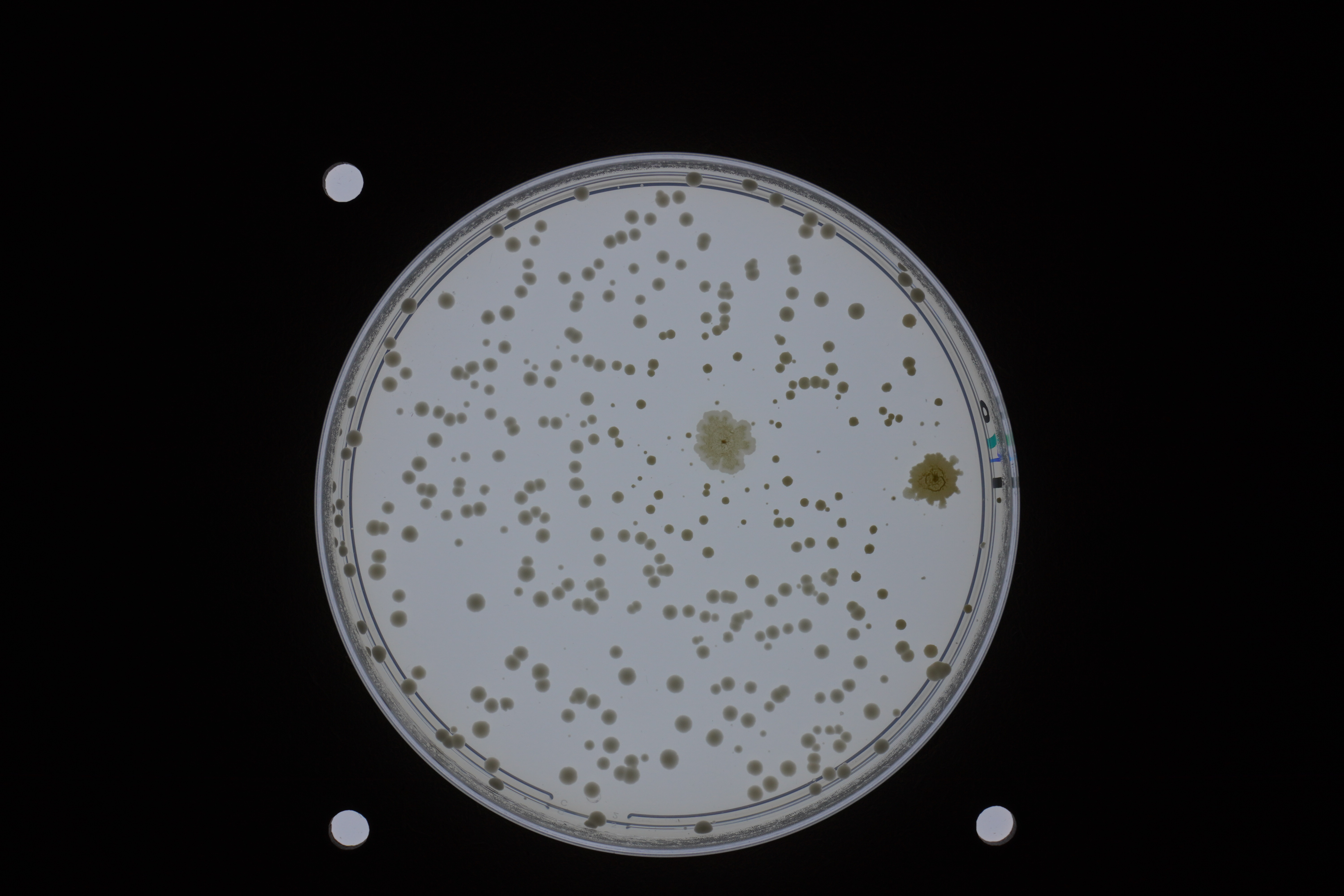

Supplement: Supplementary file 19 — Figure EV1 Source Data [file 44319_2026_702_MOESM19_ESM.zip › Figure EV1_SourceData/EV1A/Images/No fluconazole_H2O_Overexpression_SCmURA_5.TIFF]

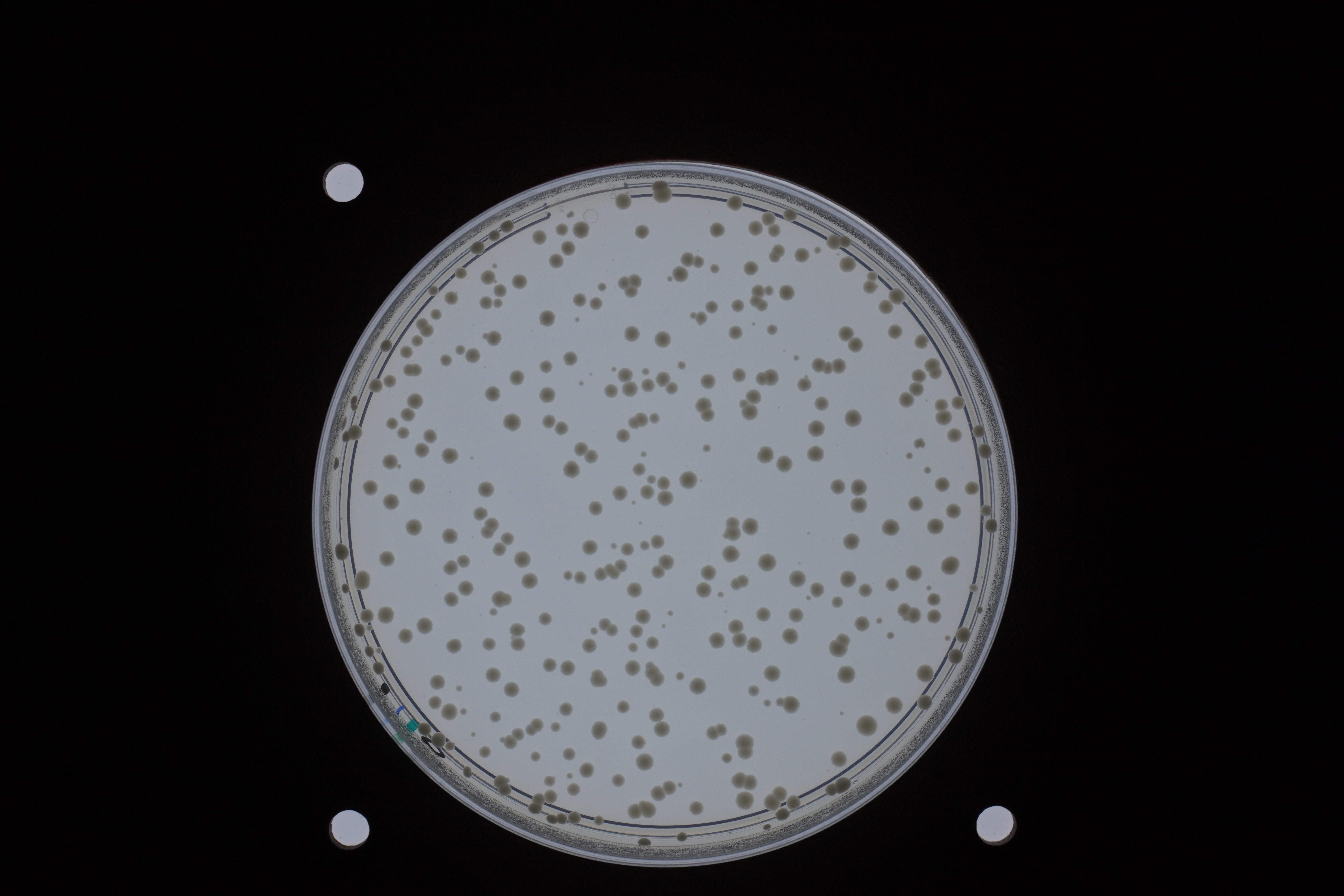

Supplement: Supplementary file 19 — Figure EV1 Source Data [file 44319_2026_702_MOESM19_ESM.zip › Figure EV1_SourceData/EV1A/Images/No fluconazole_H2O_Overexpression_SCmURA_6.TIFF]

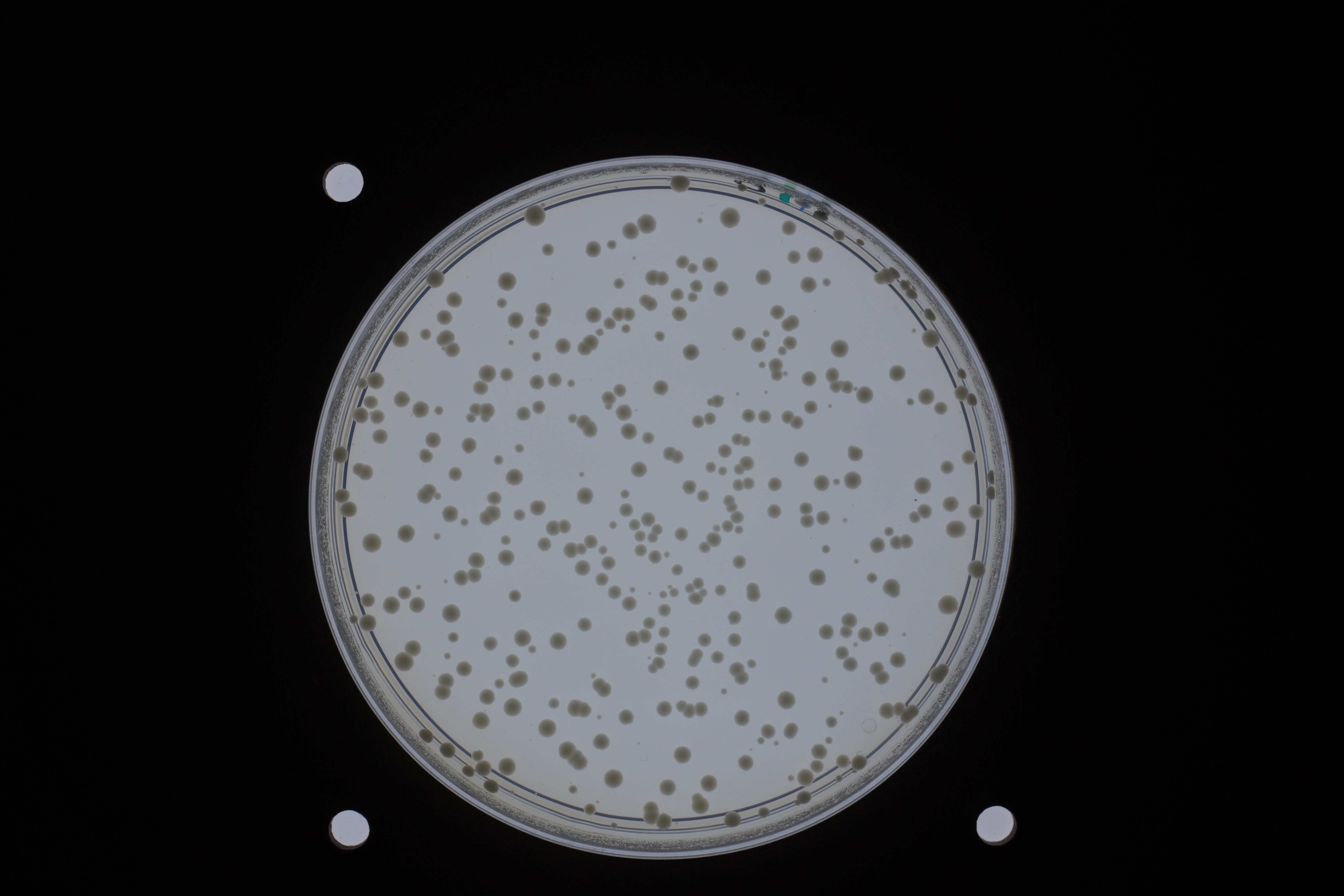

Supplement: Supplementary file 19 — Figure EV1 Source Data [file 44319_2026_702_MOESM19_ESM.zip › Figure EV1_SourceData/EV1A/Images/No fluconazole_H2O_Overexpression_SCmURA_7.TIFF]

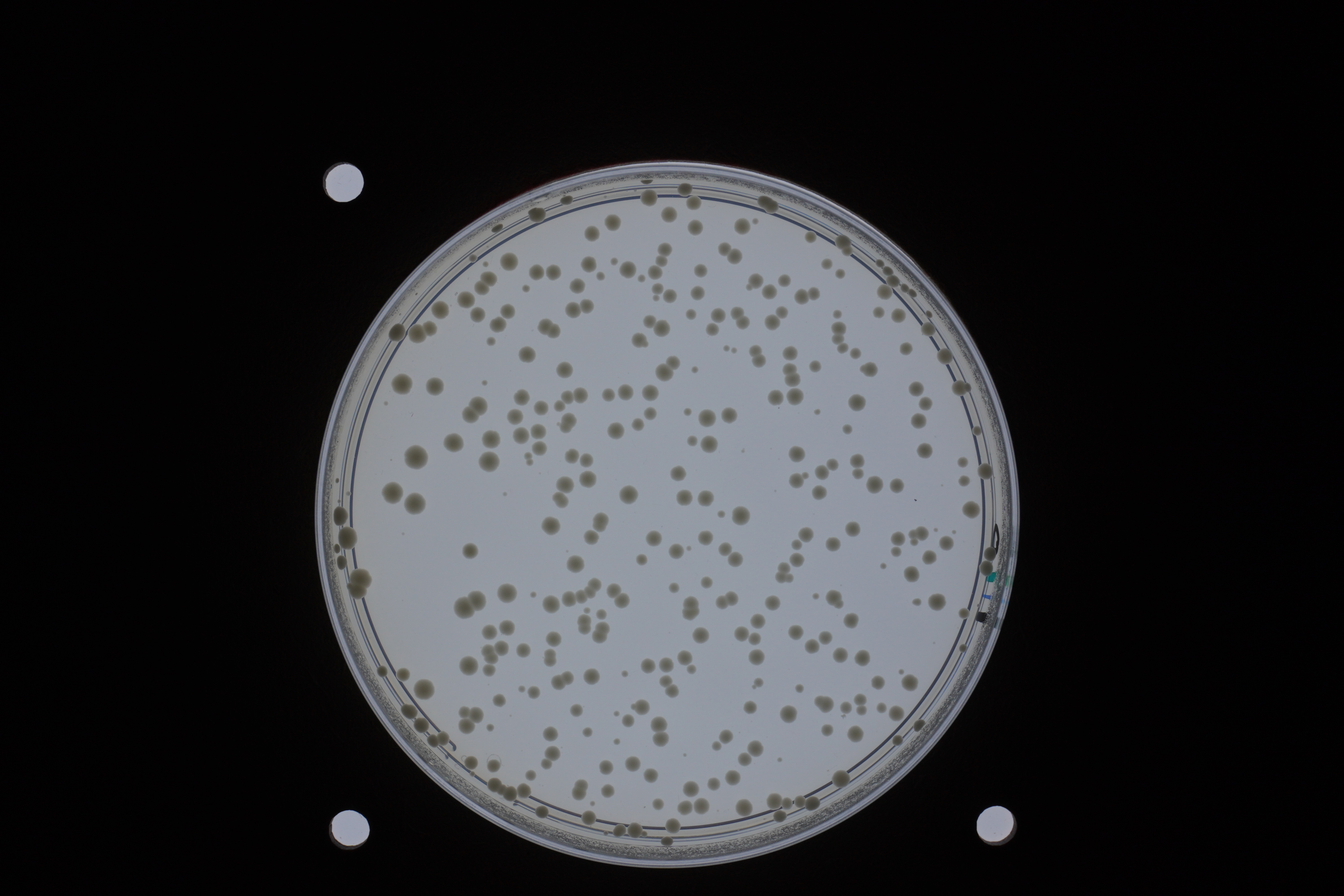

Supplement: Supplementary file 19 — Figure EV1 Source Data [file 44319_2026_702_MOESM19_ESM.zip › Figure EV1_SourceData/EV1A/Images/No fluconazole_H2O_Overexpression_SCmURA_8.TIFF]

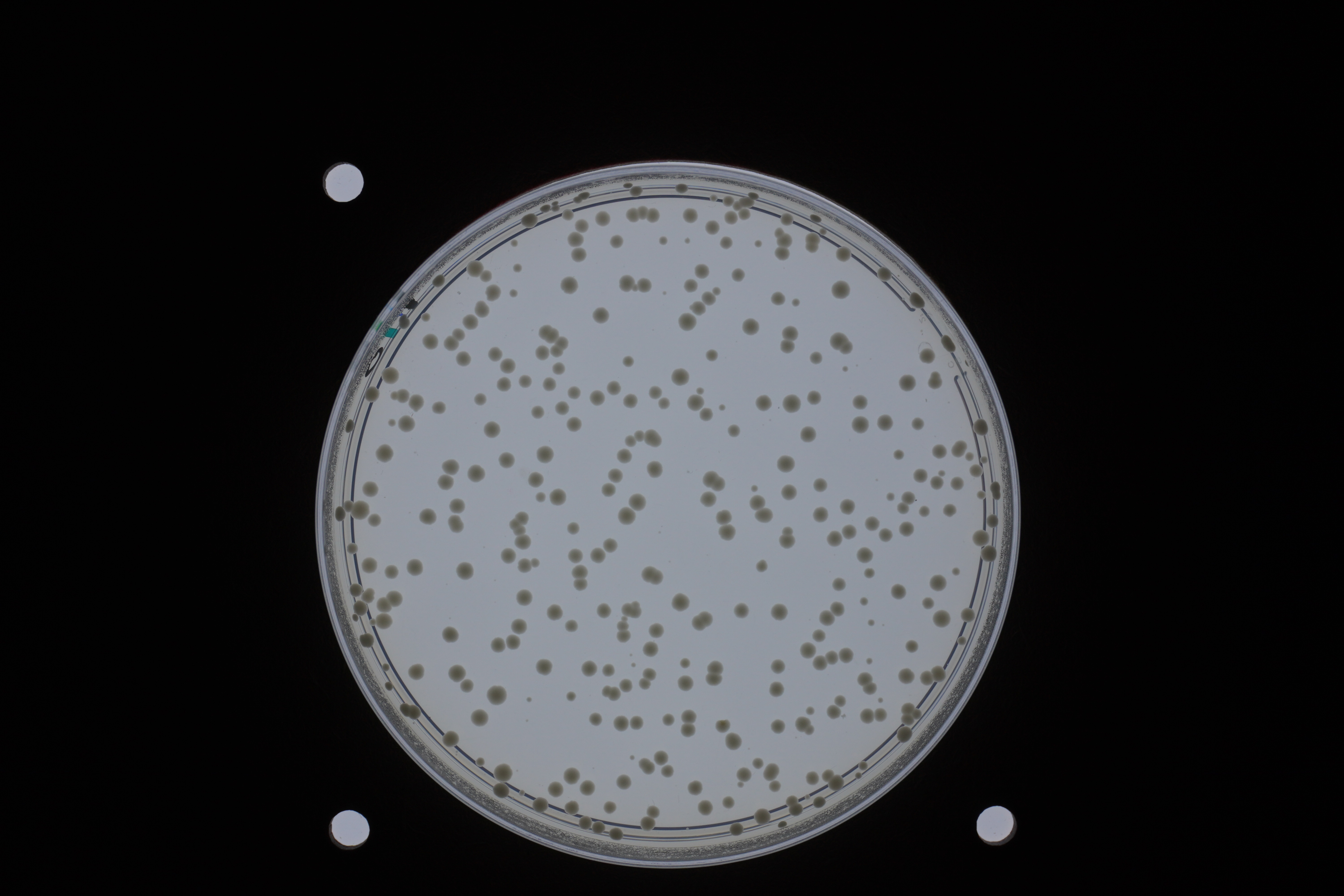

Supplement: Supplementary file 19 — Figure EV1 Source Data [file 44319_2026_702_MOESM19_ESM.zip › Figure EV1_SourceData/EV1A/Images/No fluconazole_H2O_Overexpression_SCmURA_9.TIFF]

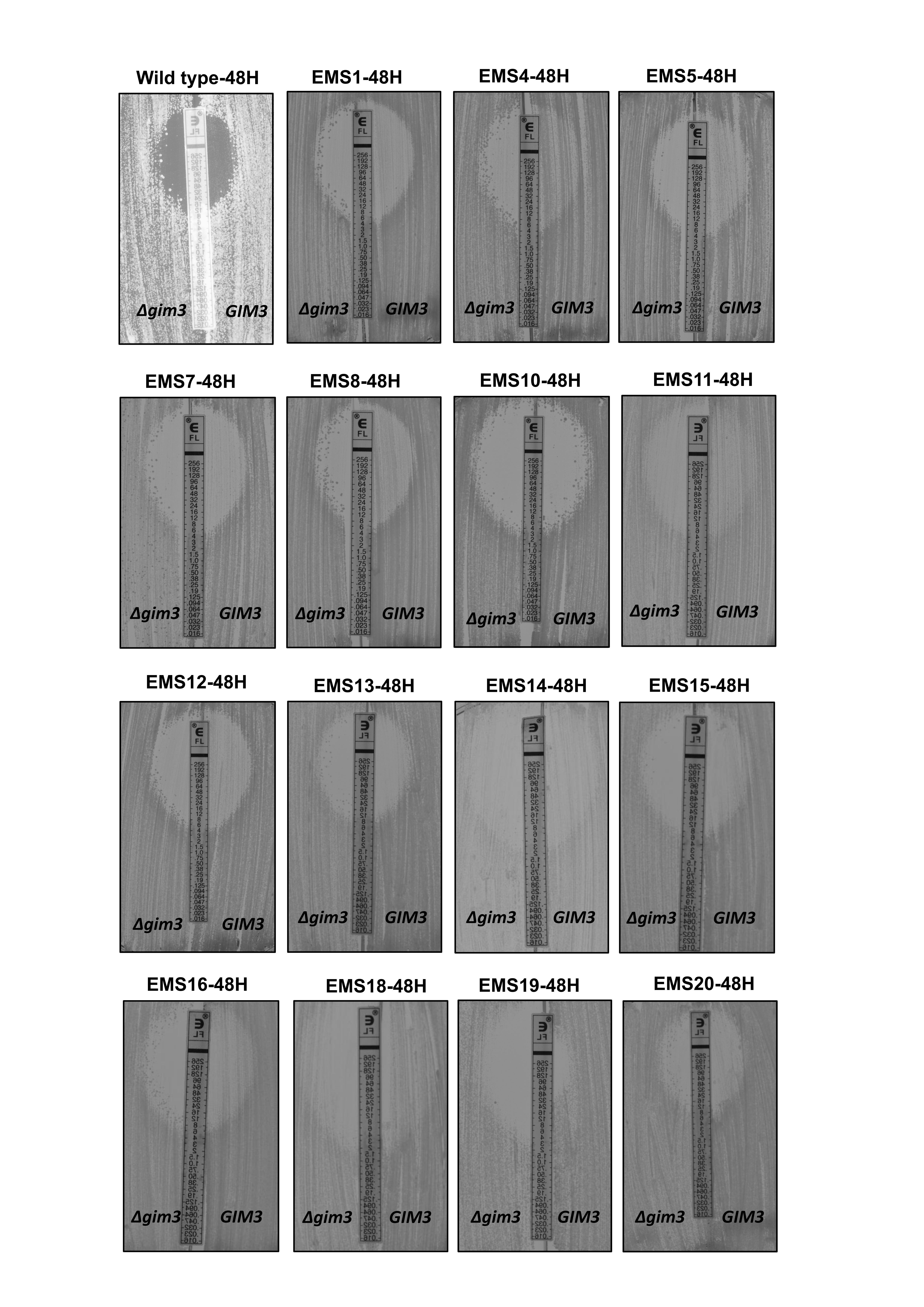

Supplement: Supplementary file 21 — Figure EV3 Source Data [file 44319_2026_702_MOESM21_ESM.zip › Figure EV3_SourceData/EV3E_SourceData/Images/Page 1.png]

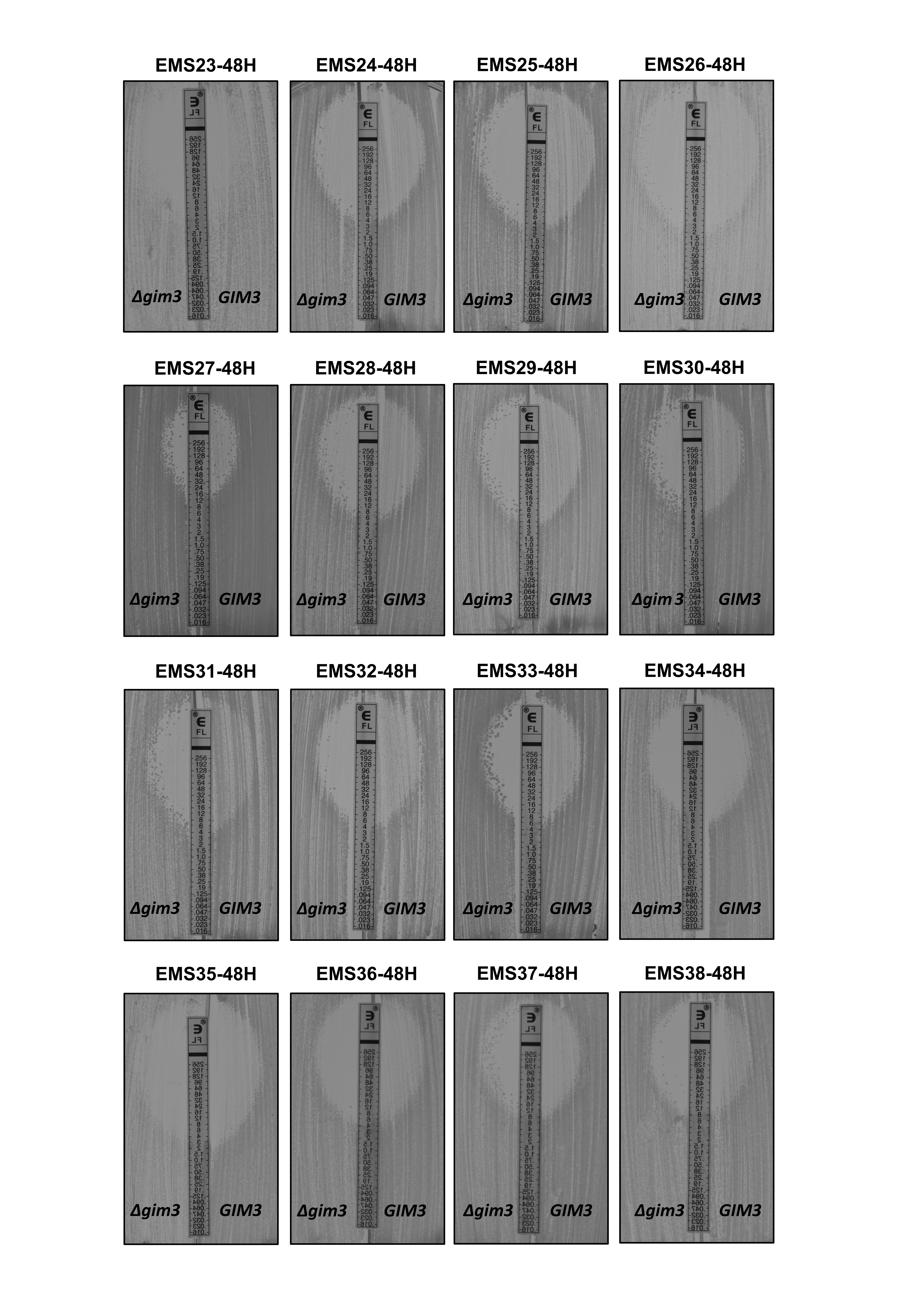

Supplement: Supplementary file 21 — Figure EV3 Source Data [file 44319_2026_702_MOESM21_ESM.zip › Figure EV3_SourceData/EV3E_SourceData/Images/Page 2.png]
