# Supplementary figures and images for: Gim3 buffers and potentiates de novo mutations that affect fluconazole susceptibility in yeast (part 2 of 2)
Source: EMBO Rep. 2026 Feb 17;27(6):1510–39. doi: 10.1038/s44319-026-00702-x (PMC13022404; doi:10.1038/s44319-026-00702-x)

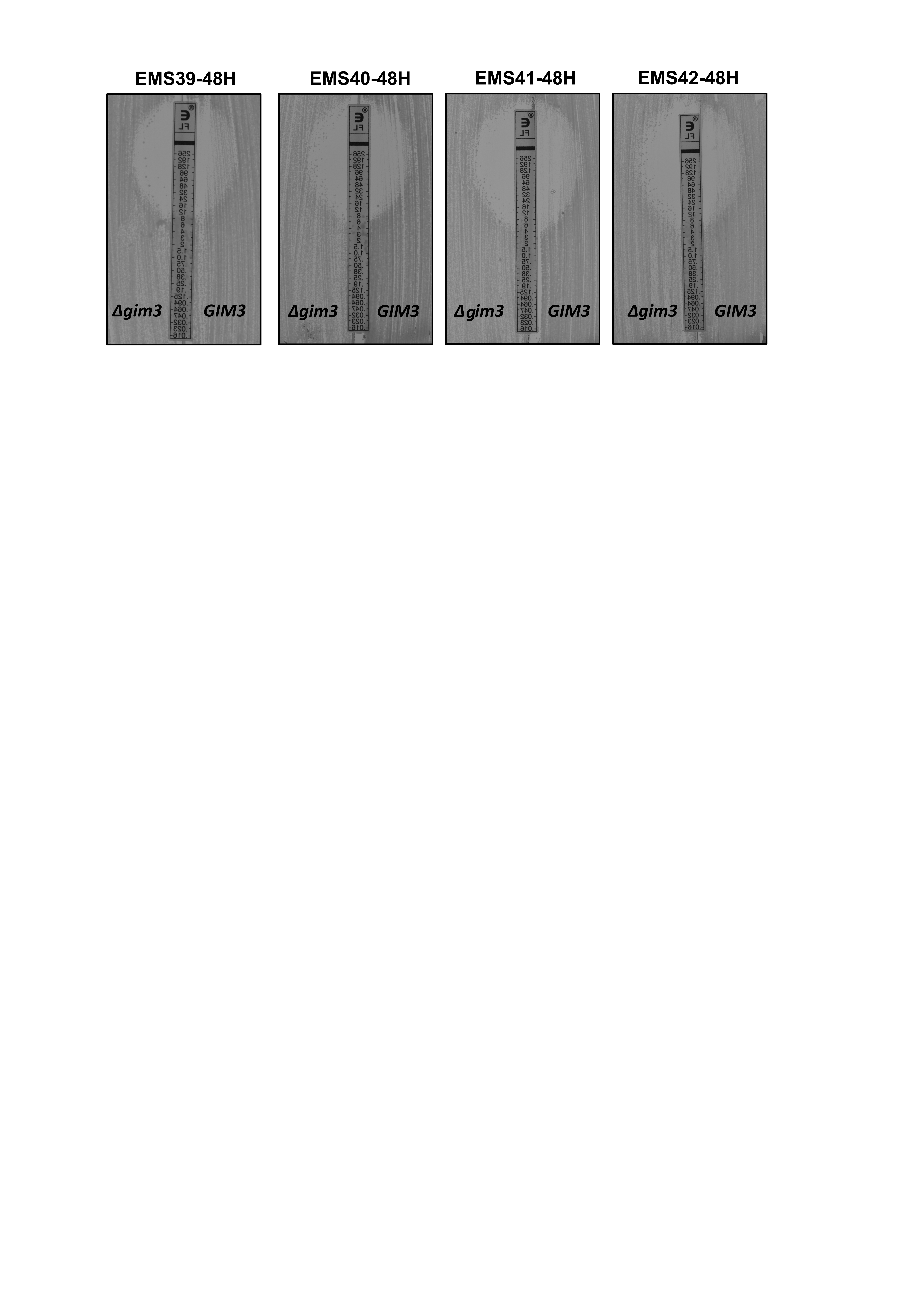

Supplement: Supplementary file 21 — Figure EV3 Source Data [file 44319_2026_702_MOESM21_ESM.zip › Figure EV3_SourceData/EV3E_SourceData/Images/Page 3.png]

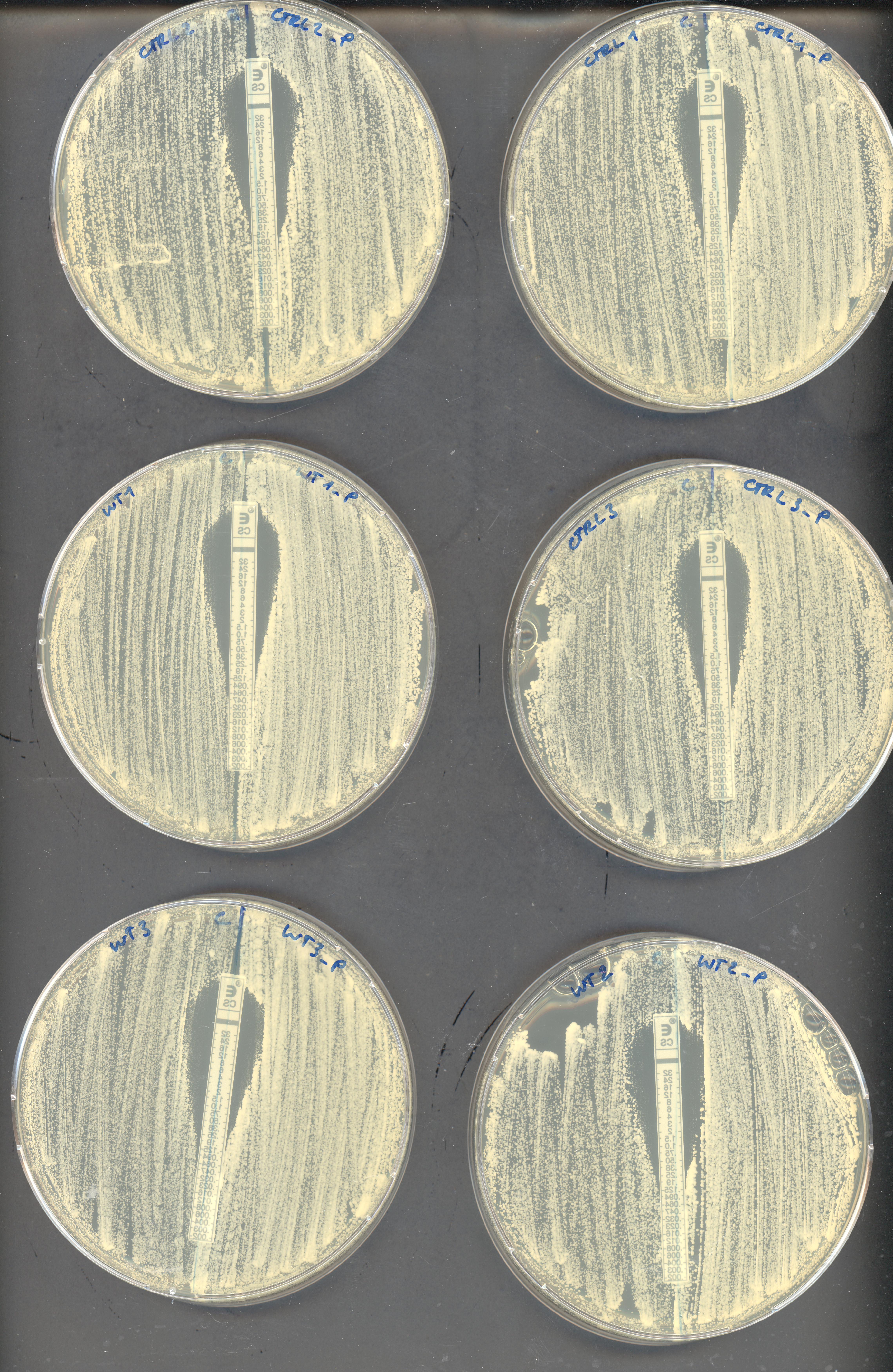

Supplement: Supplementary file 21 — Figure EV3 Source Data [file 44319_2026_702_MOESM21_ESM.zip › Figure EV3_SourceData/EV3_C&D_SourceData/Images/48hr/cerevisiae_Casp_1.TIFF]

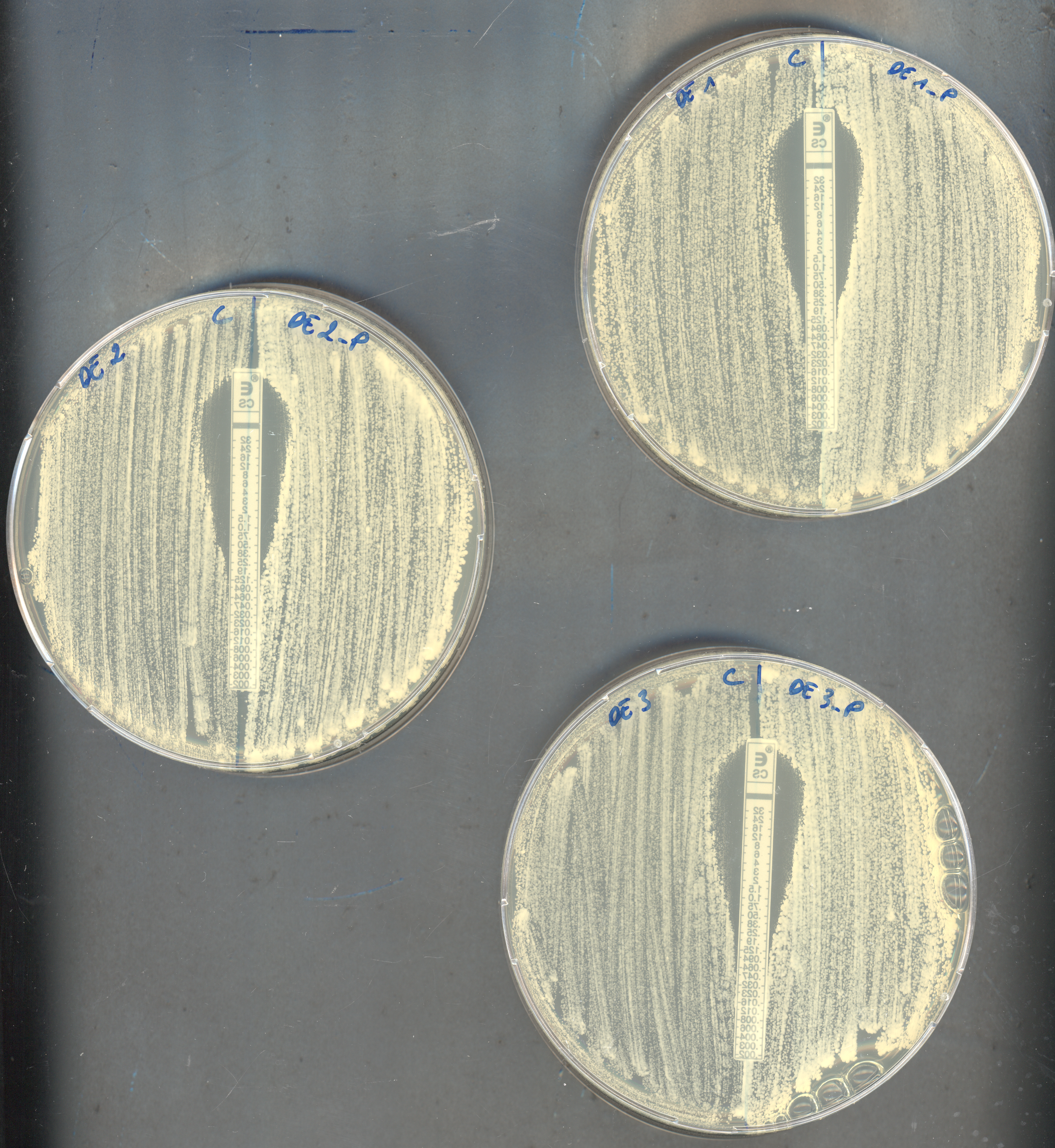

Supplement: Supplementary file 21 — Figure EV3 Source Data [file 44319_2026_702_MOESM21_ESM.zip › Figure EV3_SourceData/EV3_C&D_SourceData/Images/48hr/cerevisiae_Casp_2.TIFF]

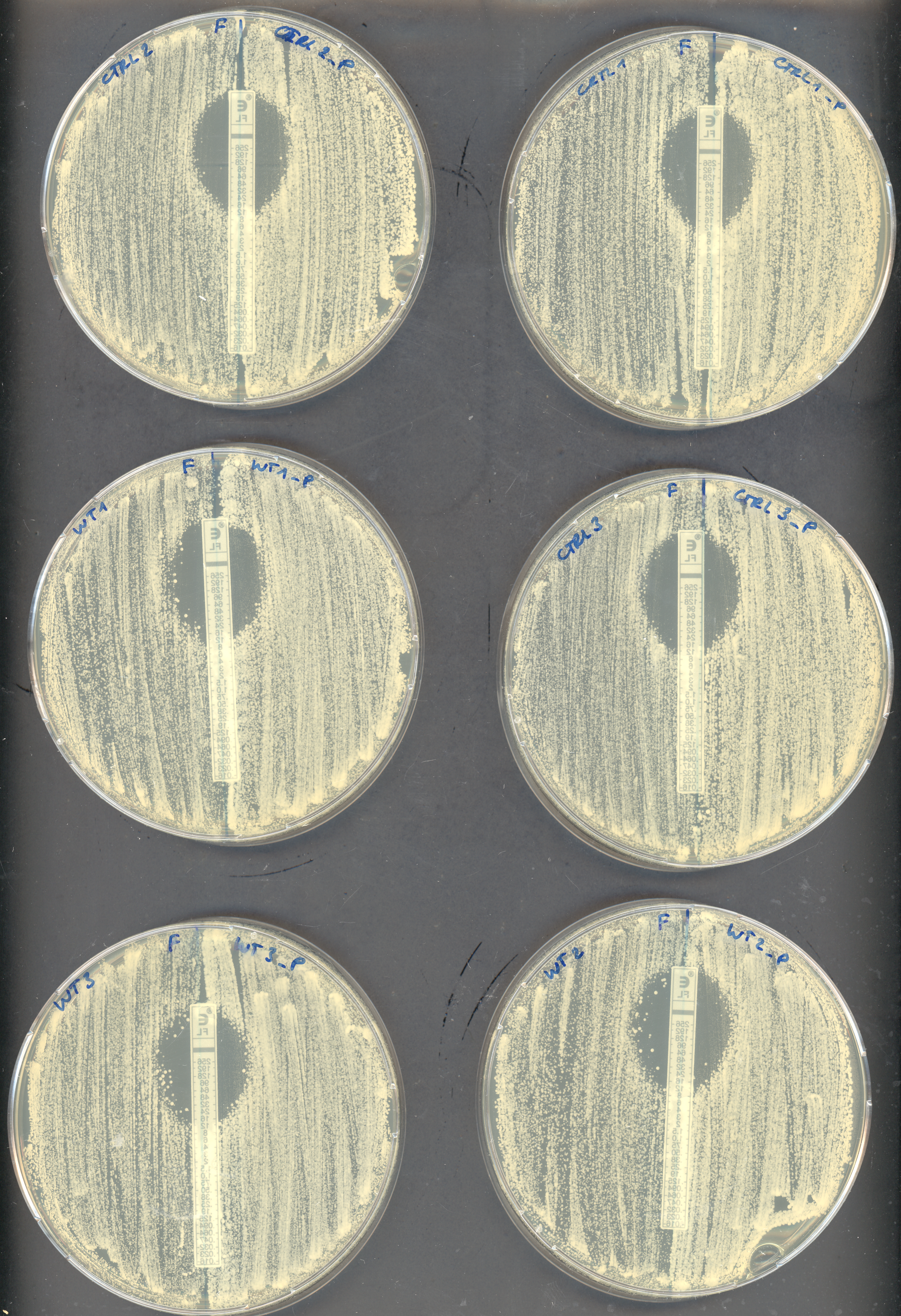

Supplement: Supplementary file 21 — Figure EV3 Source Data [file 44319_2026_702_MOESM21_ESM.zip › Figure EV3_SourceData/EV3_C&D_SourceData/Images/48hr/cerevisiae_FLC_1.TIFF]

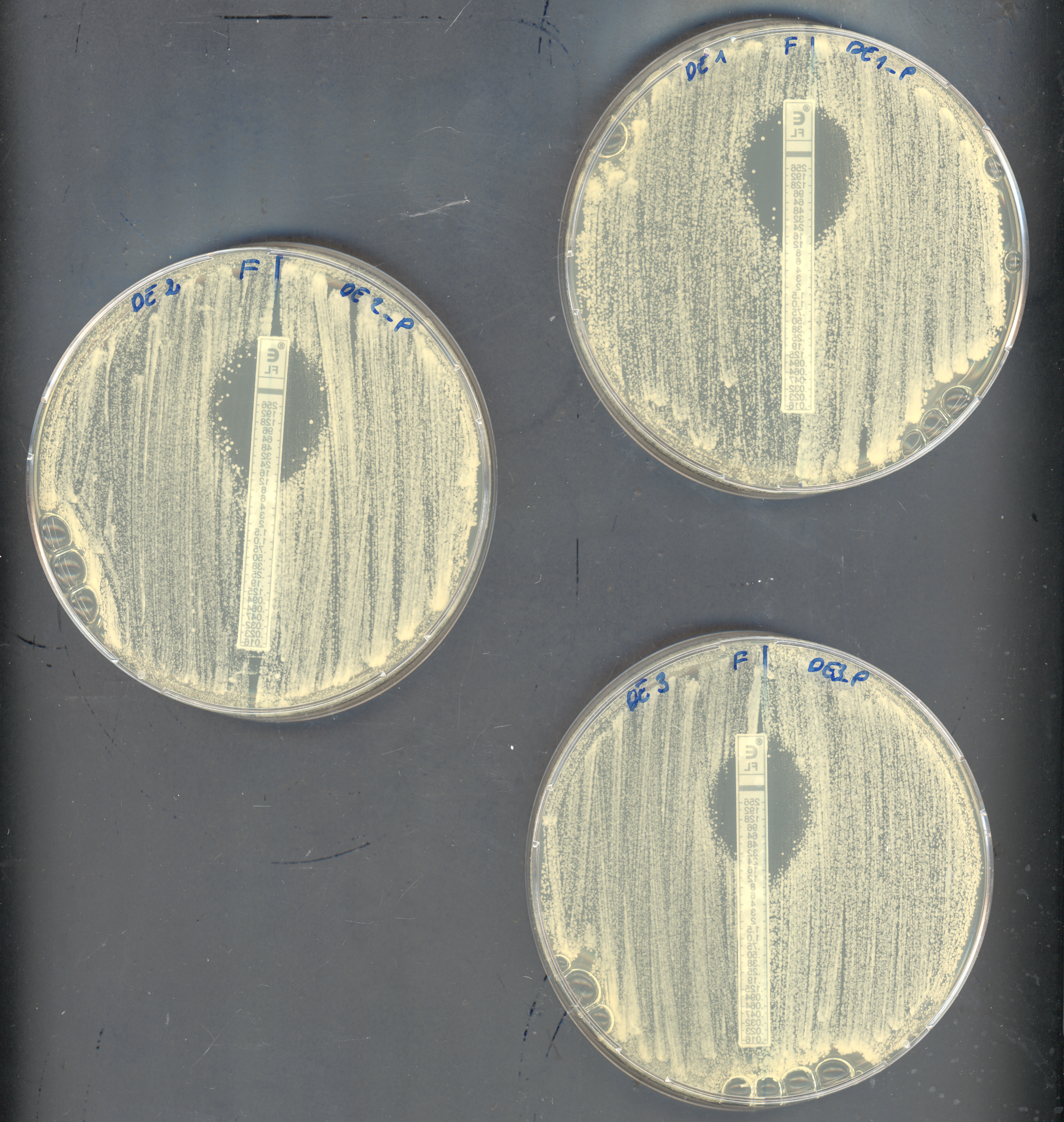

Supplement: Supplementary file 21 — Figure EV3 Source Data [file 44319_2026_702_MOESM21_ESM.zip › Figure EV3_SourceData/EV3_C&D_SourceData/Images/48hr/cerevisiae_FLC_2.TIFF]
