# Supplementary material for: Gim3 buffers and potentiates de novo mutations that affect fluconazole susceptibility in yeast
Source: EMBO Rep. 2026 Feb 17;27(6):1510–39. doi: 10.1038/s44319-026-00702-x (PMC13022404; doi:10.1038/s44319-026-00702-x)
Supplement: Supplementary file 23 — Expanded View Figures [file 44319_2026_702_MOESM23_ESM.pdf]

Expanded View Figures

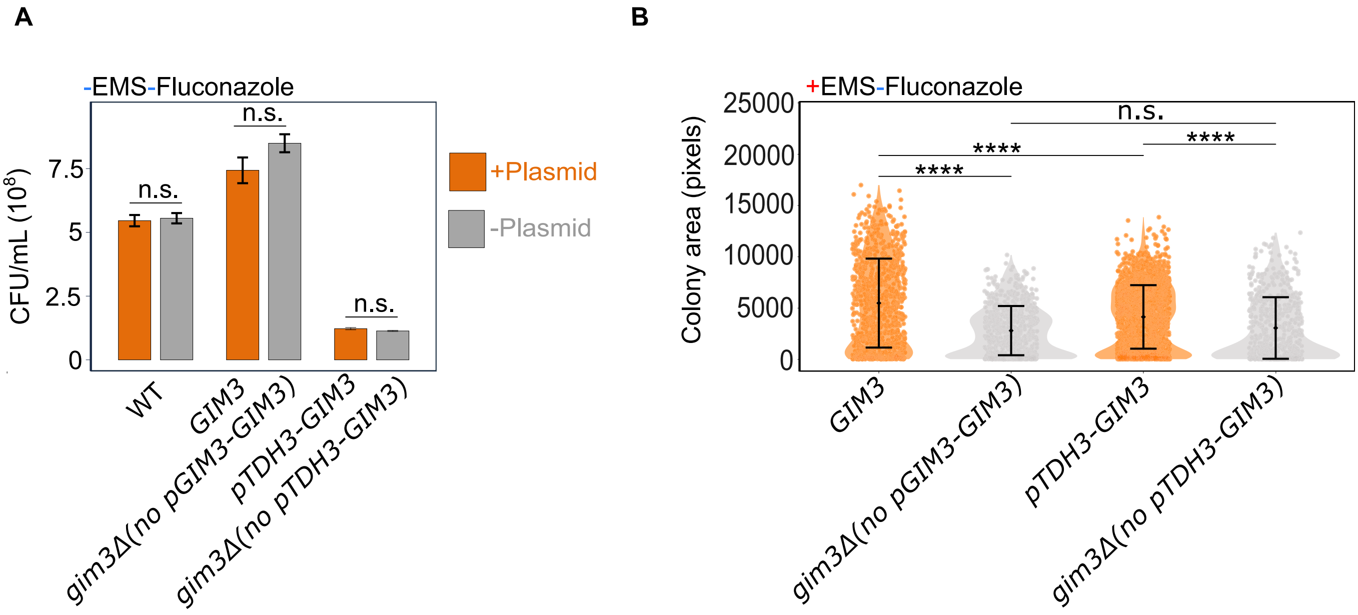

Figure EV1. Growth and colony size of yeast strains with and without GIM3 under non-stress conditions.

(**A**) Growth comparison in non-stress conditions. Yeast strains (*S. cerevisiae* BY4741 background) with and without the *GIM3* gene deletion were grown in SC medium at 30 °C. Colony-forming units per milliliter (CFU/ml) were measured after incubation. Data represent the mean ± s.e.m. of $n = 10$ technical replicates. Statistical significance was assessed using a two-sided generalized linear model (GLM; family = quasi-Poisson, link = log). *P* values corresponding to the figure, from left to right, are 0.999, 0.120, and 0.994. (**B**) Colony size differences between EMS-treated yeast strains (*S. cerevisiae* BY4741 background) with and without the *GIM3* gene under non-stress conditions (SC medium at 30 °C). Error bars represent mean colony size ± s.e.m. of n = 10 technical replicates from a single experiment. Statistical significance was assessed using a two-sided pairwise Wilcoxon Rank-Sum Test. Exact *P* values *GIM3* vs. *Δgim3 (no pGIM3-GIM)* = 1.13×10^{-28}, *pTDH3_GIM3* vs. *Δgim3 (no pTDH3-GIM3)* = 5.04×10^{-19}, *GIM3* vs. *pTDH3_GIM3* = 3.20×10^{-11}, *Δgim3 (no pGIM3-GIM3) vs Δgim3 (no pTDH3-GIM3)* = 0.889. Statistical significance was considered at *P* value < 0.05. Exact genotypes of the strains used in this figure are provided in Table EV7. Significance thresholds: n.s. = not significant, $^*P < 0.05$, $^{***}P < 0.001$, $^{****}P < 0.0001$. Source data are available online for this figure.

© The Author(s)

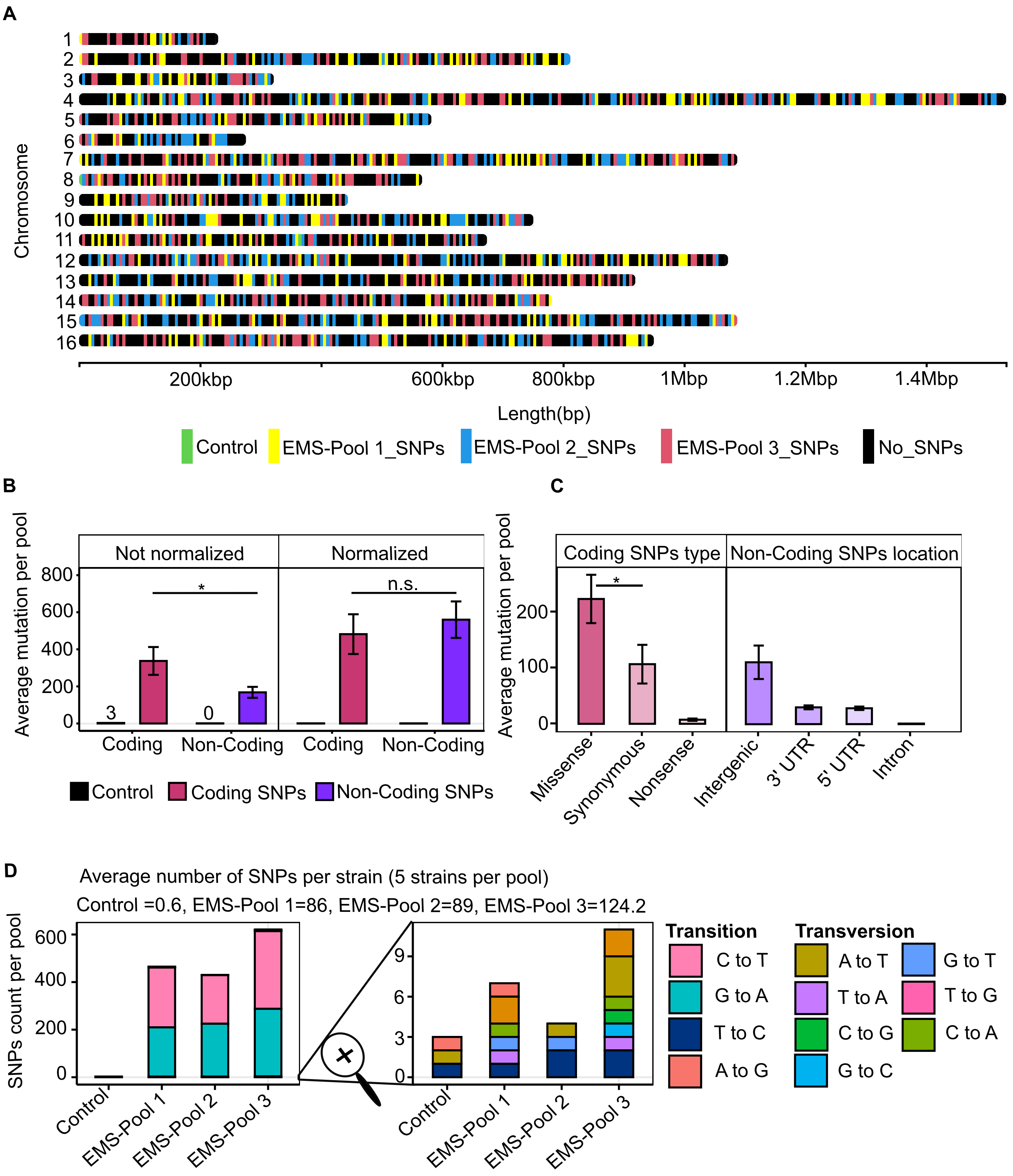
A
Chromosome
1
2
3
4
5
6
7
8
9
10
11
12
13
14
15
16
200kbp
600kbp
800kbp
1Mbp
1.2Mbp
1.4Mbp
Length(bp)
Control
EMS-Pool 1_SNPs
EMS-Pool 2_SNPs
EMS-Pool 3_SNPs
No_SNPs
B
Average mutation per pool
Not normalized
Normalized
800
600
400
200
0
*
n.s.
3
0
Coding
Non-Coding
Coding
Non-Coding
Control
Coding SNPs
Non-Coding SNPs
C
Average mutation per pool
Coding SNPs type
Non-Coding SNPs location
200
100
0
*
Missense
Synonymous
Nonsense
Intergenic
3' UTR
5' UTR
Intron
D
Average number of SNPs per strain (5 strains per pool)
Control =0.6, EMS-Pool 1=86, EMS-Pool 2=89, EMS-Pool 3=124.2
SNPs count per pool
600
400
200
0
9
6
3
0
Control
EMS-Pool 1
EMS-Pool 2
EMS-Pool 3
Transition
C to T
G to A
T to C
A to G
Transversion
A to T
T to A
C to G
G to C
G to T
T to G
C to A

 © The Author(s)

◀ **Figure EV2. Comprehensive analysis of mutations induced by EMS random mutagenesis in *S. cerevisiae* BY4741.**

(**A**) Distribution of EMS-Induced Mutagenesis Across Chromosomes: Mutations are distributed across different chromosomes within the randomly mutagenized pool. Yellow, blue, and pink colors represent the mutations found in their respective pools, with each pool comprising 5 clones. Green indicates the control pool, where only 3 mutations were identified. Black represents the chromosomes themselves. All mutations shown are single-nucleotide polymorphisms (SNPs). (**B**) The average number of SNPs in coding vs. non-coding genome regions: Left: The average number of SNPs across all pools of EMS-treated clones, categorized by coding and non-coding regions of the genome. Right: The normalized average number of SNPs, adjusted for the coding (~70%) vs. non-coding (~30%) proportions of the BY4741 genome. A significant difference was observed in SNP counts between coding and non-coding regions before normalization (*t* test, *P* value = 0.045). However, after normalization to the genome proportions, no significant difference was found (*t* test, *P* value = 0.4), highlighting the randomness of EMS mutagenesis. Data represent the mean ± s.e.m. of $n = 3$ independent EMS-mutagenized pools. (**C**) Analysis of coding and non-coding mutations: Left: The average number of coding mutations across EMS-treated pools. Missense mutations are significantly higher than synonymous mutations (*t* test, *P* value = 0.021). Data represent the mean ± s.e.m. of $n = 3$ independent EMS-mutagenized pools. Right: The average number of non-coding SNPs across EMS-treated pools and their distribution across the genome. (**D**) Mutation types and frequency: The types of mutations observed in each pool, with a majority of C to T and G to A transitions. The left panel zooms in on other less common mutations observed at lower frequencies. On average, each strain within a pool exhibited between 86 and 124 mutations per genome. From the MT1 strain listed in Table EV7, we generated an untreated control pool and 3 independent EMS-mutagenized pool. Source data are available online for this figure.

© The Author(s)

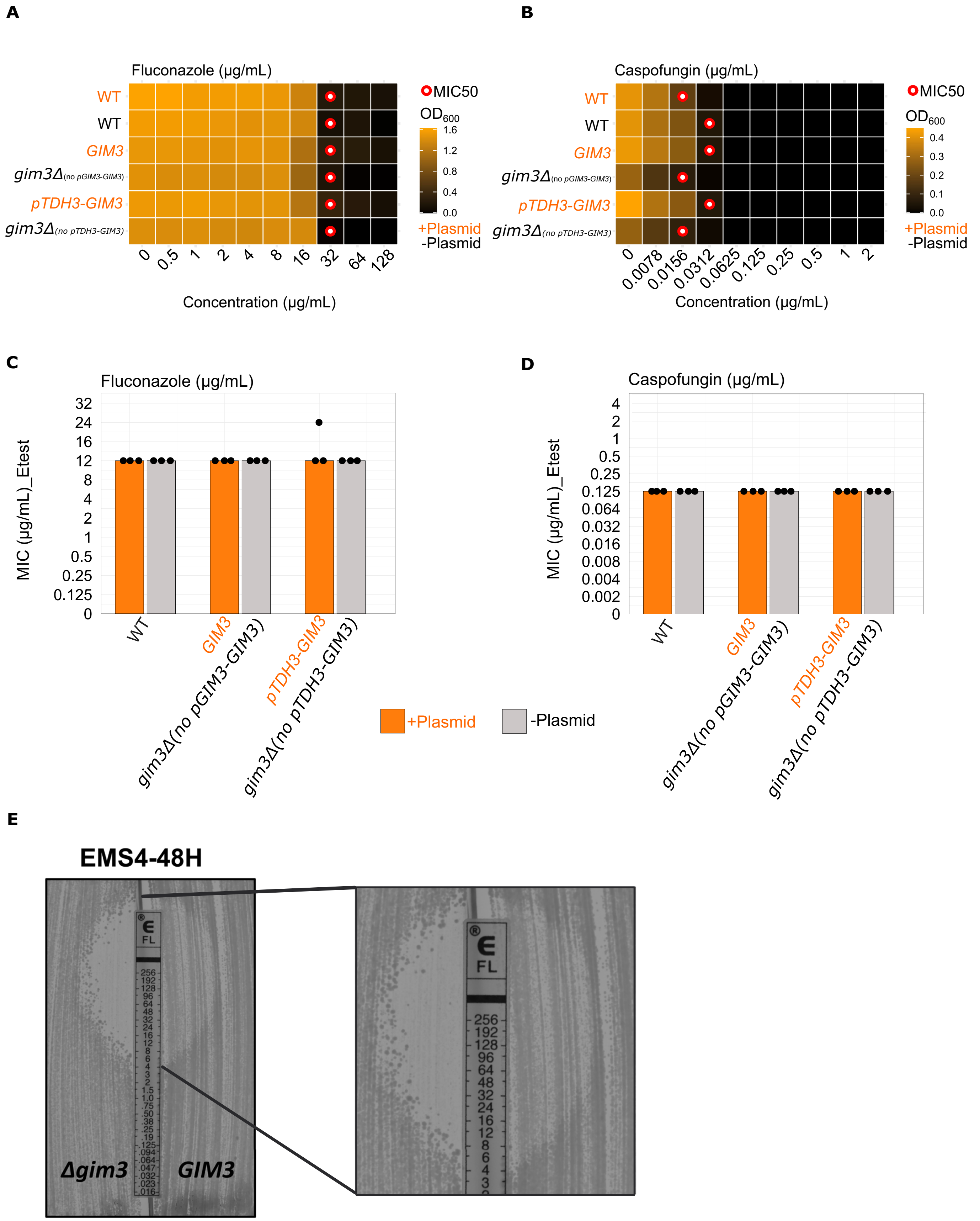
A
Fluconazole (µg/mL)
WT
WT
GIM3
gim3Δ(no pGIM3-GIM3)
pTDH3-GIM3
gim3Δ(no pTDH3-GIM3)
0 0.5 1 2 4 8 16 32 64 128
Concentration (µg/mL)
MIC50
OD600
1.6 1.2 0.8 0.4 0.0
+Plasmid
-Plasmid
B
Caspofungin (µg/mL)
WT
WT
GIM3
gim3Δ(no pGIM3-GIM3)
pTDH3-GIM3
gim3Δ(no pTDH3-GIM3)
0 0.0078 0.0156 0.0312 0.0625 0.125 0.25 0.5 1 2
Concentration (µg/mL)
MIC50
OD600
0.4 0.3 0.2 0.1 0.0
+Plasmid
-Plasmid
C
Fluconazole (µg/mL)
MIC (µg/mL)_Etest
32 24 16 12 8 4 2 1 0.5 0.25 0.125 0
WT
GIM3
gim3Δ(no pGIM3-GIM3)
pTDH3-GIM3
gim3Δ(no pTDH3-GIM3)
+Plasmid
-Plasmid
D
Caspofungin (µg/mL)
MIC (µg/mL)_Etest
4 2 1 0.5 0.25 0.125 0.064 0.032 0.016 0.008 0.004 0.002 0
WT
GIM3
gim3Δ(no pGIM3-GIM3)
pTDH3-GIM3
gim3Δ(no pTDH3-GIM3)
E
EMS4-48H
Δgim3
GIM3
FL

 © The Author(s)

Figure EV3. *GIM3* does not significantly alter fluconazole MIC_{50} in *S. cerevisiae.*

(**A**, **B**) Heatmaps showing OD_{600} values from broth dilution assays (BDA) in the presence of increasing concentrations of fluconazole (**A**) and caspofungin (**B**) for wild-type (WT), *gim3Δ*, and *GIM3*-complemented strains (native promoter or *pTDH3-GIM3*). Orange indicates plasmid-containing strains (+Plasmid), gray indicates plasmid-free strains (−Plasmid). Red circles mark MIC_{50} values, defined as the lowest drug concentration that reduces OD_{600} by ≥50% after 24 h at 30 °C. (**C**, **D**) MIC values determined by Etest for the same strains, shown for fluconazole (**C**) and caspofungin (**D**). Each bar represents the MIC of three biological replicates after 48 h incubation at 30 °C; black dots indicate individual replicates. Across both BDA and Etest assays, *GIM3* deletion does not significantly alter fluconazole MIC. In contrast, for caspofungin, a modest decrease in MIC_{50} is observed in liquid BDA for *gim3Δ*, but not on solid media (Etest). (**E**) Etest MIC analysis of 35 EMS-derived strains identified as *GIM3*-dependent in Fig. 2B. Only 3 strains showed a higher MIC in the presence of *GIM3*, indicating *GIM3*-dependent resistance, while most strains had comparable MIC values with or without *GIM3*. Representative image is shown: the left panel shows a *gim3Δ* strain; the right panel depicts a strain carrying *GIM3*. Zone diameters (MIC) are similar, but residual growth ('trailing') within the zone is greater when *GIM3* is present. Complete MIC data are available in Table EV2. Exact genotypes of the strains used in this figure are provided in Table EV7. Source data are available online for this figure.

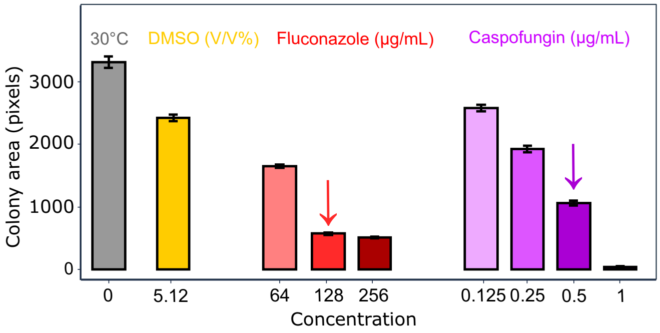

Figure EV4. Bar plot showing colony area (pixels) of the unevolved *S. cerevisiae* WT strain (BY4741) under different stress conditions.

Colony size was measured from plate images using the SGAtools online platform after 48 h of growth at 30 °C. Conditions include 0 µg/mL fluconazole (gray), 64, 128, and 256 µg/mL fluconazole (red gradient), 5.12% DMSO (yellow bar, used to dissolve 256 µg/mL fluconazole), and 0.125, 0.25, 0.5, and 1 µg/mL caspofungin (purple gradient). Bars represent the mean colony area from $n = 16$ technical replicates (colonies), black error bars indicate mean ± s.e.m. No statistical test was applied. Exact genotypes of the strains used in this figure are provided in Table EV7. Source data are available online for this figure.

 © The Author(s)
